# Novel pharmacological maps of protein lysine methyltransferases: key for target deorphanization

**DOI:** 10.1186/s13321-018-0288-5

**Published:** 2018-07-21

**Authors:** Obdulia Rabal, Andrea Castellar, Julen Oyarzabal

**Affiliations:** Small Molecule Discovery Platform. Molecular Therapeutics Program, Center for Applied Medical Research, CIMA, University of Navarra, Pio XII, 55, 31008 Pamplona, Spain

**Keywords:** Pharmacological similarity, Epigenetics, Protein lysine methyltransferase, Deorphanization, Polypharmacology, Protein–ligand interaction fingerprint, SET domain, SAM-competitive, Substrate-competitive

## Abstract

Epigenetic therapies are being investigated for the treatment of cancer, cognitive disorders, metabolic alterations and autoinmune diseases. Among the different epigenetic target families, protein lysine methyltransferases (PKMTs), are especially interesting because it is believed that their inhibition may be highly specific at the functional level. Despite its relevance, there are currently known inhibitors against only 10 out of the 50 SET-domain containing members of the PKMT family. Accordingly, the identification of chemical probes for the validation of the therapeutic impact of epigenetic modulation is key. Moreover, little is known about the mechanisms that dictate their substrate specificity and ligand selectivity. Consequently, it is desirable to explore novel methods to characterize the pharmacological similarity of PKMTs, going beyond classical phylogenetic relationships. Such characterization would enable the prediction of ligand off-target effects caused by lack of ligand selectivity and the repurposing of known compounds against alternative targets. This is particularly relevant in the case of orphan targets with unreported inhibitors. Here, we first perform a systematic study of binding modes of cofactor and substrate bound ligands with all available SET domain-containing PKMTs. Protein ligand interaction fingerprints were applied to identify conserved hot spots and contact-specific residues across subfamilies at each binding site; a relevant analysis for guiding the design of novel, selective compounds. Then, a recently described methodology (GPCR-CoINPocket) that incorporates ligand contact information into classical alignment-based comparisons was applied to the entire family of 50 SET-containing proteins to devise pharmacological similarities between them. The main advantage of this approach is that it is not restricted to proteins for which crystallographic data with bound ligands is available. The resulting family organization from the separate analysis of both sites (cofactor and substrate) was retrospectively and prospectively validated. Of note, three hits (inhibition > 50% at 10 µM) were identified for the orphan NSD1.

**Electronic supplementary material:**

The online version of this article (10.1186/s13321-018-0288-5) contains supplementary material, which is available to authorized users.

## Background

Protein lysine methyltransferases (PKMTs) are proteins that methylate histone and nonhistone proteins by catalyzing the transfer of the methyl group of the cofactor S-adenosyl-l-methionine (SAM or AdoMet) to a lysine residue of its corresponding substrate and yielding S-adenosyl-l-homocysteine (SAH or AdoHcy). PKMTs consist of two classes based on the structure of their catalytic domain (fold): SET domain-containing (class V) and Rossmann-like alpha/beta PKMTs (class I methyltransferases, MTs) [1]. The only representative member of the latter is the DOT1L PKMT, which is therefore structurally more related to protein arginine methyltransferases (PRMTs). There are 50 human SET domain-containing PKMTs (obsolete UniProt entry Q6ZW69, corresponding to ASHH1, is not human) [2, 3]. This conserved SET domain consists of approximately 130 amino acids folded into a series of β strands and a structurally variable insert that surround a canonical pseudoknotmotif at the C-terminal segment of SET [4, 5]. This pseudoknotmotif contains the highly conserved NHS/CxxPN motif, where x is any amino acid, and is in close proximity with the loop having the second highly conserved ELxF/YDY motif (the last Y being the catalytic residue). A third highly conserved motif is the GxG triplet at the N-terminal region. The core SET domain forms part of the catalytic domain and is flanked by non-conserved set of regions like the i-SET and post-SET (cSET) domain that form the binding groove for the substrate peptide. The cofactor binds at a different pocket, also partially contributed by the post-SET domain and connected by a narrow hydrophobic binding channel to the substrate binding site. Depending on the PKMT subfamily, SET domains can also be flanked by Pre-SET, N-SET, MYND and CTD domains [5].

Clinical evidence supports the implications of these enzymes in cancer and many other human diseases, including inflammation, brain disorders, metabolic and cardiovascular diseases, what has attracted considerable interest in the development of selective small molecule inhibitors targeting PKMTs [3, 6]. A parallelism between the current status with PKMT inhibitor design and that for kinases 20 years ago was established [7], although here the main current challenge is that here some PKMT subfamilies remain unexplored. At sight of reported selectivity profiles for currently available chemical probes and advanced compounds in clinical trials, achieving selectivity within the PKMT family seems a trivial task compared to the situation in the field of kinases, although the high structural conservation of substrate and cofactor binding sites challenges the design of selective inhibitors. Nevertheless, selectivity profiling of advanced compounds is expensive and unaffordable for academic groups. Here, rationale approaches that incorporate information on ligand recognition and that go beyond traditional sequence-based relationships between targets might be helpful to guide the identification of surrogate ligands for unexplored PKMTs and/or to prioritize targets for selectivity screening. In this sense, previous efforts with well-established therapeutic targets include ligand-based organization of GPCRs [8], protein–ligand interaction fingerprint-based clustering of kinase complexes [9] and cavity analysis of serine proteases [10] and epigenetic inhibitors [11], to mention a few. Relationships between different epigenetic families beyond PKMTs were recently explored on the basis of the chemical structures of their reported inhibitors [12]. An inconvenient of these approaches is that they either rely on a vast number of available ligands or are restricted to proteins with crystallographic structures (at least in the *apo* form for cavity analysis). A novel methodology, originally named GPCR-CoINPocket, appeared in 2017 that transfers patterns of ligand–residue interactions to sequence-based comparisons of proteins to deorphanize class A GPCRs [13].

Here, we propose a novel pharmacological organization of PKMTs according to the experimental interactions detected in both, the SAM and the substrate binding sites, by using an adaptation of the GPCR-CoINPocket methodology for the analysis of PKMTs (hereafter referred to as PKMT-CoINPocket). Unexpected similarities between PKMTs emerged from the resulting family arrangements of the separate analysis of both sites that were retrospective and prospectively validated, leading to the identification of three hits targeting the orphan NSD1.

## Methods

### PDB compilation

**Fig. 1:**
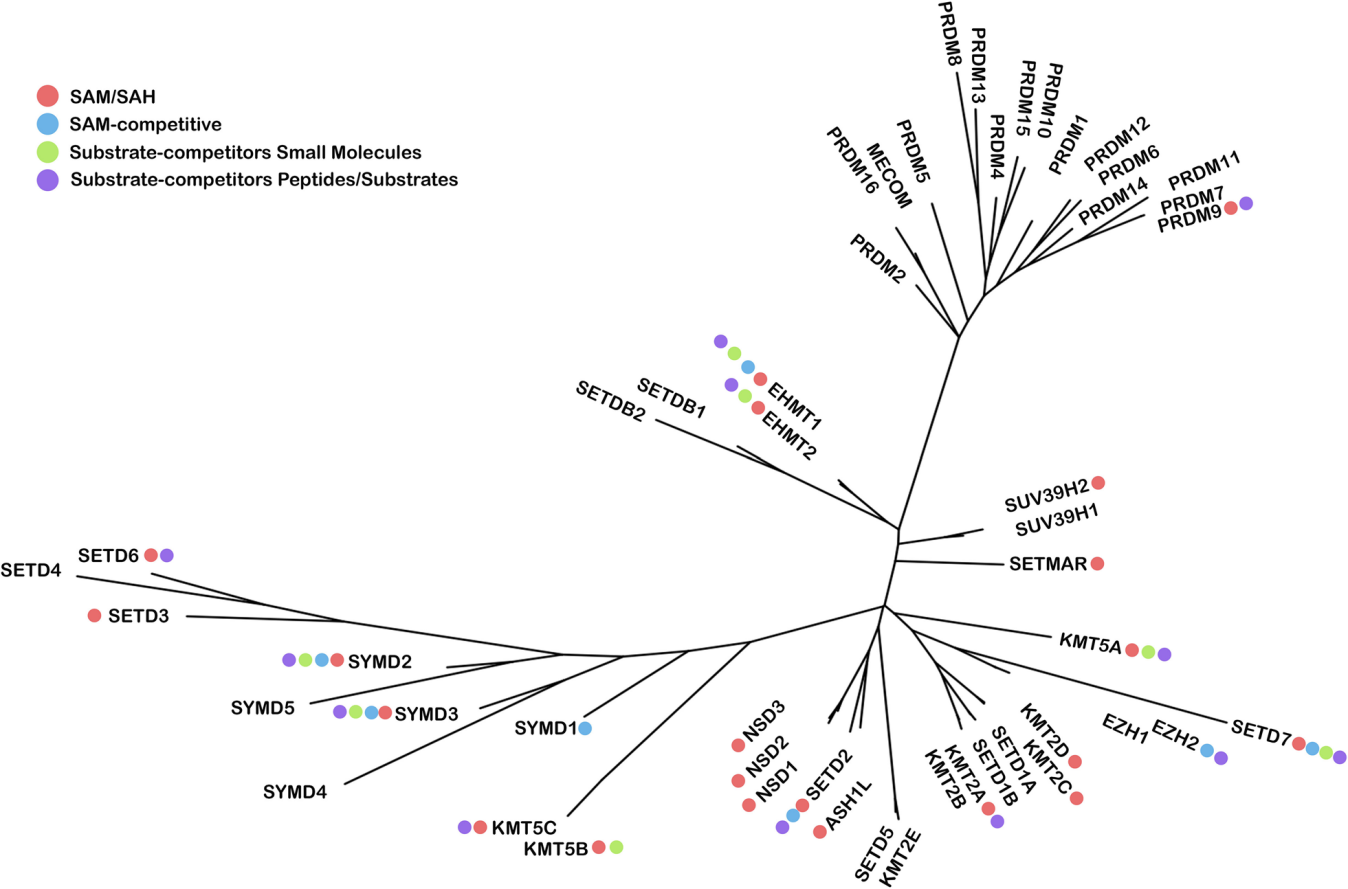
Phylogenetic tree of 50 SET domain-containing PKMTs with circles indicating co-crystallized ligands

Given the diversity of alternative names for methyltransferases [14], sequence names follow the HUGO Gene Nomenclature Committee standard gene names [15]. For each PKMT, we retrieved all the PDB entries (as for August 2017) [16]. Crystal structures lacking the methyltransferase (MT) domain or crucial residues in the cofactor or substrate binding cavities and *apo* structures were excluded. A total of 104 PDB entries, representative of 23 PKMTs, were compiled (Additional file 1: Table S1). In 101 out of the 104 PDB entries, the cofactor binding site is occupied by either SAM (41), SAH (41), sinefungin inhibitor (a nucleoside derivative of SAM, 6), other nucleoside-based SAM-like inhibitors (11) or non-nucleoside based small molecules (2). Of note, for the cofactor-binding site analysis we considered all crystals, independently of whether they had cofactors alone or also ligands in the substrate cavity. For the substrate binding cavity, 32 complexes are co-crystallized with small molecule substrate-competitive inhibitors which are representative of 7 different PKMTs: EHMT2, EHMT1, SETD7, KMT5A, KMT5B, SMYD2 and SMYD3. The phylogenetic tree in Fig. 1 represents the distribution of available crystals depending on bound ligands for all 50 SET-containing PKMTs. The corresponding chemical structures of small molecule inhibitors and biochemical profiling are given in Additional file 1: Table S2. Compound MTF003, (PDB entry 5WCG), is a bisubstrate SMYD2 inhibitor, so it was contemplated in the analysis of both binding pockets (resulting in 33 complexes for the substrate cavity). There are four pairs of PDB entries that share the same chemical structure bound to EHMT1 and EHMT2 (5TTG/5TTF; 5TUZ/5TUY; 5VSD/5VSC and 5VSF/5VSE). Moreover, for the pair 5TTG/5TTF structures, the X-ray resolved ligand structure does not match the original structures and differs between them, so the number of structurally different substrate-competitive ligands is 30. In any case, the 33 PDB entries were kept for protein–ligand interaction analysis of the substrate binding site. Most complexes correspond to human PKMTs, with the exception of 5 (*mus musculus*) and 1 (*Homo sapiens Anolis carolinensis*) complexes. With the exception of PRDM9 (86.8%), the remaining PKMTs have > 90% sequence identity with its human homolog (Additional file 1: Table S1). As PRDM9 crystal structure is the only representative crystal among PRDMs (Fig. 1), it was kept for analysis.

### PDB preparation

Downloaded PDBs were manually processed with MOE 2016.0801 to correct relevant residues with missing atoms, missing residues in small gaps and wrong ligand connectivity. Irrelevant ligands and waters were removed. Ligands were protonated at pH = 7. Only one chain per PDB entry was kept.

### Alignment

The performance of protein–ligand interaction fingerprints and alignment-based recognition of pharmacological similarities mostly relies on the quality of the alignment that captures similarities in the binding site of the analyzed ligands. Structural superposition of the 104 PDB entries based on a full length global sequence alignment downloaded from ChromoHub database [17, 18] did not reflect the similarities and relationships at the cofactor binding site (Additional file 1: Figure S1) [5, 19]. Thus, the ClustalW alignment of the MT domain (alone) of PKTMs at the ChromoHub database was finally chosen [17, 18]. This alignment was manually refined in MOE to include some single missing residues at the end of the MT domain that matched residues for other MTs present in the alignment or small obvious misaligned gaps (Additional file 2). Then, each of the 104 PDB entries were aligned and superposed with MOE against the sequence alignment. Here, the degree of overlap between SAM/SAH/SAM-competitive inhibitors and peptide substrates (Additional file 1: Figures S2 and S3) strongly supports the goodness of the alignment: mean RMSD among all SAH molecules of 0.26 ± 0.28 Å, taking SAH conformation in EHMT2 PDB entry 2O8J as reference, and of 0.30 ± 0.30 Å for the SAM conformations compared to that of SAM in SUV39H2 PDB entry in 2R3A. Also, the RMSD of superposition between the 101 structures complexed with SAM/SAH and analogues is low, especially for gapless residues in the alignment (< 2 Å, Additional file 1: Figure S4). Residue UID numbers were transformed into position alignment numbers. Additional file 1: Tables S3, S4 have the corresponding translation for relevant PKMTs discussed below.

### Protein ligand interaction fingerprints (PLIF)

Protein ligand interaction fingerprints (PLIF), as implemented in MOE 2016.0801 [20] were generated for each complex, using default settings of minimal and maximal contact energies. Two different analyses were run for each binding pocket (101 cofactor bound complexes for 23 PKMTs and 33 substrate bound complexes covering 7 PKMTs). For analysis of the interactions, we counted the number of PLIF-detected interactions that occur according to its type of interaction: hydrogen bond donor (HBD), hydrogen bond acceptor (HBA), ionic, surface contacts and arene attraction (these two last corresponding to hydrophobic interactions). For HBD, HBA and ionic interactions, if the ligand establishes multiple interactions with a given residue, the interaction was counted only once and no matter if the contact is established with the side chain or the backbone of the protein.

### PLIF clustering

For each set of complexes, we carried out an all-against-all comparison by calculating the pairwise Tanimoto coefficient between any two PLIF values. The R software [21] was used to cluster and draw the hierarchical tree using the average linkage clustering method (function *hclust*) and the PLIF similarity (previous conversion to distance).

### FCFP_4-based ligand similarity

The Tanimoto pairwise similarity for the 30 substrate-competitive inhibitors in Additional file 1: Table S2 was calculated using FCFP-4 [22] as implemented in Pipeline Pilot [23].

### Implementation of PKMT–CoINPocket

A workflow similar to that described by Ngo et al. [13] for determining GPCR-CoINPocket score was followed that consists of the following steps: (1) Determination of ligand contact-strength profiles as calculated with BaSiLiCo [24–26] and (2) Sequence-based comparison of PKMT-binding sites using the calculated ligand contact-strength profiles. For the first part, ICM binary files prepared with ICM software [27, 28] were inputted to BaSiLico. Given a ligand-protein complex, for each pair or non-hydrogen ligand and protein atoms separated by interatomic distance *d*, its contact strength is a function of interatomic distance and distributed within range of 0–1, with linear decrease from 3.23 to 4.63 Å. If *d* > 4.63 Å, the two atoms are not considered to be in contact. In the original paper describing GPCR–CoINPocket methodology [13], only side chain atom contacts were considered in the final consensus fingerprint. Here, because of relevant interactions between backbone atoms of PKMTs with ligands (see “Results and discussion”), the total contact strength *T* was considered. For a particular PKMT-binding site, if different complexes were available, all separate complexes were aggregated into a single ICM file and BaSiLiCo was run with *ensemble mode* to avoid redundancy in the contact patterns. Extended TSV files with information on the contact fingerprint were exported for each PKMT-binding site complex (hereafter referred to as the *projected binding site positional fingerprint vector*, following original GPCR–CoINPocket nomenclature). Then, given a sequence alignment of proteins, a vector or pairwise per-residue similarities was calculated as:

$$S_{ij} = M_{ij}/\sqrt {(M_{ij} \times M_{ij} } )$$

where *i* and *j* are amino acids at a given position of the alignment between two sequences and *M* is the Gonnet [29] residue comparison matrix. This calculation was obtained with MOE using a customized SVL script that writes a CSV file that stores, per each residue in the reference sequence, its non-normalized Gonnet coefficient against every other sequence in the alignment. These CSV files were processed with Pipeline Pilot to carry out PKMT–CoINPocket calculation. Here, the per-residue Gonnet similarities (*S*_*ij*_) between a couple of sequences *seq1* and *seq2* in the alignment were multiplied, element-wise by the projected binding site positional fingerprint vector obtained for each of the proteins for which contact strengths were calculated (i.e. proteins for which there is crystallographic information available of protein-ligand complexes). Then, Gonnet_PFP_L_, the similarity between *seq1* and *seq2* for a given projected binding site positional fingerprint vector of protein *L* was calculated as the sum of this vector over all residues. Note that due to the different lengths of the PKMTs (and alignment gaps), not all contact residues could be propagated. Then, Gonnet_PFP_L_ values were standardized into Z-scores for each binding-site fingerprint and the final profiled PKMT–CoINPocket score for a pair of proteins *seq1* and *seq2* in the alignment was calculated as the average of the Z-scores across the different binding profiles (23 and 7 for cofactor- and substrate-binding sites, respectively). Finally, to convert PKMT–CoINPocket scores to distances, the respective value was first normalized to be in the range of 0 to 1. A PKMT-CoINPocket score was calculated for each binding site. Due to the nature of the PKMT-CoINPocket approach, distances are fully dependent on the set (proteins or binding site) under study, and comparisons cannot be directly translated among different sets. For comparison purposes, the classical Gonnet similarity matrix was separately calculated for all residues within 4.5 Å of each set of ligands (co-factor and substrate sites, separately).

### PKMT–CoINPocket clustering and trees

The matrix of normalized PKMT–CoINPocket scores for all pairwise comparisons of sequences in the alignment was converted into distances (1-corresponding similarity score) and used to cluster PKMTs using the unweighted pair group method with arithmetic mean (UPGMA) algorithm [30], as implement in R [21]. Dendrograms obtained from clustering were saved as Newick files and trees were obtained with iTOL [31].

### CheMBL data set compilation for retrospective validation

ChEMBL [32, 33] database was queried (as in September 2017) to retrieve ligands with inhibitory activity against any of the different PKMTs in Additional file 1: Table S1. Only data for human proteins and activities given as IC_50_, inhibition, K_d_ or K_i_ were retained. A total of 1712 data points were retrieved, associated to 908 different compounds. However, only for 43 out of the 908 compounds, there is available data on more than one single target. Allosteric compounds, compounds without a well-defined binding site (e.g. chaetocin and derivatives potentially binding to cysteine-rich regions) [34, 35], inactive compounds against all PKMTs and compounds already in Additional file 1: Table S2 or their very close analogues with similar selectivity profiles were discarded. After this refining, only the substrate-competitive inhibitor Cyproheptadine and the SAM-competitive EZH2 inhibitor GSK343 remained. Lastly, in order to enlarge the data set, 5 additional inhibitors with selectivity profiles were rescued from literature [3]. The chemical structures and biochemical profiles of these 7 structures are given in Additional file 1: Table S5.

### Inhibitory activity assays for prospective validation

The synthesis and biological activity of the three proprietary compounds CM-272, CM-679 and CM-986 has been reported [36–39]. Chemical structures and IC_50_ values against EHMT2 (G9a) and DNMT1 are given in Fig. 12a. Inhibitory assays against SETD2, KMT2A, KMT5C, NSD1 and NSD2 were performed by Eurofins (https://www.eurofins.com/) with radioligand binding assays ([3H] SAM and different substrates: nucleosome (SETD2, NSD1, KMT5C), core histone (NSD2) and histone H3 full length (KMT2A) at 10 µM and 100 µM and tested in duplicates.

## Results and discussion

This section is structured as follows. For each binding site (cofactor and substrate), an updated analysis of detected interactions of bound ligands is firstly discussed, with emphasis on novel (un)conserved interaction patterns undisclosed in previous analysis because of the higher number of available crystal structures. Second, the results of the PKMT-CoINPocket approach are presented, to end up with the validation cases.

### Cofactor binding site

#### Analysis of bound ligand interactions

**Fig. 2:**
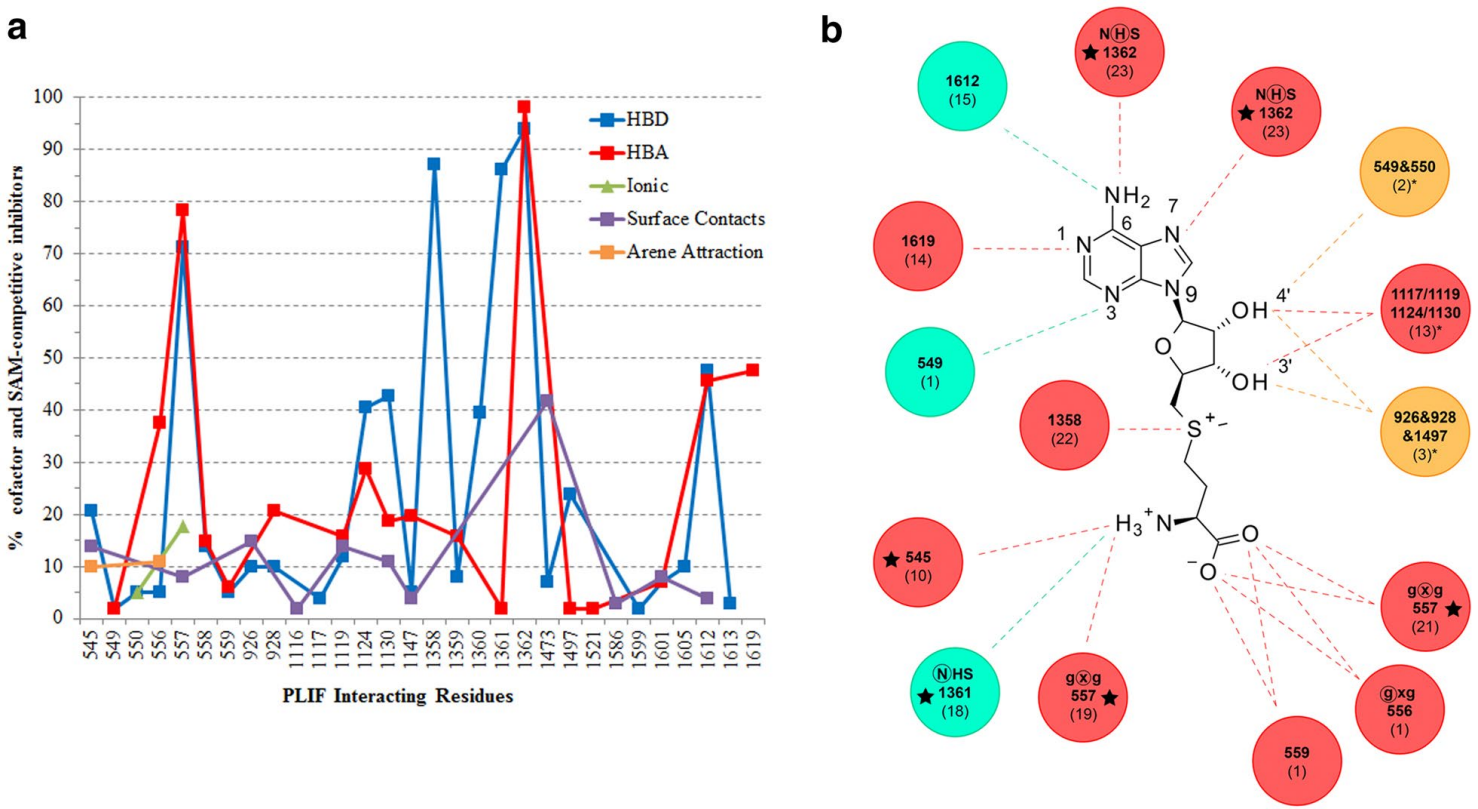
**a** Percentage of cofactor and SAM-competitive inhibitors that interact with a given PLIF interacting residue according to interaction type. **b** Summary of detected HB interactions for SAM. Circles represent PLIF interacting residues: numbers correspond to residue alignment position (conserved residues are shown, if applicable) and (numbers) correspond to the number of different PKMTs for which this interaction was detected. Red, blue and orange colors are for backbone, side chain or both types of HB contacts, respectively. The network of six HBs described by Campagna-Slater et al. [19] is labeled with stars: (1) N7 of SAM as HBA with the highly conserved His (Cys in PRDM9) of the NHS/CxxPN motif (position 1362); (2) amine group of the adenine ring with this same residue; amine group of the methionine fragment acting as HBD (3) with the Asn (1361) of the NHS/CxxPN motif and (4) the x residue at position 557 of the conserved motif GxG and (5) the residue upstream of the GxG motif (545); (6) the carboxylate group of the methionine moiety acting as HBA with the x residue of the GxG motif (557)

A comprehensive study of the cofactor binding cavity (without ligands) of 10 SET PKMTs, 3 classical PRMTs and the non-SET PKMT DOT1L was done by Campagna-Slater et al. in 2011 using GRID maps [19]. Here, a network of six hydrogen bonds for the cofactor was established as a motif present in all studied SET PKMTs. Our analysis of explicit interactions for the 23 SET-domain containing PKMTs reveals that most of the 1320 residue-based contacts detected with PLIF correspond to hydrogen–bond interactions (86%), with a preference for the ligand being HBD (50.6%) versus HBA (35.4%). Hydrophobic interactions, quantified as the number of surface contacts and arene attraction, account for only 11% of the interactions. These contacts are widely distributed across a set of 30 residues in the alignment (hereafter referred to as *PLIF interacting residues*, Fig. 2a and details in Additional file 1: Table S6).

**Fig. 3:**
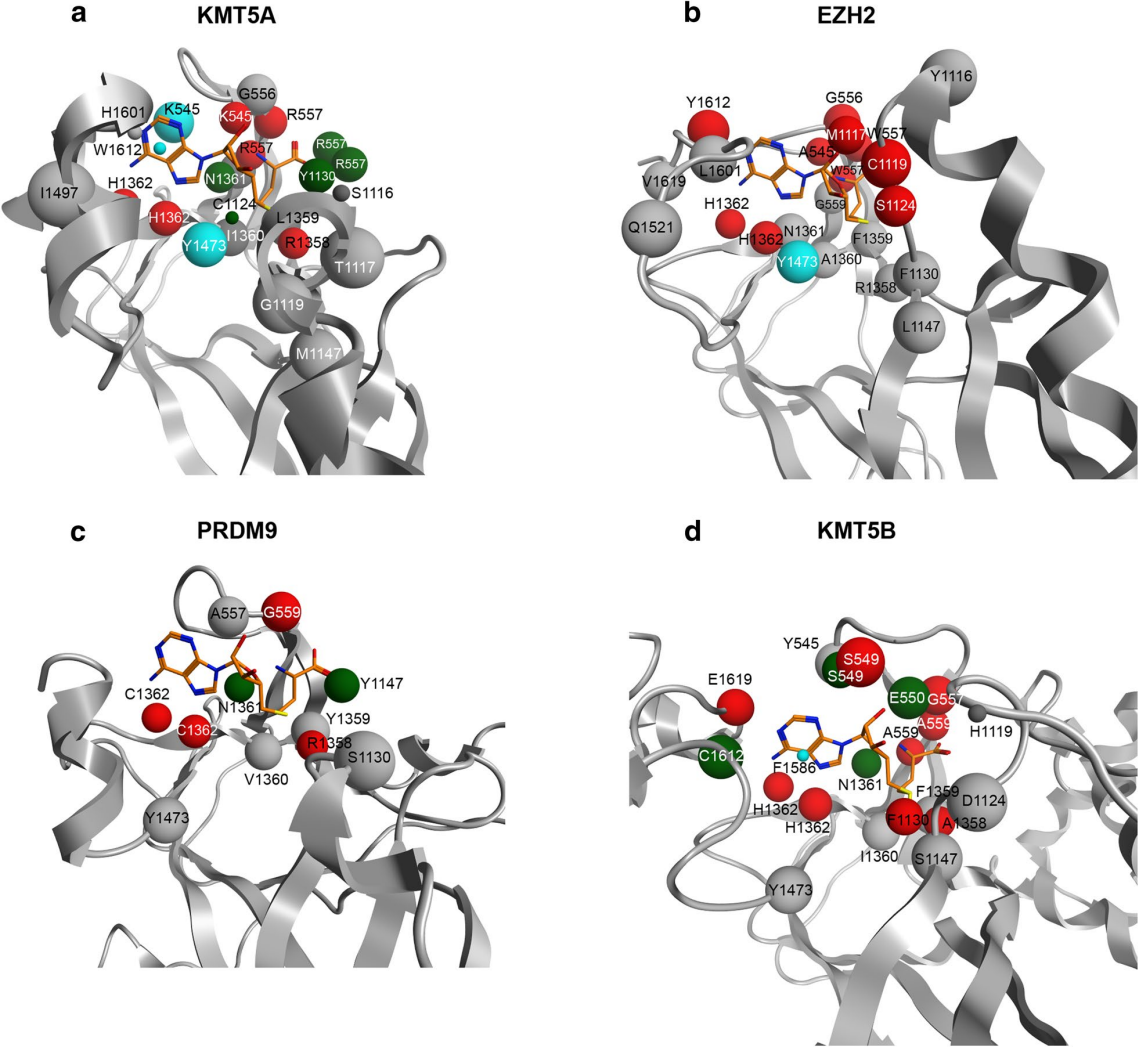
Mapping of PLIF interacting residues for 4 complexes of representative PKMTs complexed with either SAM or SAH. **a** KMT5A (PDB: 4IJ8; SAM); **b** EZH2 (PDB: 5HYN; SAH); **c** PRDM9 (PDB: 4C1Q; SAH) and **d** KMT5B (PDB: 5WBV; SAM). All 30 PLIF interacting residues for SAM-competitive inhibitors are shown, considering that the residue can be mapped in the alignment. Balls correspond to either backbone (red) or side chain (green) atoms establishing HB contacts or hydrophobic contacts (blue), respectively. Grey balls, marked at the C*α* atom, correspond to PLIF interacting residues for which the corresponding ligand does not establish any interaction

As summarized for SAM in Fig. 2b, not all the mentioned six hydrogen bonds are 100% conserved in all 23 SET PKMTs, especially with respect to the interaction of the amine group of the methionine, and more particularly with residue x at position 545 (GxG), which was detected in only 21% of the complexes and for 10 out the 23 PKMTs. KTM5A complexed with SAM exemplifies well the conservation of this HB interaction network as well as other hydrophobic interactions (Fig. 3a). In contrast, the PRDM9-SAH complex exhibits fewer explicit interactions, with the residue at position 559 (Gly257) replacing HB contacts at 557, so basically only the HB interactions of the adenine ring with residues at 1362 and 1361 are conserved (Fig. 3c). Examples for six additional PKMT complexes, representative of different patterns of interactions, are given in Additional file 1: Figure S5. From the viewpoint of non-nucleoside competitive inhibitors design, the two structures of EZH2 complexed with pyridone inhibitors (Additional file 1: Table S2) suggest that targeting the residue at position 557 of the GxG motif (Trp624 in EZH2) is more relevant than blocking the hydrogen-bond network of the adenine ring of SAM/SAH with residues NH (1361–1362) of the pseudoknotmotif of the SET domain (Asn688 and His689 in EZH2) (see individual 2D maps of interactions for the bound ligands, Additional file 1: Figure S6). More interestingly, our analysis of PLIF interacting residues reveals other highly conserved interactions between SAM/SAH cofactors and analogues that might be relevant for the design of SAM competitive inhibitors. Specifically, we mean the role of the N1 and N3 atoms of the adenine ring and the hydroxyl groups of the ribose ring of SAM/SAH and analogues. In 14 PKMTs, a bidentate hydrogen bond interaction is established between (1) the N1 of the adenine ring and the backbone of non-sequence conserved residue at alignment position 1619 of the C-terminal region and (2) the –NH2 group of the adenine nucleotide and the conserved Cys at 1612 (side chain contact) (Fig. 3d for KMT5B). These 14 PKMTs (KMT5B, KMT5C, EHMT1, EHMT2, SETMAR, SUV39H2, ASH1L, SETD2, NSD1, NSD2, NSD3, KMT2A, KMT2C, KMT2D) are representative of different subfamilies in the phylogenetic tree (Fig. 1). For EZH2, only the contact at Cys at 1612 is observed (Fig. 3b). Thus, this N1 contact is non-exclusive of PRMTs [19], but also present in many SET domains, and not only as a potential interaction HB hot spot for SET domains [19], but as a real contact that might difficult the design of PKMT over PRMT selective SAM competitive inhibitors. Concerning the N3 atom of the adenine ring, it is mainly involved in hydrophobic contacts. However, KMT5B presents a unique feature: it is the only PKMT HB bounded to N3 through the side chain of Ser205 (residue alignment position 549, Fig. 3d). Thus, in contrast with previous analysis, the potential HB hot spot around this N3 region is not restricted to PRMTs [19], but has been experimentally found for at least one PKMT. The hydroxyl groups of the ribose ring also establish key hydrogen bonds with many different residues. There are four possible patterns: (1) PKMTs without a direct HB interaction (but possibly water mediated, as observed in specific PDB entries such as 2RFI of EHMT1) with these hydroxyls (SUV39H2, KMT2D, KMT2A, KMT2C, PRDM9 in Fig. 3c); (2) PKMTs with the hydroxyl group on the 3’ carbon of the ribose HBD (EHMT2, EHMT1, SETMAR, SETD7, NSD2, SETD6, SETD3, KMT5A in Fig. 3a); (3) or HBD with the hydroxyl group on the 4’ carbon of the ribose (KMT5B in Fig. 3d, KMT5C) and (4) HBD interaction at both hydroxyl groups (EZH2 in Fig. 3b, SETD2, ASH1L, NSD3, NSD1, SMYD3, SMYD2, SMYD1). Again, from the smaller data set of complexes in the 2011 study, apparently this double interaction was exclusively predicted in SMYD structures, but current available complexes demonstrate a higher coverage for the different PKMTs subfamilies. Also, the PLIF interacting residue with these –OH groups is highly variable (Fig. 2b): despite some pattern conservation like residues at alignment position 1117, 1119, 1124 and 1130, there are residues that are particular to the SMYD subfamily (926, 928 and 1497) or KMT5B/KMT5C (549 and 550, respectively). Moreover, contacts can be established with either backbone or side chain atoms of these residues. Finally, other frequent contacts include surface interactions with catalytic Tyr at 1473 (15 PKMTs) and HB interactions between the sulphur atom and/or the methyl group of SAM with 1358 (22 PKMTs) (Fig. 2b).

In summary, the detailed analysis above demonstrates subtle differences in the interaction patterns of the 23 PKMTs with SAM/SAH, despite their low RMSD of superposition, and, more importantly, highlights certain patterns of conservation of interactions across the different subfamilies. These semi-qualitative conclusions become apparent when comparing the heat maps of RMSD of superposition of SAM and SAH conformations (41 molecules in each case, Additional file 1: Figures S7 and S9, respectively) with the pairwise PLIF-based similarities between their corresponding complexes (separately obtained, for SAM- and SAH-bound complexes in Additional file 1: Figures S8 and S10, respectively). While pairwise RMSD values are mostly < 0.5 Å, distances in interaction profiles range from 0 (same protein) to 1. These quantitative differences support the integration of information on protein-ligand contacts to the prediction of potential pharmacological neighbors. From the viewpoint of selective ligand inhibitor design, these structural differences suggest that it is possible to achieve selectivity within the SAM binding pocket, as demonstrated for SAM-competitive inhibitors in Additional file 1: Table S2.

#### Prediction of pharmacological similarities with PKMT-CoINPocket Model

**Fig. 4:**
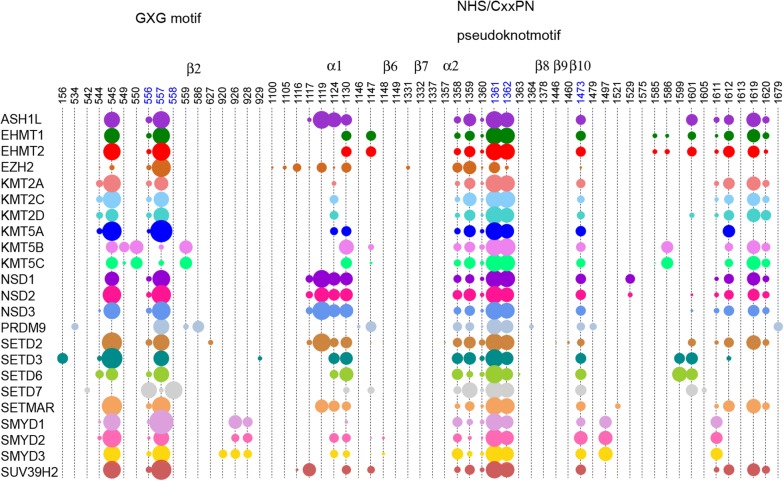
BaSiLiCO ligand contact map of SAM-competitive inhibitors of 23 PKMTs. The area of the circles reflects the relative strength of the ligand contact and residue position in the alignment

Clustering PKMTs using PLIF fingerprints to devise similarities among them is restricted to structurally solved proteins (< 50% of all SET-domain containing PKMTs; Additional file 1: Figure S11). Following the idea of GPCR-CoINPocket [13], the incorporation of information on the interactions established between ligands and key (un)conserved residues provides an interesting approach to map pharmacological similarities between proteins. As PLIF contacts are measured in terms of presence/absence of interactions, we finally opted for incorporating contact strength information as calculated with BaSiLiCo in the original GPCR-CoINPocket publication [13]. This resulted in the identification of a ‘cloud’ of 59 residue positions (Fig. 4) around the NHS/CxxPN pseudoknotmotif for the 23 PKMTs with available crystals. Despite differences in the number of interacting residues (59 for distance-based contacts with BaSiLiCo versus 30 for explicit hydrogen-bond/hydrophobic interactions with PLIF), main hot spots discussed above (residue alignment positions 545, 557, 1361, 1362, 1119, 1124, 1130, 1473, 1612, 1619) are equally identified by both approaches (Figs. 2a and 4).

#### Retrospective validation

**Fig. 5:**
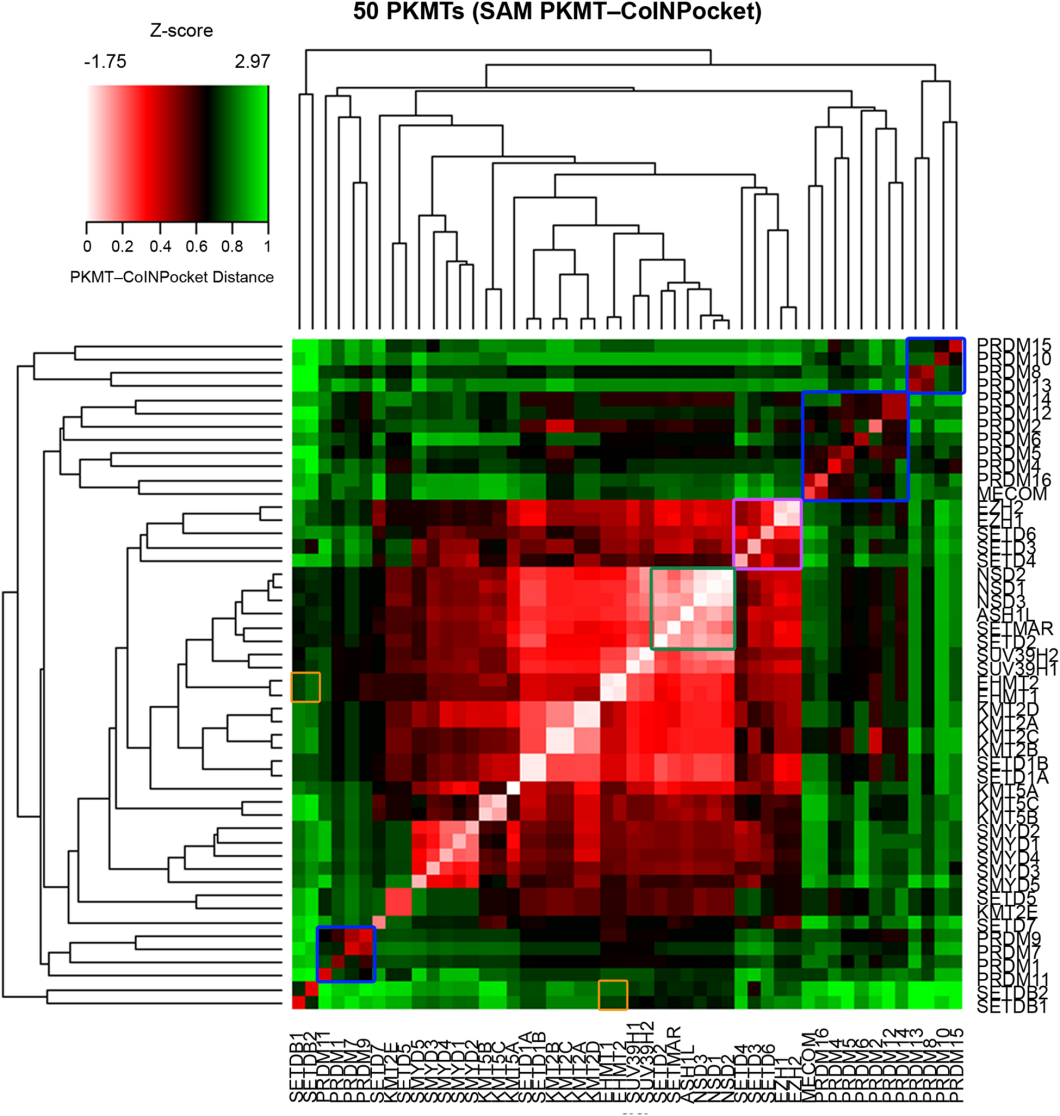
Heat map representation of 50 SET PKMTs based on PKMT-CoINPocket similarity for the SAM binding site. Targets discussed in the text are highlighted

**Fig. 6:**
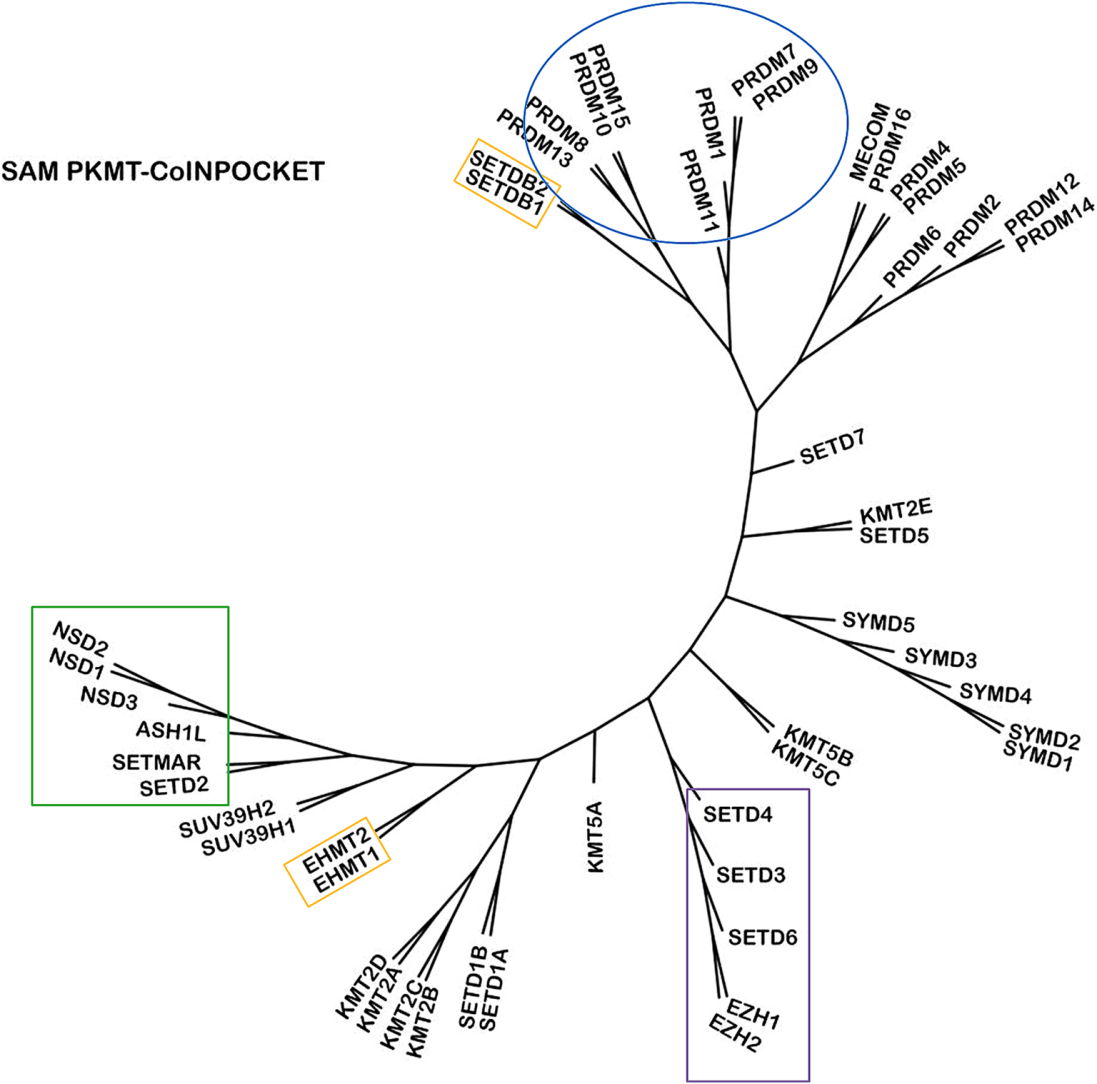
PKMT-CoINPocket organization of 50 PKMTs for the SAM-binding site. Targets discussed in the text are highlighted

Most of the SET subfamilies were grouped together in the PKMT-CoINPocket similarity heat map considering SAM interactions (Fig. 5 and derived pharmacological tree in Fig. 6), with a few surprising differences such as the partition of the PRDM subfamily into three different clusters or the higher remote cluster for SETDB1/SETDB2, far away from their sequence-based neighbors EHMT1/EHMT2 (Figs. 6 versus 1). Other distant sequence-based relationships, as for example between EZH1/EZH2 and SETD3/SETD4/SETD6 emerged by applying PKMT-CoINPocket comparisons (Fig. 6). It remains to experimentally validate this pharmacological relationship for any of the EZH2 inhibitors in Additional file 1: Tables S2 or S5 (e.g. for UNC1999 or GSK343, chemical structures in Fig. 8) as, to our knowledge, assays against any of these last three targets are not currently outsourceable. Other sequence-based relationships such as the one between SETD2 and NSDs were also captured by PKMT-CoINPocket score: SETD2 > NSD3 (0.90) > SETMAR (0.88) > NSD1 (0.87) > NSD2 (0.86), what can be retrospectively acknowledged according to the inhibitory profile of nucleoside-analogue inhibitors of SETD2 in Additional file 1: Table S2 (5LSS, 5LSX, 5LSY, 5LSZ, 5LT6 and 5LT7).

### Substrate binding site

#### Analysis of bound ligand interactions

**Fig. 7:**
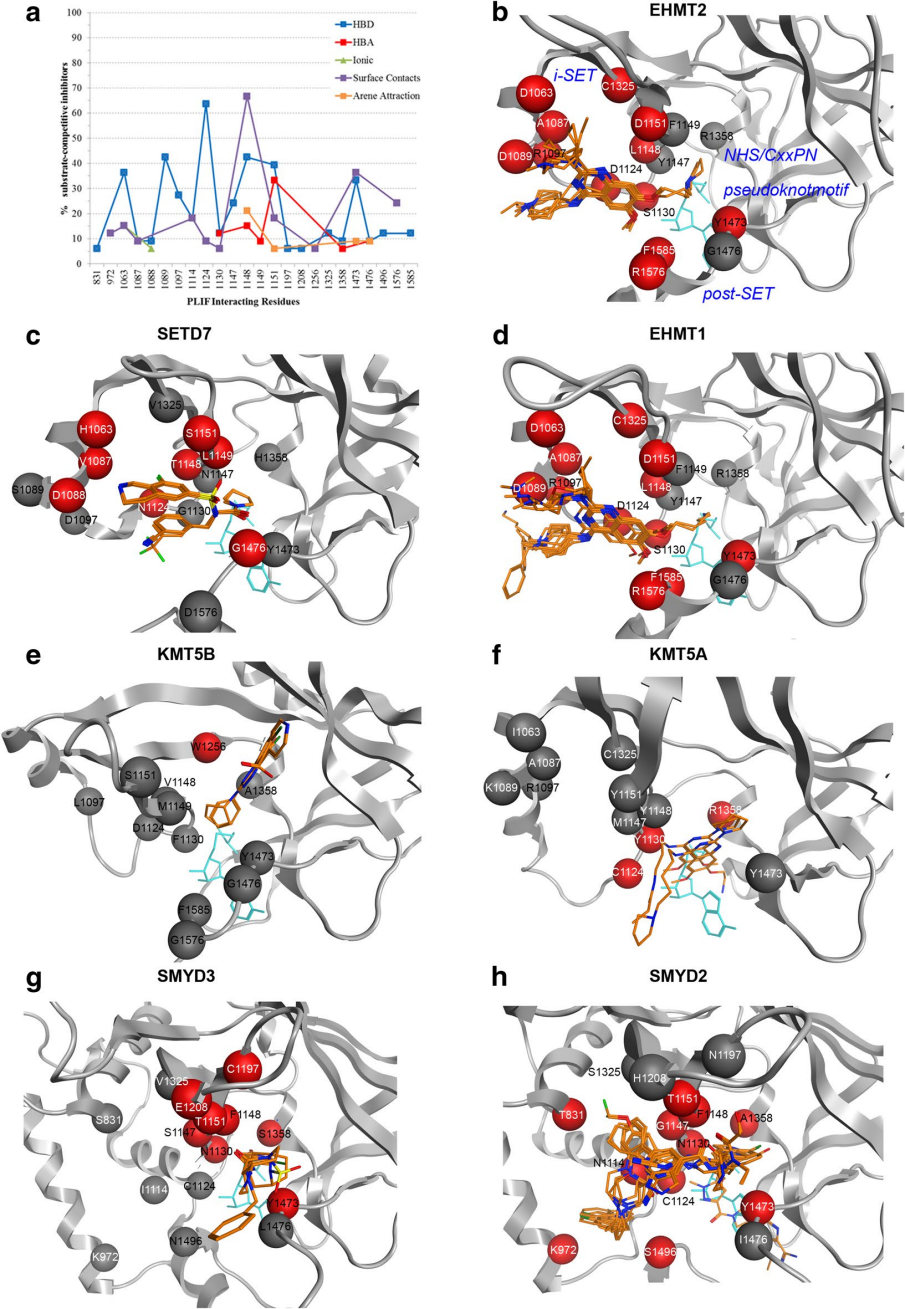
**a** Percentage of substrate-competitive inhibitors that interact with a given PLIF interacting residue according to interaction type. **b–h** Mapping of PLIF interacting residues for each of the seven PKMTs. Red balls correspond to residues for which at least one interaction was detected for all ligands (in orange) of this protein and grey balls to any of the remaining 24 different PLIF interacting residues (if conserved for the given PKMT at the corresponding positioning of the alignment) and for which no interaction was detected for any of the ligands of this PKMT. Note that because of structural protein differences in the alignment not all PLIF interacting residues could be mapped onto each protein. For reference, SAH is shown in blue

Compared to the SAM binding site, the number of PLIF detected interactions between the 33 substrate-competitive inhibitors and the 7 PKMTs is much reduced at the substrate binding site, with a total of 265 residue-based contacts, distributed across a total of 24 PLIF interacting residues (Fig. 7a. and Additional file 1: Table S7).

**Fig. 8:**
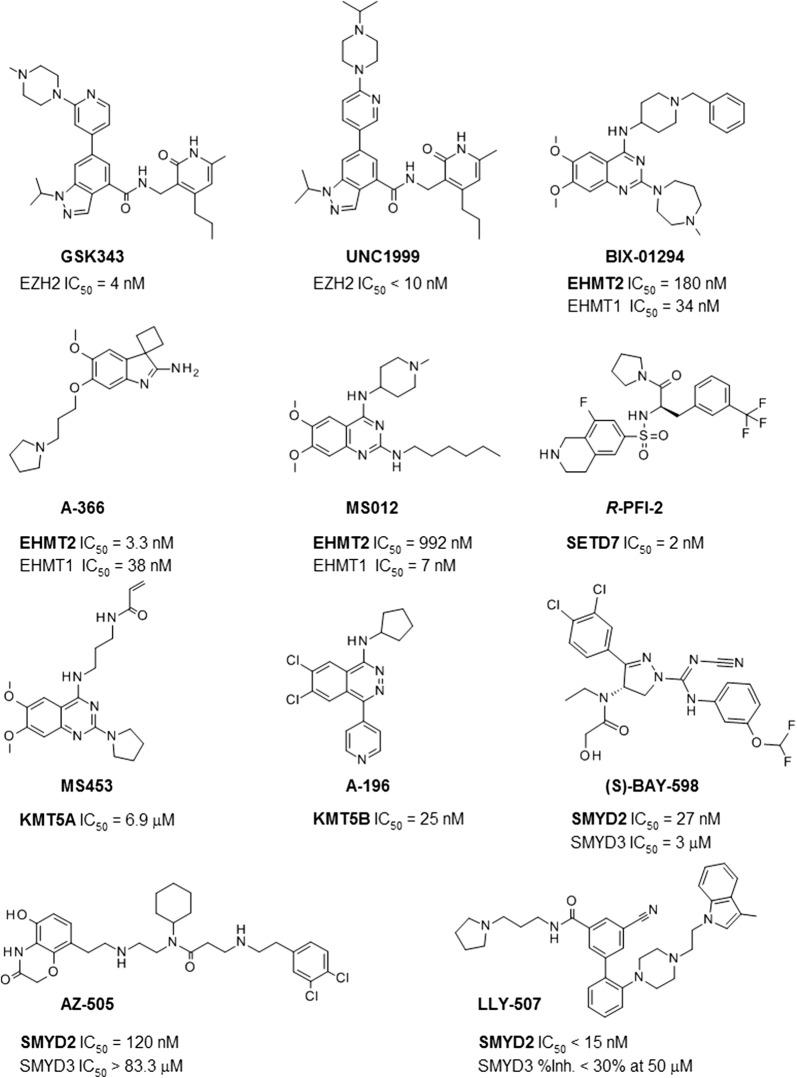
Chemical structures of selected PKMT inhibitors discussed in the text. Inhibitory activity against their primary target(s) (in bold if co-crystallized with)

As for the SAM-binding cavity, a preference for polar contacts over hydrophobic interactions is observed, highlighting the electronegative character of the substrate-binding groove [40]. Of note, there is a preference for the ligand acting as HBD instead of HBA: 53.6 versus 10.6%. Hydrophobic interactions, quantified as the number of surface contacts and arene attraction sum up a total of 33.2% of the interactions (27.5 and 5.7%, respectively) and explicit ionic interactions contribute to only 2.6% of the contacts. Next, we inspected the degree of conservation of PLIF interacting residues across the 7 PKMTs and which amino acids contribute to specific contacts. Figure 7b–h show the position of each PLIF interacting residue for each PKMT, with all its substrate-competitive inhibitors superposed (2D schematic representations for each separate inhibitor are in Additional file 1: Figure S12). As the average RMSD among all crystals of a given PKMT is low (< 2.5 Å for Cα (mean of 0.9 Å) and < 2.7 Å (mean of 1.3 Å) for all atoms, Additional file 1: Table S8), a unique representative crystal structure per PKMT is shown in Fig. 7b–h. Chemical structures of representative inhibitors discussed below are given in Fig. 8, with their inhibitory activity against its primary target(s).

Refer to Additional file 1: Tables S2 and S5 for full selectivity profiling and PDB entries. Despite all ligands occupying the substrate lysine binding channel (< 4 Å to the catalytic tyrosine of the post-SET domain at 1473—following position alignment numbering, see Additional file 1: Table S4 for a translation to the corresponding PDB numbering), they largely differ in the occupation of the remaining cavity of the peptide binding groove formed by post-SET and i-SET domains, emphasizing opportunities for the design of selective substrate competitive PKMT inhibitors. Conserved contacts between EHMT2, EHMT1 and SETD7 are clearly apparent by visual inspection of Fig. 7b–d, despite chemical differences between EHMT2/EHMT1 inhibitors (all of them are quinazolines (e.g. BIX-01294 and MS012 in Fig. 8), with the exception of the spiro[cyclobutane-1,3’-indole]-2’-amine of PDB 4NVQ against EHMT2 (A-366) and the 1,2,3,4-tetrahydroisoquinoline-6-sulfonamide-based SETD7 inhibitors such as *R*-PFI-2 in Fig. 8 (mean FCFP_4-similarity of 0.13 ± 0.02 between all EHMT2/EHMT1 inhibitors and SETD7 inhibitors, Additional file 1: Figure S13). Opposite, for the quinazoline compounds targeting KMT5A (e.g. MS453), which have a moderate chemical similarity with EHMT2/EHMT1 inhibitors (mean FCFP_4-similarity of 0.48 ± 0.10), an alternative binding mode to that observed in EHMT2/EHMT1 was found (Fig. 7f) [41], in which the inhibitors enter deeper into the lysine binding channel and most contacts with the i-SET domain are lost. For the phthalazine and 1H-pyrano[2,3-c]pyrazol-6-one KMT5B inhibitors (e.g. A-196), only surface contacts at residue at position 1256 (Trp264) are detected according to PLIF consensus (Fig. 7e). Besides interacting with the i-SET domain, SMYD2 and SMYD3 inhibitors occupy an additional cavity proximal to the α-helices at positions 831 (Thr105 and Ser101 for SMYD2 and SMYD3, respectively) and 972 (Lys145 and Lys140) of the alignment (Fig. 7g, h). Considering the range of alternative orientations of the ligands, only a few PLIF interacting residues are common to several PKMTs (Fig. 7) so as to stand out as hot spots. One of them is residue at position 1124, at the loop connecting the helix and the β-sheets (EHMT2, EHMT1, KMT5A and SETD7) and the α-helix of the i-SET domain of SMYD2. Despite non-sequence conservation (Asp for EHMT2/EHMT1, Asn for SETD7, Cys for KMT5A/SMYD2, Additional file 1: Table S4) and different secondary structure (e.g. SMYD2), it appears as a hot spot at the entrance of the lysine binding channel. A close residue at position 1130 also establishes contacts with different PKMT inhibitors: HBD, surface and HBA interactions with EHMT2, EHMT1, KMT5A, SMYD2 and SMYD3, respectively. Interestingly, this residue hot spot was also detected by Nguyen et al. when docking a library of fragments against six SET domain-containing PKMTs and its carbonyl group plays a role in increasing the nucleophilicity of SAM’s departing methyl group during catalysis [42]. The non-sequence conserved residue at position 1148 (L, T, Y, V or F) facing the catalytic Lys at 1473 and establishing hydrogen bond interactions with the substrate [5, 40], is also hydrogen-bonded to different ligands of EHMT2/EHMT1/SETD7 (HBD, 42%) and SMYD2/SMYD3 (HBA, 15%), as well as by hydrophobic interactions (67%, for the five PKMTs mentioned above). Position 1151, at one β sheet of the i-SET domain, is another exploited residue by inhibitors of EHMT2/EHMT1/SMYD2 (via HBD) and SETD7/SMYD3/SMYD2 (via HBA). Finally, the catalytic Tyr at position 1473 is HBD targeted by EHMT2, SMYD3 and SMYD2. When clustering these 33 complexes based on PLIF, the high distances among different PKMTs correlate with the lack of binding mode agreement visually observed (Additional file 1: Figure S14).

#### Prediction of pharmacological similarities with PKMT-CoINPocket Model

**Fig. 9:**
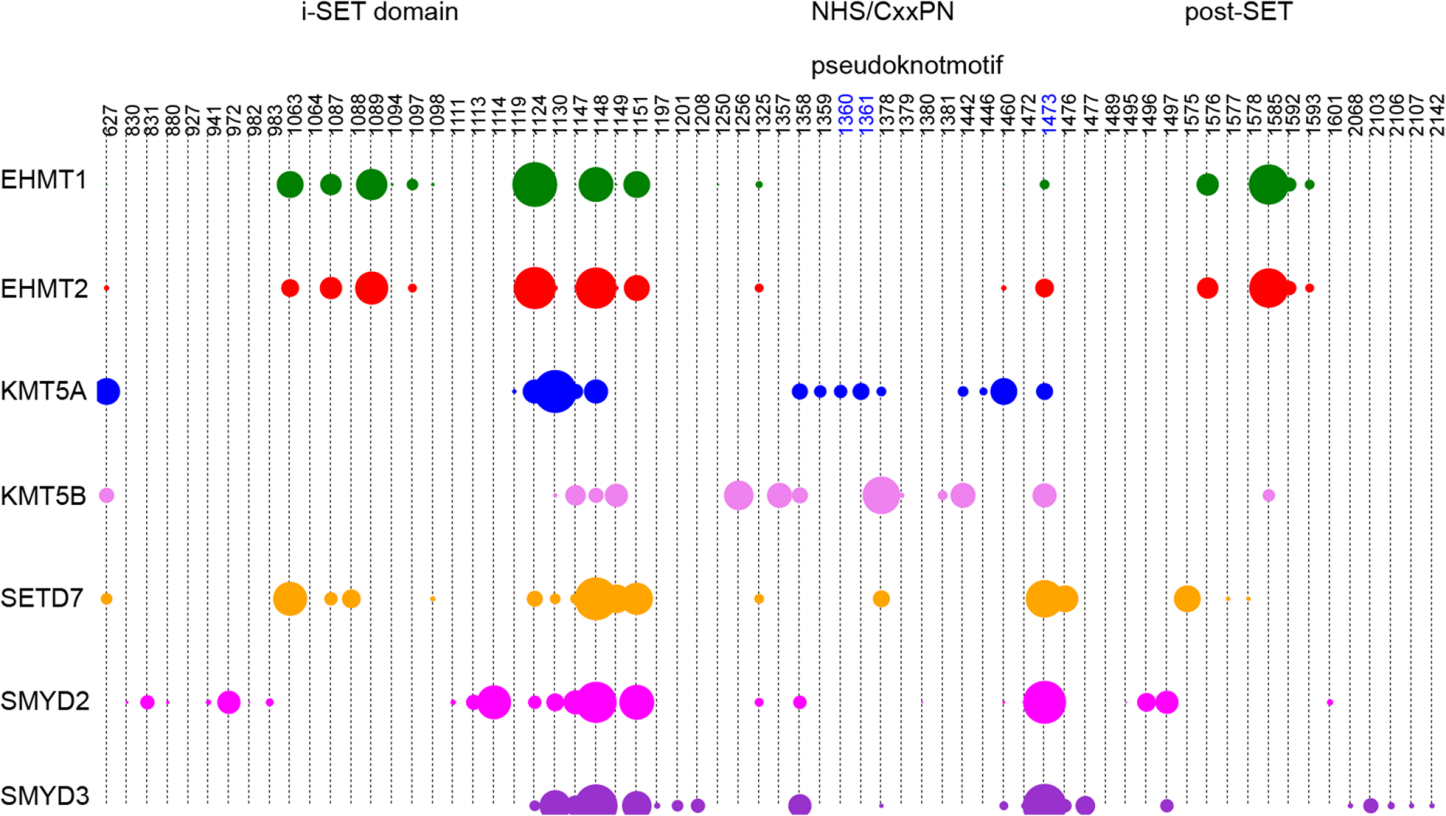
BaSiLiCO ligand contact map of substrate-competitive inhibitors of 7 PKMTs. The area of the circles reflects the relative strength of the ligand contact and residue position in the alignment

Here, the calculation of PKMT—substrate-competitive ligand BaSiLiCo contact strengths resulted in the identification of a ‘cloud’ of 75 residue positions that outline the i-SET, NHS/CxxPN pseudoknotmotif and the post-SET domain (Fig. 9). Again, this cloud of 75 interacting residues detected by BaSiLiCo is much higher than the 24 PLIF interacting residues because of differences in contact definitions, although, again, main hot spots identified by both approaches and discussed above are mostly in common (e.g. 1124, 1130, 1148, 1151 and 1473).

**Fig. 10:**
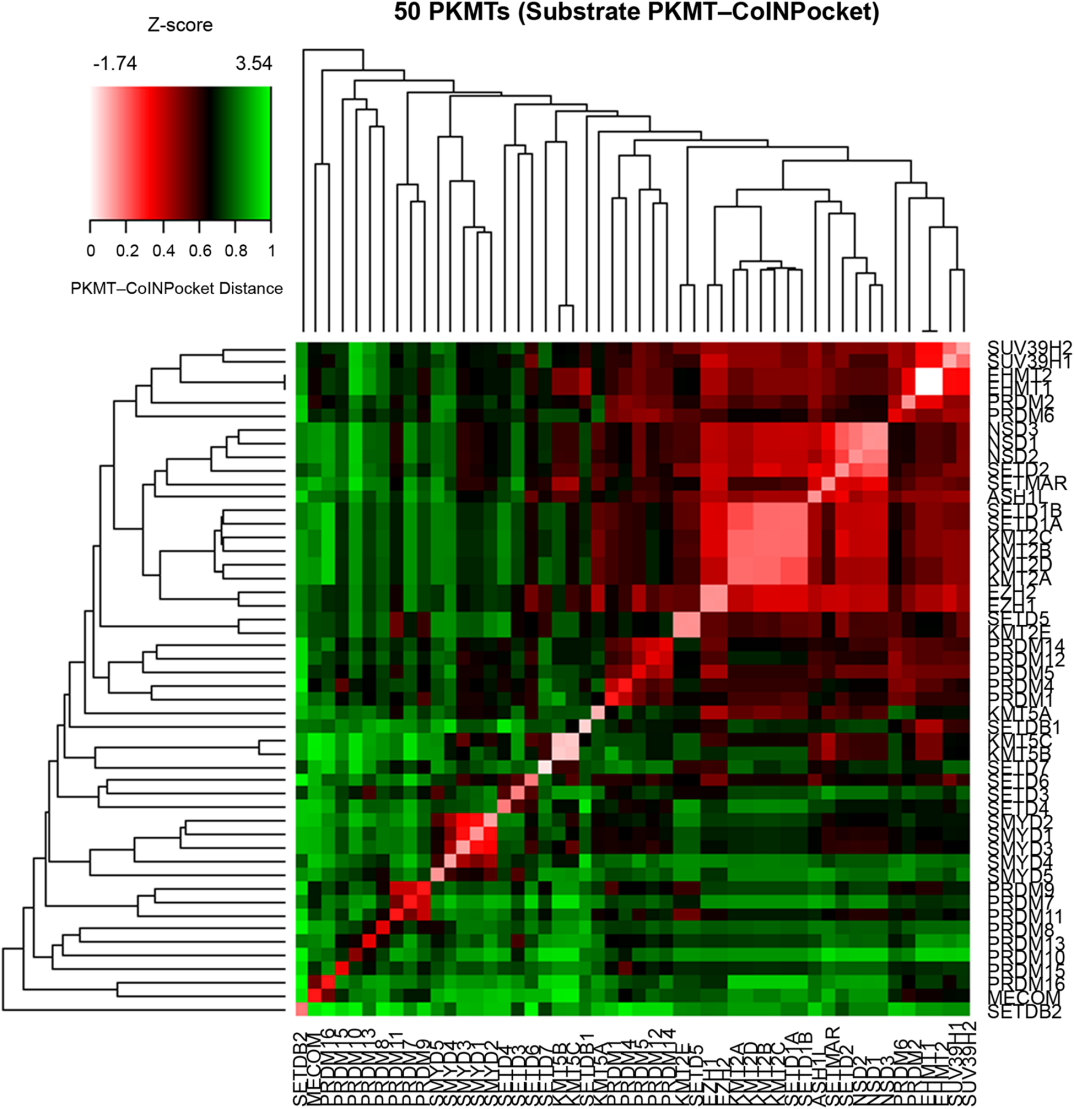
Heat map representation of 50 SET PKMTs based on PKMT-CoINPocket similarity for the substrate binding site

**Fig. 11:**
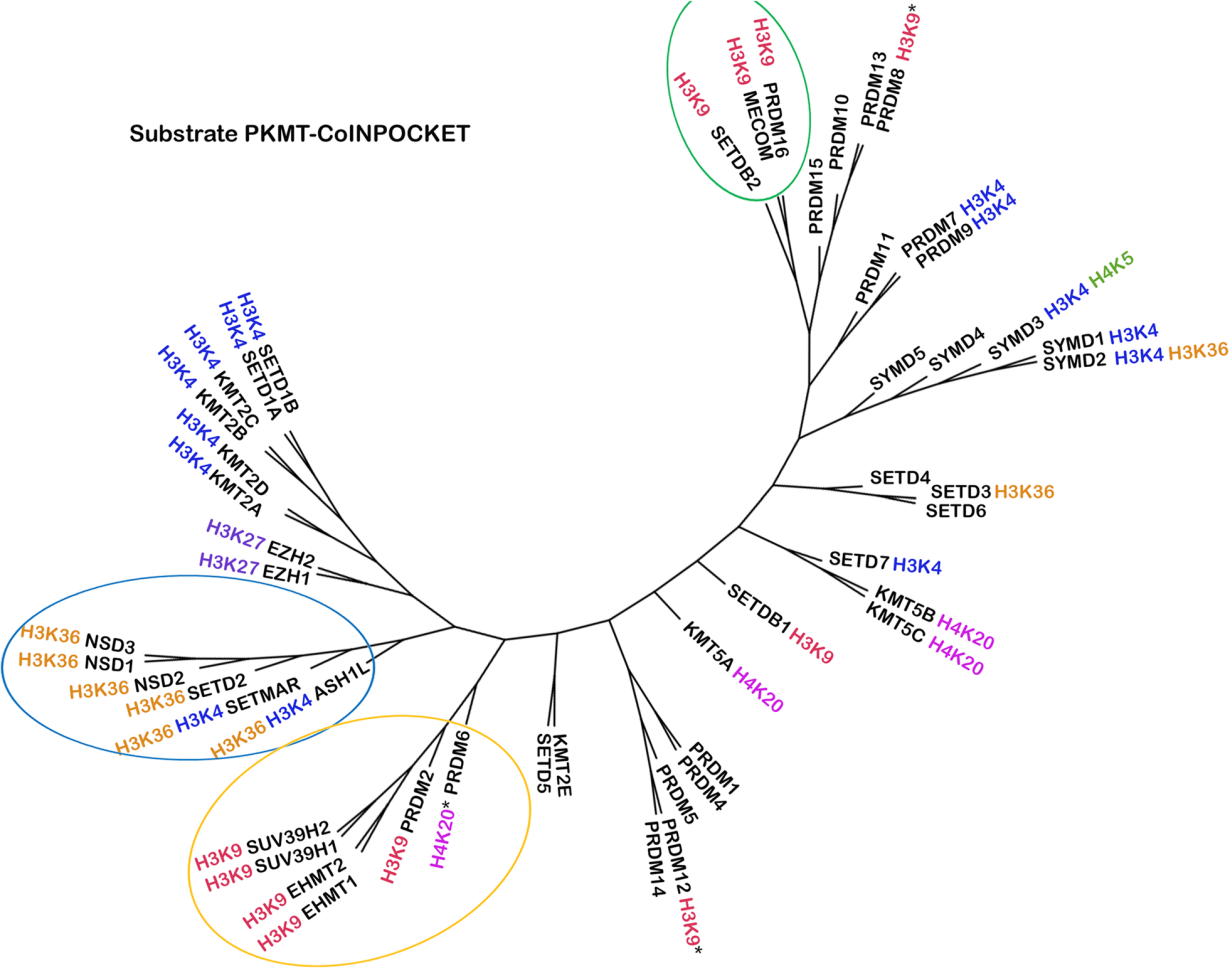
PKMT-CoINPocket organization of 50 PKMTs for the substrate binding site. Known methylation sites for histone H3 and H4 tails are labeled [3] (*indicates that this information was directly taken from UniProt). Targets discussed in the text are highlighted

As for the SAM binding site, PKMT-CoINPocket similarity heat map (Fig. 10) and its derived tree (Fig. 11) arrange some SET-domain containing PKMTs outside of their subfamily, while most relationships are conserved (e.g. for the SMYD subfamily). For example, PRDM2 and PRDM6 shift away from the other PRDMs and toward members of the Suvar3-9 subfamily (SUV39H2, SUV39H1, EHMT2 and EHMT1). Interestingly, all of them (except for PRDM6), share H3K9 as histone target. SETMAR groups with members of the SETD2 subfamily (SETD2, NSDs and ASH1L), all of them having in common H3K36 as substrate. Analogously, for the case of SETDB2 regrouped with H3K9-targeting PRDMs MECOM and PRDM16. This does not mean that PKMT-CoINPocket yields PKMT arrangement according to the histone substrate (Fig. 11), but simply suggests that the new organization has sense from the view point of common substrates. Compared to the SAM binding site, the greater Z-score of the substrate binding site (3.54 against 2.97, respectively in Figs. 5 and 10) emphasizes the greater diversity of this last pocket (as also observed when comparing the similarity matrix of both sites, Additional file 1: Figures S15 and S16 for the co-factor and substrate sites, respectively).

#### Retrospective validation

In order to assess whether the PKMT-CoINPocket score reflects pharmacological relationships, the selectivity profile of substrate-competitive PKMT inhibitors in Additional file 1: Tables S2 and S5 was examined. Full profiling of most of these inhibitors is not publicly available and, if available, most of them are highly selective, targeting only one or at maximum two closely sequence related PKMTs in the low nanomolar range (e.g. EHMT1 and EHMT2 or SMYD2 and SMYD3) and a few other targets in the low micromolar range (at maximum). Moreover, there are pairs of proteins for which truly selective inhibitors are available (inactive against one of the targets at high micromolar range) but that also share inhibitors able to bind to both proteins (despite selectivity higher than 3 log units). For example, among all low-nanomolar SMYD2 inhibitors in Fig. 8 and Additional file 1: Table S2, A-893 and (S)-BAY-598 bind to SMYD3 (at least in the low micromolar range) while others are highly selective for SMYD2 over SMYD3 (AZ-505, LLY-507, with IC_50_ against SMYD3 > 50 µM). With that in mind, we concentrated on experimentally detected non-selective profiles, understood as the ability of the ligand to bind even at low micromolar range. Leaving aside obvious relationships, some interesting predicted unsuspected neighbors in PKMT-CoINPocket organization such as EHMT1/EHMT2 and NSD2 (cluster in Fig. 10) correlate with the observed activities of BIX-01294 and A-366 against EHMT1/EHMT2 (low nanomolar) and NSD2 (micromolar). Another interesting pair, EHMT1/EHMT2 and SETD2 (with PKMT-CoINPocket similarity of 0.45) can be acknowledged by the activity of MS012 against both targets (EHMT1 IC_50_ = 7 nM and 40% inhibition of SETD2 at 10 µM). Finally, SETD7 and EZH2 have a PKMT-CoINPocket similarity of 0.41 (EZH2 ranked at position 5 for the most similar targets of SETD7 at the substrate binding site), in line with the activities of (*R*)-PFI-2 against SETD7 (IC_50_ = 2 nM) and EZH2 (~ 50% inhibition at 50 µM).

#### Prospective validation

**Fig. 12:**
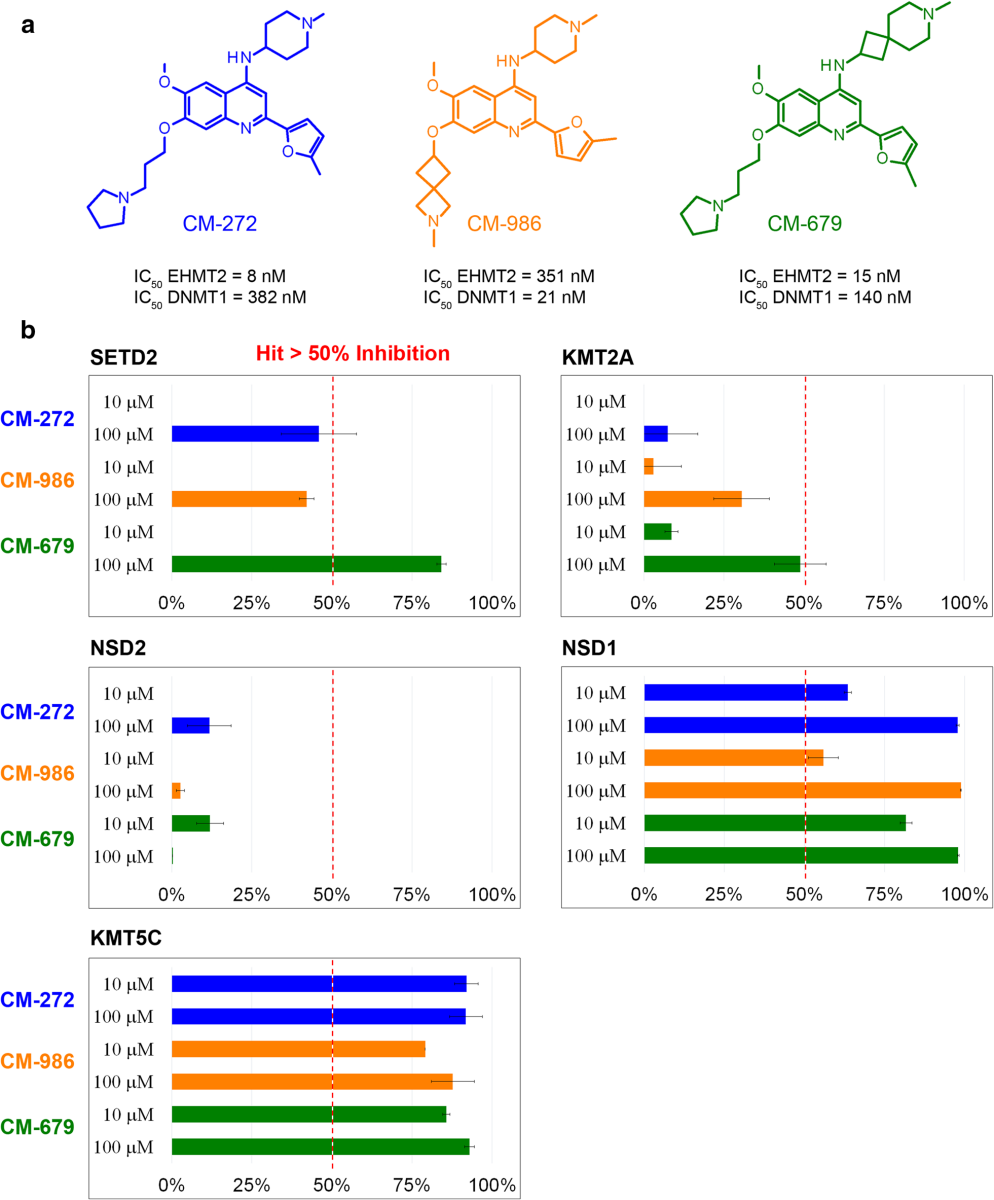
**a** Chemical structures and IC_50_ values against EHMT2 (G9a) and DNMT1 for proprietary compounds CM-272, CM-986 and CM-679. **b** Percentage of inhibition of compounds CM-272, CM-986 and CM-679 against selected targets similar to EHMT2

We discovered novel 4-aminoquinoline inhibitors targeting EHMT2 and DNMT1 at the low nanomolar range [36–39]. Because of their therapeutic relevance in cancer, we selected three of them (CM-272, CM-679 and CM-986, Fig. 12a) with diverse selectivity profiles against their primary targets to be profiled against those SET-domain containing PKMTs with high PKMT-CoINPocket similarity scores for EHMT2: SETD2, KMT5C, KMT2A, NSD1 and NSD2 (Fig. 12b and Additional file 1: Table S9). Unfortunately, assays for other PKMT-CoINPocket-similar targets to EHMT2 (PRDM2 and PRDM6) could not be outsourced and remain to be confirmed. For SETD2, the three compounds exhibited > 40% inhibition at 100 µM (initially, only CM-272 activity at 10 µM had been tested for SETD2, with negligible inhibition [36]). Particularly, CM-679 had the strongest effect (84% at 100 µM). Although this concentration is not therapeutically relevant, it provides a certain validation of the computational approach to find weak PKMT binders for closely pharmacologically related targets that might be useful as starting points for identifying potent chemical probes. For KMT5C and KMT2A, there is not a single pair in Additional file 1: Tables S2 and S5 supporting this selection, but only negative cases (e.g. MS0122, MS0124, UNC0224, A-366). For KMT2A, only CM-679 displays a weak inhibition of 48% at 100 µM. More interestingly, all three compounds bind at the low micromolar range to KMT5C. Although it is interesting because KMT5C substrate is H4K20 (different from the H3K9 substrate of EHMT2, the original target of these compounds); there are already nanomolar inhibitors available for KMT5C (e.g. A-196). This is not the case for NSD1, an oncoprotein overexpressed in numerous cancers such as acute myeloid leukemia [43] that remains orphan. All three compounds inhibit NSD1 higher than 50% at 10 µM and in a dose-response manner, especially CM-679. Interestingly, very recently it was shown that the EHMT2 inhibitor BIX-01294 does also inhibit NSD1 (IC_50_ = 112 µM) [44]. Taken altogether, apart from the validation of the PKMT-CoINPocket approach, these results open the door to the repurposing of EHMT2 inhibitors towards NSD1, at least to serve as starting points for potency optimization.

## Conclusions

A detailed update of experimentally detected ligand interactions with SET domain containing PKMTs at the SAM and substrate binding sites is discussed. For the SAM binding site, interactions of the N1 and N3 of the adenine ring and hydroxyl groups of the ribose of SAM/SAH evidence a higher similarity with PRMTs that initially expected [19]. This analysis also reveals interaction patterns that are conserved across different subfamilies and that could be exploited to develop selective SAM competitive inhibitors. While the analysis of substrate-bound inhibitors is restricted to only 7 PKMTs with ligands occupying different sites of the cavity, some important hot spots arise that are shared by different series of inhibitors. To our knowledge, this is the first study that applies protein ligand interaction fingerprints to the study of SET domain containing PKMTs. Interestingly PLIF approach captures changes in the interaction network of SAM/SAH molecules with the different PKMTs, despite strong binding mode conservation, as evidenced by the low RMSD of superposition of the ligands. As the predictive power of this approach is restricted to proteins for which a crystal structure is available, the novel GPCR-CoINPocket methodology was adapted for the entire family of SET domain containing proteins. For both sites, the novel organization retains sequence-based relationships, although some interesting unexpected similarities appeared that were confirmed experimentally for a set of dual EHMT2 / DNMT1 inhibitors at the substrate binding site. It must be noted that during our initial selectivity profiling of CM-272 against 8 closely related PKMTs, only EHMT1 was identified [36]. Here, KMT5C and the orphan NSD1 were identified as alternative targets for this chemical series. This is especially relevant for NSD1, as it requires deorphanization and these compounds could be used as starting points to develop chemical probes with enhanced potency and selectivity. For the retrospective study cases, it should be noted that because of the lack of massive ligand information, a comprehensive benchmarking study is still unaffordable. Three are the main disadvantages of this approach: it is very sensible to the alignment, water contacts were not considered and the presence of many inserts (gaps) in the alignment avoids translation of contact information to all the proteins. Moreover, in the current analysis the flexibility of the proteins was not contemplated. In this sense, the incorporation of contact occupancies (and alternative explored contacts) resulting from the analysis of molecular dynamics trajectories could improve the approach. Finally, we think that this study provides a useful compilation of available selectivity data for known inhibitors of PKMTs at both sites.

## Additional files

**Additional file 1.** Additional figures and tables.

**Additional file 2.** Sequence alignment.

## Abbreviations

BaSiLiCo: binding site ligand contacts
FCFP_4: functional class fingerprint
GPCR-CoINPocket: GPCR contact-informed neighboring pocket
HB: hydrogen bonds
HBD: hydrogen bond donor
HBA: hydrogen bond acceptor
MT: methyltransferase
PDB: protein data bank
PKMT: protein lysine methyltransferase
PKMT-CoINPocket: PKMT contact-informed neighboring pocket
PLIF: protein–ligand-interaction fingerprint (MOE implementation)
PRMT: protein arginine methyltransferase
RMSD: root-mean-square deviation
SAH: S-adenosyl-L-homocysteine
SAM: S-adenosyl-l-methionine
SVL: scientific vector language
UPGMA: unweighted pair group method with arithmetic mean
