# Supplementary material for: Novel pharmacological maps of protein lysine methyltransferases: key for target deorphanization
Source: J Cheminform. 2018 Jul 21;10:32. doi: 10.1186/s13321-018-0288-5 (PMC6054832; doi:10.1186/s13321-018-0288-5)
Supplement: Supplementary file 1 — Additional file 1. Additional figures and tables. [file 13321_2018_288_MOESM1_ESM.docx]

**Novel Pharmacological Maps of Protein Lysine MethylTransferases: Key for Target Deorphanization.**

Obdulia Rabal, Andrea Castellar and Julen Oyarzabal

Small Molecule Discovery Platform. Molecular Therapeutics Program, Center for Applied Medical Research, CIMA, University of Navarra, Pamplona, Spain.

A.C ([acastellar@alumni.unav.es](mailto:acastellar@alumni.unav.es)).

Correspondence should be addressed to O.R. ([orabal@unav.es](mailto:orabal@unav.es)) and J.O. ([julenoyarzabal@unav.es](mailto:julenoyarzabal@unav.es)), Pio XII, 55, 31008 Pamplona, Spain, Tel: +34 948 19 47 00 Ext 2000 and 2044, Fax: +34 948 19 47 15.

# Table of Contents

**S1**. **Table S1**. List of 50 SET domain-containing PKMTs and 104 crystal structures in this study………………………………………………………………………………………...........3

**S2**. **Table S2**. SAM- and substrate competitive inhibitors in crystal structures..…………………………………………………………………………………..........8

**S3**. **Figure S1**. Structural superimposition of 104 PKMT crystals with a full-sequence alignment…………………………………………………………………………………..........20

**S4**. **Figure S2**. Structural superimposition of 104 PKMT crystals with MT alignment (cofactor view)………………………………………………………………………………….................21

**S5. Figure S3.** Overlay of the 23 PKMT structures with MT alignment………………………22

**S6.** **Figure S4**. Pairwise matrix of RMSD values of superposition of the 101 complexes with cofactor (SAM/SAH) and SAM-competitive inhibitors with 23 PKMTs………………….....23

**S7**. **Table S3**. Translation of sequence alignment positions to PDB residue number for each the 10 relevant PKMTs with cofactor bound structures discussed in the text……………………...24

**S8. Table S4.** Translation of sequence alignment positions to PDB residue number for each of the 7 PKMTs with substrate-competitive inhibitors discussed in the text……………………...25

**S9. Table S5.** Chemical structure, biochemical inhibitory activity against primary target of cofactor and substrate-competitive inhibitors of PKMTs lacking a crystal structure…………..25

**S10. Table S6**. Distribution of PLIF detected contacts for the 101 complexes of SET domain-containing PKMTs bound with cofactor (SAM / SAH) or SAM-competitive inhibitors………………………………………………………………………………………..28

**S11. Figure S5.** Mapping of PLIF interacting residues for 6 complexes of representative PKMTs complexed with either SAM or SAH…………………………………………………32

**S12. Figure S6**. 2D schematic view of PLIF conserved contacts across the 101 complexes of the cofactor (SAM or SAH) and SAM-competitive inhibitors against 23 different PKMTs……...33

**S13. Figure S7.** Heat map of the RMSD of superposition between the 41 SAM molecules….38

**S14. Figure S8.** Heat map representation of PLIF similarities for the 41 PKMT-SAM complexes……………………………………………………………………………………….39

**S15. Figure S9.** Heat map of the RMSD of superposition between the 41 SAH molecules….40

**S16. Figure S10.** Heat map representation of PLIF similarities for the 41 PKMT-SAH complexes………………………………………………………………………………………41

**S17. Figure S11.** Heat map representation of PLIF similarities for the 101 SAM-binding PKMT complexes………………………………………………………………………………………42

**S18. Table S7.** Distribution of PLIF detected contacts for the 33 complexes of SET domain-containing PKMTs bound with substrate-competitive inhibitors………………………………43

**S19. Figure S12.** 2D schematic view of PLIF conserved contacts across the 33 complexes of the substrate-competitive inhibitors against 7 different lysine methyltransferases………………...45

**S20. Table S8.** Average RMSD (Cα and All atoms) among all 7 PKMTs with substrate competitive inhibitors…………………………………………………………………………...46

**S21. Figure S13.** FCFP_4-based similarities between the 30 substrate-competitive inhibitors of the PKMTs…………………………...…………………………………………………………47

**S22.** **Figure S14.** Heat map representation of PLIF similarities (expressed as distances) for the 33 substrate-competitive complexes…….………………………………………………….…48

**S23. Figure S15.** Heat map representation of Gonnet similarity matrix (expressed as distances) for the SAM-binding site…………………..………………………………………….……...49

**S24. Figure S16.** Heat map representation of Gonnet similarity matrix (expressed as distances) for the substrate-binding site…………………………………………..……………………..50

**S25. Table S9.** Percentage of inhibition for the proprietary compounds………………………51

**References**……………………………………………………………………………………...52

**Table S1**. List of 50 SET domain-containing PKMTs. Crystal structures having substrate or substrate-competitive inhibitors together with cofactor (SAM/SAH) are listed within the “PDB entries with substrate binding molecules” column.

| PKMT Name  (Hugo Nomenclature) | Alternative common names | Human Protein  UniProt Entry | PDB entries with cofactor binding molecules | | | PDB entries with substrate binding molecules | | Total # of PDBs |
| --- | --- | --- | --- | --- | --- | --- | --- | --- |
|  |  |  | SAM | SAH | Inhibitors | Small Molecules | Peptides or Substrate |  |
| EHMT2 | G9a / KMT1C | Q96KQ7 |  | 2O8J |  | 3K5K (SAH)  3RJW (SAH)  4NVQ (SAH)  5TTF (SAM)  5TUY (SAM)  5VSC (SAM)  5VSE (SAM) | 5JIY (SAM) | 9 |
| EHMT1 | GLP / KMT1D | Q9H9B1 |  | 2IGQ | 3SW9 (SFG; peptide) | 3FPD (SAH)  3MO0 (SAH)  3MO2 (SAH)  3MO5 (SAH)  5TTG (SAM)  5TUZ (SAM)  5VSD (SAM)  5VSF (SAM) | 2RFI (SAH)  3HNA (SAH)  3SWC (SAH) | 13 |
| SETDB1 | KMT1E | Q15047 |  |  |  |  |  | 0 |
| SETDB2 | KMT1F | Q96T68 |  |  |  |  |  | 0 |
| SUV39H2 | KMT1B | Q9H5I1 | 2R3A |  |  |  |  | 1 |
| SUV39H1 | KMT1A | O43463 |  |  |  |  |  | 0 |
| SETMAR |  | Q53H47 |  | 3BO5 |  |  |  | 1 |
| SETD5 |  | Q9C0A6 |  |  |  |  |  | 0 |
| KMT2A | MLL / MLL1 | Q03164 |  | 5F6L |  |  | 2W5Z (SAH) | 2 |
| KMT2B | MLL4 | Q9UMN6 |  |  |  |  |  | 0 |
| KMT2C | MLL3 | Q8NEZ4 |  | 5F59 |  |  |  | 1 |
| KMT2D | MLL2 | O14686 |  | 4Z4P |  |  |  | 1 |
| KMT2E | MLL5 | Q8IZD2 |  |  |  |  |  | 0 |
| SETD1A | KMT2F | O15047 |  |  |  |  |  | 0 |
| SETD1B | KMT2G | Q9UPS6 |  |  |  |  |  | 0 |
| EZH2 | KMT6A | Q15910 |  |  | 5IJ7^(2)^  5LS6 |  | 5HYN (SAH) | 3 |
| EZH1 | KMT6B | Q92800 |  |  |  |  |  | 0 |
| KMT5A^(1)^ | SETD8 | Q9NQR1 | 4IJ8 |  |  | 5T5G  5TH7 | 1ZKK (SAH)  2BQZ (SAH)  5TEG (SAM) | 7^(1)^ |
| SETD7 | SET7/9 | Q8WTS6 | 1N6C | 1MT6 | 3CBP (SFG; peptide)  3VUZ  3VV0 | 4E47 (SAM)  4JDS (SAM)  4JLG (SAM) | 1O9S (SAH)  1XQH (SAH)  2F69 (SAH)  3CBM (SAH)  3M53 (SAH)  3OS5 (SAH)  4J8O (SAH)  4J83 (SAM) | 16 |
| SETD2 | KMT3A | Q9BYW2 |  | 4H12 | 5LT8 (SFG)  4FMU  5LSS  5LSX  5LSY  5LSZ  5LT6  5LT7 |  | 5V21 (SAM)  5V22 (SAH) | 11 |
| ASH1L | KMT2H | Q9NR48 | 4YNM |  |  |  |  | 1 |
| NSD1 | KMT3B | Q96L73 | 3OOI |  |  |  |  | 1 |
| NSD2 | WHSC1  MMSET  KMT3G | O96028 | 5LSU |  |  |  |  | 1 |
| NSD3 | WHSC1L1  KMT3F | Q9BZ95 | 5UPD |  |  |  |  | 1 |
| SETD6 |  | Q8TBK2 |  |  |  |  | 3QXY (SAM)  3RC0 (SAM) | 2 |
| SETD4 |  | Q9NVD3 |  |  |  |  |  | 0 |
| SETD3 |  | Q86TU7 | 3SMT |  |  |  |  | 1 |
| SMYD3 | KMT3E | Q9H7B4 | 3QWP | 3OXF  3OXG | 3PDN (SFG)  5HI7 | 5CCL (SAM)  5CCM (SAM) | 5EX0 (SAH)  5EX3 (SAH)  5HQ8 (SAH) | 10 |
| SMYD2 | KMT3C | Q9NRG4 | 3TG4 | 3QWV^(3)^ | 3QWW (SFG)^(3)^  5WCG^(4)^ | 3S7B (SAM)  4YND (SAM)  5ARF (SAM)  5ARG (SAM)  5KJK (SAM)  5KJM (SAM)  5KJN (SAM)  4WUY (SAH) | 3S7D (SAH)  3TG5 (SAH)  3S7F (SAM) | 15 |
| SMYD1 | KMT3D | Q8NB12 |  |  | 3N71 (SFG)^(3)^ |  |  | 1 |
| SMYD5 |  | Q6GMV2 |  |  |  |  |  | 0 |
| SMYD4 |  | Q8IYR2 |  |  |  |  |  | 0 |
| KMT5B | SUV420H1 | Q4FZB7 | 3S8P |  |  | 5CPR (SAM)  5WBV (SAM) |  | 3 |
| KMT5C | SUV420H2 | Q86Y97 | 3RQ4 |  |  |  | 4AU7 (SAH)^(3)^ | 2 |
| PRDM1 |  | O75626 |  |  |  |  |  | 0 |
| PRDM2 |  | Q13029 |  |  |  |  |  | 0 |
| MECOM | PRDM3  MDS1 | Q13465 |  |  |  |  |  | 0 |
| PRDM4 |  | Q9UKN5 |  |  |  |  |  | 0 |
| PRDM5 |  | Q9NQX1 |  |  |  |  |  | 0 |
| PRDM6 | KMT8C | Q9NQX0 |  |  |  |  |  | 0 |
| PRDM7 |  | Q9NQW5 |  |  |  |  |  | 0 |
| PRDM8 | KMT8D | Q9NQV8 |  |  |  |  |  | 0 |
| PRDM9 | KMT8B | Q9NQV7 |  |  |  |  | 4C1Q (SAH)^(3)^ | 1 |
| PRDM10 |  | Q9NQV6 |  |  |  |  |  | 0 |
| PRDM11 |  | Q9NQV5 |  |  |  |  |  | 0 |
| PRDM12 |  | Q9H4Q4 |  |  |  |  |  | 0 |
| PRDM13 |  | Q9H4Q3 |  |  |  |  |  | 0 |
| PRDM14 |  | Q9GZV8 |  |  |  |  |  | 0 |
| PRDM15 |  | P57071 |  |  |  |  |  | 0 |
| PRDM16 | KMT8F | Q9HAZ2 |  |  |  |  |  | 0 |
| **TOTAL** |  |  | **12** | **11** | **19** | **32** | **29** | **104** |

(1) For KMT5A, PDB entry 5W1Y has an allosteric ligand, not listed in this table and was discarded from further analysis.

(2) Homo sapiens Anolis carolinensis (95% sequence identity with human); (3) Mus Musculus, with sequence identities in the MT domain with human of 92.2% (SMYD2), 97.6% (SMYD1), 94.8% (KMT5C) and 86.8% (PRDM9); (4) Bisubstrate inhibitor (SAM-competitive and substrate competitive).

**Table S2.** Chemical structure, biochemical inhibitory activity against primary target and selectivity of the SAM- and substrate-competitive inhibitors: 13 SAM-competitive, 1 bisubstrate and 29 substrate-competitive.

| Chemical Structure | PDB  entry | Ligand  Name | Primary Target (crystal) | Selectivity | Binding  Site |
| --- | --- | --- | --- | --- | --- |
|  | 5IJ7[1] |  | EZH2 (PCR2) HsPRC2_4  K_i_ = 1.63nM[1] |  | SAM |
|  | 5LS6[2] |  | EZH2  IC_50_ < 10nM[2] |  | SAM |
|  | 3SW9[3]  3CBP[4]  5LT8[5]  3PDN[6]  3QWW[7]  3N71[8] | Sinefungin (SFG) | EHMT1  IC_50_ = 513µM  SETD7  IC_50_ = 1µM  SETD2  71% at 30µM[5]  SMYD3  SMYD2  SMYD1 | CARM1[9]  IC_50_ = 0.4µM  PRMT1[9]  IC_50_ = 1µM  PNMT1[9]  IC_50_ = 3.7µM  SUV39H2[9]  IC_50_ = 4.6µM  IC_50_ > 10 µM for:[9]  KMT2A, EHMT2,  PRMT3, EZH2,  DOT1L, KMT5B KMT5C, KMT5A | SAM |
|  | 3VUZ[10] | AAM-1 | SETD7  ~10% at 10µM |  | SAM |
|  | 3VV0[10] | DAAM-3 | SETD7  ~50% at 10µM |  | SAM |
|  | 4FMU[9] |  | SETD2  IC_50_=0.8µM | CARM1  IC_50_ = 2.96µM  SETD7  IC_50_ = 2.2µM  PRMT1  IC_50_ = 9.5µM  EZH2  IC_50_ = 24µM  KMT2A  IC_50_ = 14µM  SUV39H2  IC_50_ = 10µM  SETD1  IC_50_ = 0.8µM  KMT5B  IC_50_ > 50µM  KMT5C  IC_50_ > 50µM  PRMT3  IC_50_ ~100 µM  KMT5A  IC_50_ > 100µM  EHMT1  IC_50_ > 100µM  EHMT2  IC_50_ > 100µM | SAM |
|  | 5LSS[5] |  | SETD2  IC_50_=0.49µM | NSD2  IC_50_ = 3.3µM | SAM |
|  | 5LSX[5] |  | SETD2  IC_50_=1.9µM | NSD2  52% at 1000µM | SAM |
|  | 5LSY[5] |  | SETD2  IC_50_ = 0.9µM | NSD2  IC_50_ = 1.8µM | SAM |
|  | 5LSZ[5] |  | SETD2  36% at 30µM | NSD2  IC_50_ = 69µM | SAM |
|  | 5LT6[5] |  | SETD2  IC_50_ = 3.9µM | NSD2  IC_50_ = 20µM | SAM |
|  | 5LT7[5] |  | SETD2  46% at 30µM | NSD2  48% at 300µM | SAM |
|  | 5HI7[11] | GSK2807 | SMYD3  IC_50_ = 13 µM | IC_50_ > 1000 µM for:  SMYD2, CARM1, PRMT1, DOT1L, ASH1L, PRMT5, EHMT2, NSD2, NSD3 | SAM |
|  | 5WCG | MTF003 | SMYD2 |  | Both |
|  | 3K5K[12] | UNC0224 | EHMT2  IC_50_ = 15nM[12] | EHMT1  IC_50_ = 20nM[12]  IC_50_ > 40000 nM for:  SETD7, KMT5A, PRMT3, JMJD2E[13] | Substrate |
|  | 3FPD[14] | BIX-01294 | EHMT2  IC_50_ = 180nM[15] | EHMT1  IC_50_ = 34nM[15]  NSD1  IC_50_ = 112µM[16]  NSD2  IC_50_ = 41µM[16]  NSD3  IC_50_ = 95µM[16]  SUV39H1(H320R)  0% < 10µM[17]  PRMT1  0% < 10µM[17]  SUV39H1  0% < 45µM[17]  SETD7  0% < 45µM[17] | Substrate |
|  | 3MO5[18] | E72 | EHMT1  IC_50_ = 136nM[18] | EHMT2 IC_50_=164nM[18]  SUV39H2  20% at 5 µM[18] | Substrate |
|  | 3MO0[18] | E11 | EHMT1  IC_50_ = 778nM[18] |  | Substrate |
|  | 3MO2[18] | E67 | EHMT1  IC_50_ = 273nM[18] |  | Substrate |
|  | 3RJW[15] | UNC0638 | EHMT2  IC_50_ <15 nM[15] | EHMT1  IC_50_ = 19nM[15]  JMJD2E  IC_50_= 4.5µM[15]  DNMT1  IC_50_=1287nM[19]  IC_50_ > 10000 nM for:[15]  SUV39H1  SUV39H2  SETD7  KMT2A  EZH2  KMT5A  PRMT1  PRMT3 | Substrate |
|  | 5TTG^(1)^ [20]  5TTF^(1)^ [20] | MS012 | EHMT1  IC_50_ = 7nM[20]  EHMT2  IC_50_ 992nM[20] | SETD2 ~40% at 10µM[20]  SMYD2 ~30% at 10µM[20]  CARM1 ~25% at 10µM[20]  PRMT6 ~25% at 10µM[20]  METTL3-14 57% at 10µM[20]  BCDIN3D ~30% at 10µM[20]  0% Inh. at 1µM for:[20]  SUV39H1, SUV39H2, SETDB1, SETD7, KMT5A, KMT5B, KMT5C, KMT2A, KMT2C, PRDM9, SMYD3, PRC2, PRMT1, PRMT3, PRMT5, PRMT7, PRMT8, PRMT9, ASH1L, DOT1L, DNMT1, DNMT3A/3L, DNMT3B/3L | Substrate |
|  | 5TUZ[20]  5TUY[20] | MS0124 | EHMT1  IC_50_ = 13nM[20]  EHMT2  IC_50_ = 440nM[20] | <10% Inh. at 10µM for:[20]  SUV39H1, SUV39H2, SETDB1, SETD7, KMT5A, KMT5B, KMT5C, KMT2A, KMT2C, PRDM9, SMYD2, SMYD3, PRC2, SETD2, PRMT1, PRMT3, CARM1, PRMT5, PRMT6, PRMT7, PRMT8, PRMT9, ASH1L, DOT1L, DNMT1, DNMT3A/3L, DNMT3B/3L, BCDIN3D, METTL3-14 | Substrate |
|  | 5VSD[21]  5VSC[21] | MS3748 | EHMT1  IC_50_ = 5nM[21]  EHMT2  IC_50_ = 295nM[21] |  | Substrate |
|  | 5VSF[21]  5VSE[21] | MS3745 | EHMT1  IC_50_ = 4nM[21]  EHMT2  IC_50_ = 259nM[21] |  | Substrate |
|  | 4NVQ[22] | A-366 | EHMT2  IC_50_ = 3.3nM[22] | EHMT1  IC_50_ =38nM[22, 23]  NSD2  ~40% at 10µM[22, 23]  <20% Inh. at 1µM for:[22, 23]  SUV39H2, SETDB1, KMT2A, SETD7, PRMT1, PRMT3, PRMT5, PRMT6, PRMT8, KMT5A, EZH1, EZH2, SMYD3, DNMT1, KMT5B, KMT5C, SMYD2, SETD2,  DOT1L, NSD3 | Substrate |
|  | 5T5G[24] | MS2177 | KMT5A  IC_50_ = 1.9μM[24] |  | Substrate |
|  | 5TH7[24] | MS453 | KMT5A  IC_50_ = 6.9μM[24] | EHMT1  ~40% at 10µM[24]  <20% Inh. at 10µM for:[24]  ASH1L, DNMT1,  DNMT3A/3L, DNMT3B/3L, DOT1L, EZH2, EHMT2, KMT2A, KMT2C, PRDM9, PRMT1, PRMT3, CARM1, PRMT5, PRMT6, PRMT7, PRMT8, PRMT9, SETD2, SETD7, SETDB1, SMYD2, SMYD3, SUV39H1, SUV39H2, KMT5B, KMT5C | Substrate |
|  | 4JDS | PF-5426 | SETD7 |  | Substrate |
|  | 4JLG[25] | *R*-PFI-2 | SETD7  IC_50_ = 2.0nM[25] | EHMT1~30% at 50µM[25]  EHMT2~60% at 50µM[25]  EZH2~50% at 50µM[25]  <20% Inh. at 50µM for:[25]  SUV39H2, EZH1, KMT5B, KMT5C, KMT5A, SETD2, PRMT1, PRMT3, PRMT5, PRMT8, SETDB1, KMT2A, DOT1L, NSD2,  SMYD2, DNMT1 | Substrate |
|  | 4E47[26] | PFI-1 | SETD7  IC_50_ = 100nM[26] |  | Substrate |
|  | 5CCM[27] | EPZ030456 | SMYD3  IC_50_ = 4nM[27] | SMYD2  IC_50_ > 50µM[27]  <30% Inh. at 10µM for:[27]  DOT1L, EHMT1, EHMT2, EZH1, EZH2, NSD1, PRDM9, PRMT3, PRMT6, PRMT7, PRMT8, SETD7,  SETDB1, SUV39H1, NSD2, NSD3 | Substrate |
|  | 5CCM[27] | EPZ030456 | SMYD3  IC_50_ = 4nM[27] | SMYD2  IC_50_ > 50µM[27]  <30% Inh. at 10µM for:[27]  DOT1L, EHMT1, EHMT2, EZH1, EZH2, NSD1, PRDM9, PRMT3, PRMT6, PRMT7, PRMT8, SETD7,  SETDB1, SUV39H1, NSD2, NSD3 | Substrate |
|  | 5CCL[27] |  | SMYD3  IC_50_ = 17μM[27] |  | Substrate |
|  | 3S7B[28] | AZ-505 | SMYD2  IC_50_ = 120nM[28] | IC_50_ > 83.3µM for:[28]  SMYD3, DOT1L,  EZH2, EHMT1,  EHMT2, SETD7 | Substrate |
|  | 4WUY[29] | LLY-507 | SMYD2  IC_50_ < 15nM[29] | <30% Inh. at 50µM for:[29, 30]  SMYD3, EHMT2  EHMT1, SUV39H2, SETDB1, KMT2A, KMT2C, SETD7, KMT5A, KMT5B, KMT5C, PRMT1, PRMT3, PRMT5, PRMT6, PRMT8, PRDM9, EZH1, EZH2, NSD1, NSD2, NSD3, SETD2, DOT1L, DNMT1. | Substrate |
|  | 4YND[31] | A-893 | SMYD2  IC_50_ = 2.8nM[31] | SMYD3 ~50% at 10µM[31]  SUV39H2 ~60% at 10µM[31]  PRMT3 ~60% at 50µM[31]  PRMT7 ~60% at 50µM[31]  KMT5B ~50% at 50µM[31]  <30% Inh. at 50µM for:[31]  EHMT1, EHMT2,  SETDB1, SETD7,  KMT5A, KMT5C,  KMT2A, EZH1, EZH2, SETD2, PRDM9,  PRMT1, PRMT5, PRMT6, PRMT8, NSD1, NSD2, NSD3, ASH1L, DOT1L, DNMT1, DNMT3A/3L, DNMT3B/3L, BCDIN3D, METTL21A, METTL21D. | Substrate |
|  | 5ARF[32] |  | SMYD2  IC_50_ = 0.8µM[32] |  | Substrate |
|  | 5ARG[32] | (S)-BAY-598 | SMYD2  IC_50_ = 27nM[32] | SMYD3  IC_50_ ~ 3µM[32]  PRMT3 ~30% at 10µM[32]  PRMT7 ~25% at 50µM[32]  <20% Inh. at 1µM for:[32, 33]  EHMT2, EHMT1, PRDM9, SETDB1, SUV39H1, SUV39H2, SETD7, KMT2A, KMT2C, KMT5A, PRMT1, PRMT5, PRMT6, PRMT8, SETD2, EZH2, KMT5B, KMT5C, BCDNIMT1, DNMT1, DNMT3A/3L, DNMT3B/3L, ASH1L, DOT1L, NSD1, NSD2, NSD3, METTL21A, METTL21D. | Substrate |
|  | 5KJK[34] | AZ370 | SMYD2  IC_50_ = 12nM[34] |  | Substrate |
|  | 5KJM[34] | AZ931 | SMYD2  IC_50_ = 65nM[34] |  | Substrate |
|  | 5KJN[34] | AZ506 | SMYD2  IC_50_ = 17nM[34] | DOT1L  IC_50_ > 100µM[34] | Substrate |
|  | 5CPR[35] | A-196 | KMT5B  IC_50_ = 25nM[35] | KMT5C  IC_50_ = 144nM[35]  <20% Inh. at 1µM for:[35, 36]  EHMT2, EHMT1, SUV39H1, SUV39H2, SETDB1, SETD7, KMT5A, KMT2A, KMT2C, PRMT1, PRMT3, PRMT5,  PRMT6, PRMT7,  PRMT8, PRDM9, SMYD2, SMYD3, EZH2, SETD2, BCDIN3D, DNMT1, DNMT3A/3L, DNMT3B/3L, ASH1L, DOT1L, NSD1, NSD2, NSD3 | Substrate |
|  | 5WBV |  | KMT5B |  | Substrate |

(1) The resolved co-crystallized ligand in the corresponding PDB entry does not match this structure: N2-ethyl-6,7-dimethoxy-N4-(4-piperidyl)quinazoline-2,4-diamine for 5TTF and N2-ethyl-6,7-dimethoxy-N4-(1-methyl-4-piperidyl)quinazoline-2,4-diamine for 5TTG.

**Figure S1.** View of the cofactor molecules (SAM/SAH/competitive inhibitors) and peptides (ribbon) for the different proteins after structurally superposing all 104 PDB entries of PKMTs using the full length alignment available at ChromoHub [37]. There is a total lack of overlap between the different cofactor molecules.


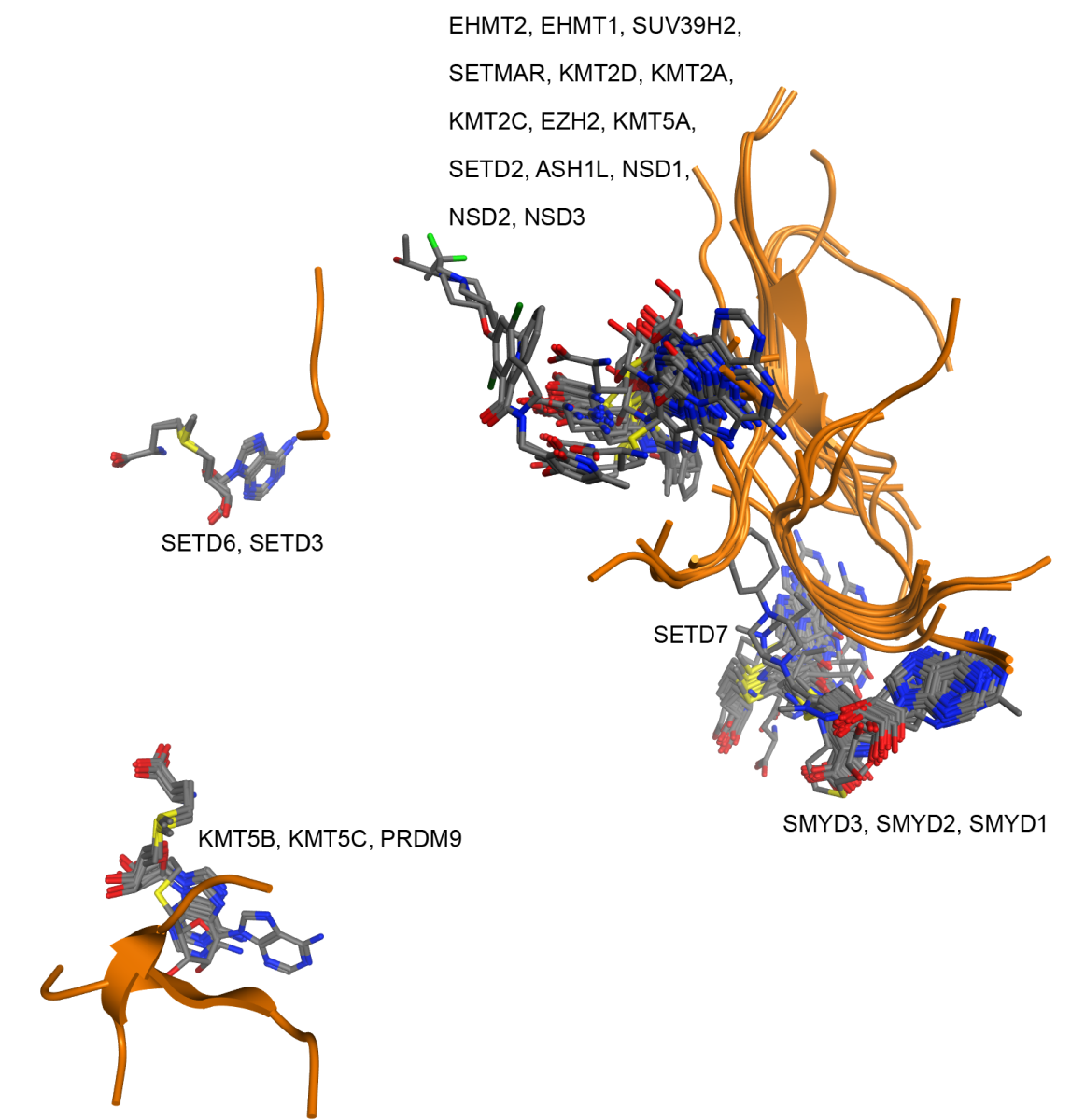


**Figure S2.** View of the cofactor molecules (SAM/SAH/competitive inhibitors) and peptides (ribbon) for the different proteins after structurally superposing all 104 PDB entries of PKMTs using the MT domain of PKMTs alignment available at ChromoHub [37].


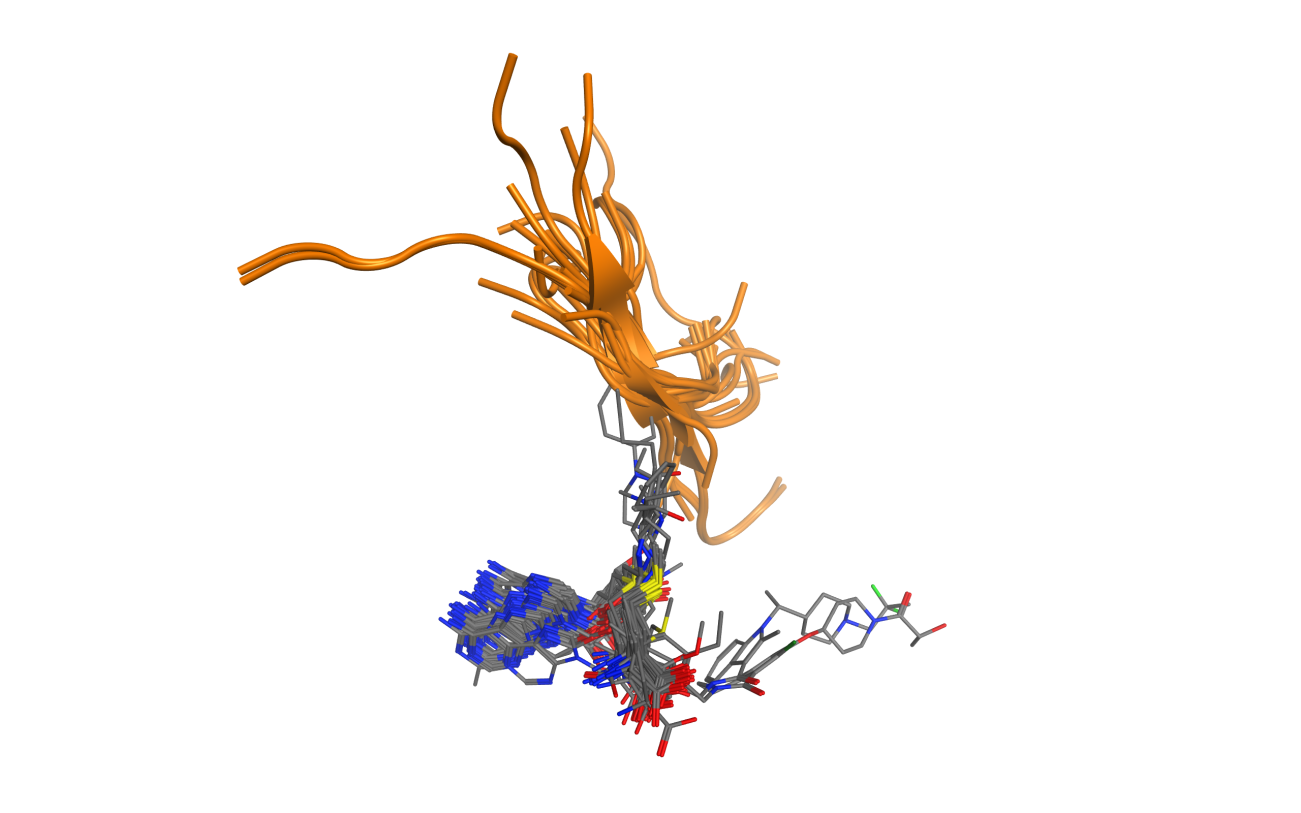


**Figure S3.** Overlay of the 23 PKMT structures (one selected PDB per structure) for the different proteins after structurally superposing all 104 PDB entries of PKMTs using the MT domain of PKMTs alignment available at ChromoHub [37]. For clarity, the different PKMTs were split into different groups according to additional co-crystallized domains beyond SET. SAM/SAH cofactors are shown.

**
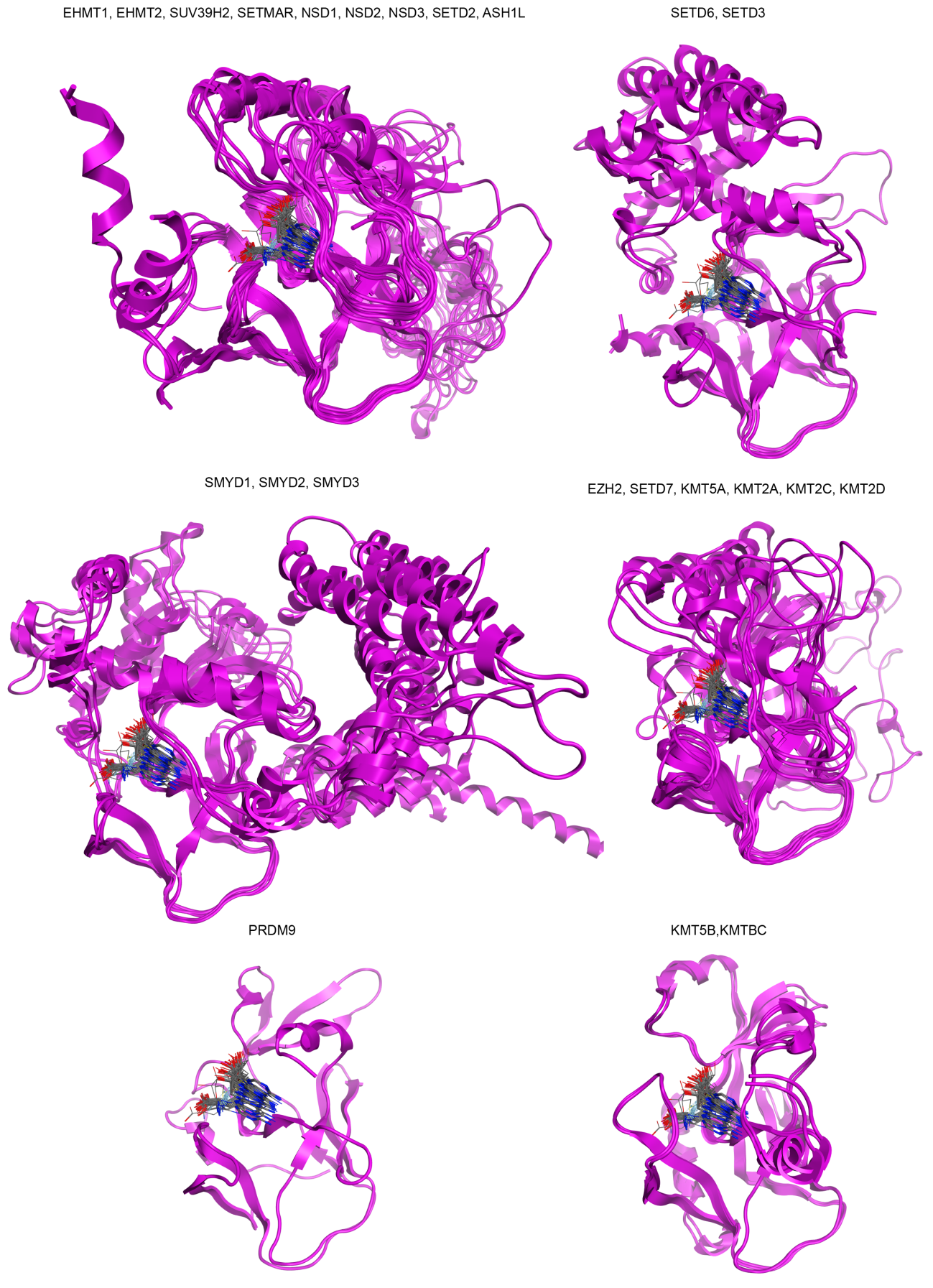
**

**Figure S4**. Pairwise matrix of RMSD values of superposition of the 101 complexes with cofactor (SAM/SAH) and SAM-competitive inhibitors with 23 PKMTs. Upper panel corresponds to the superposition of gapless residues (61) and bottom graph to all residues of the proteins.


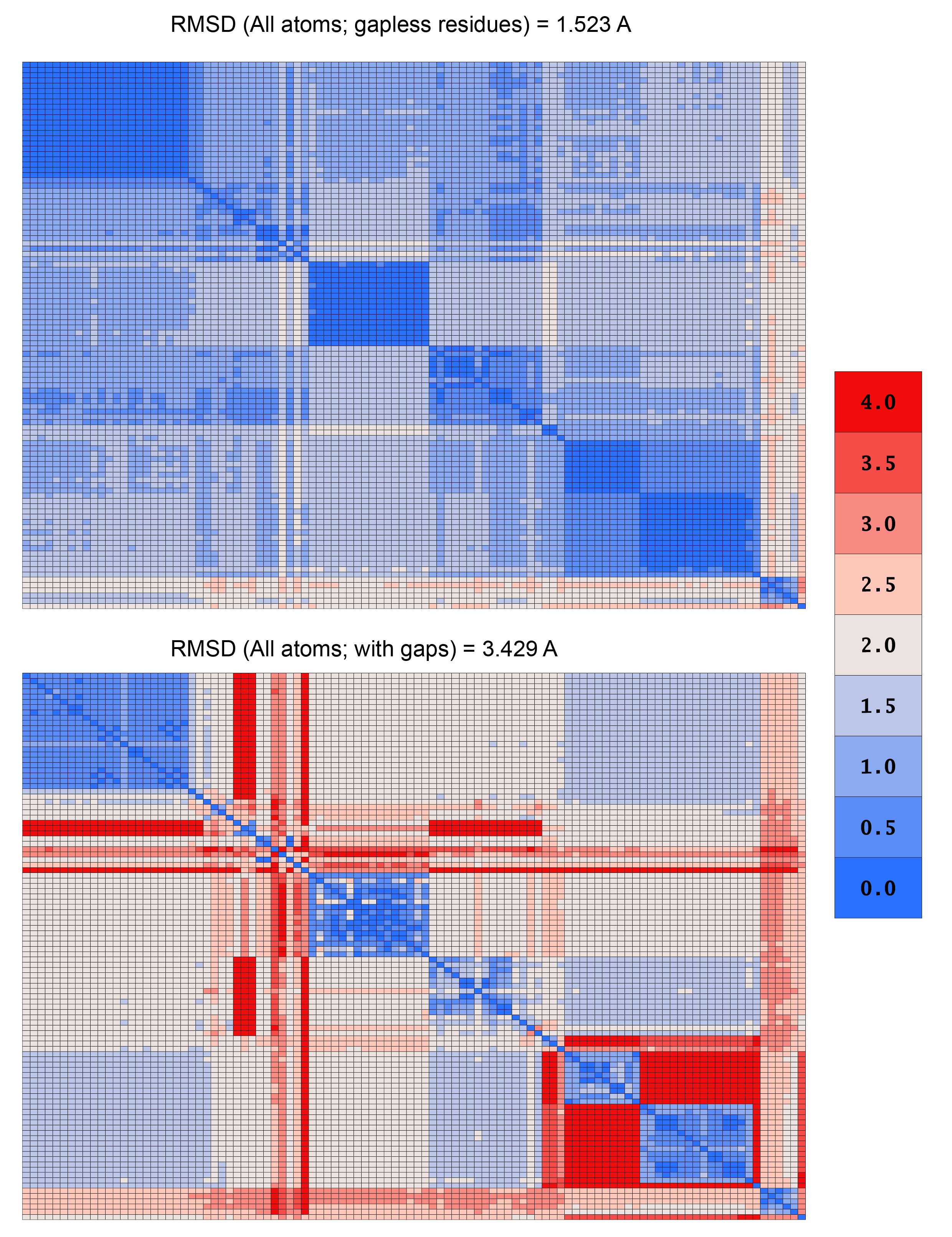


**Table S3**. Translation of sequence alignment positions to PDB residue number for each of the 10 relevant PKMTs with cofactor bound structures discussed in the text.

| Alig.  Pos | EHMT1 | SETD2 | SETD7 | SMYD3 | EZH2 | PRDM9 | KMT5B | KMT5A | NSD1 | KMT2A |
| --- | --- | --- | --- | --- | --- | --- | --- | --- | --- | --- |
| 545 | M1105 | K1560 | S225 | R14 | A622 | --- | Y203 | K267 | R101 | H3839 |
| 549 | --- | --- | --- | --- | --- | --- | S205 | --- | --- | --- |
| 550 | --- | --- | --- | --- | --- | --- | E206 | --- | --- | --- |
| 556 | G1106 | G1561 | A226 | G15 | G623 | --- | --- | G268 | G102 | G3840 |
| 557 | W1107 | W1562 | G227 | N16 | W624 | A256 | G209 | R269 | W103 | R3841 |
| 558 | --- | --- | E228 | --- | --- | --- | --- | --- | --- | --- |
| 559 | G1108 | G1563 | G229 | G17 | G625 | G257 | A210 | G270 | G104 | G3842 |
| 926 | --- | --- | --- | E130 | --- | --- | --- | --- | --- | --- |
| 928 | --- | --- | --- | N132 | --- | --- | --- | --- | --- | --- |
| 1116 | --- | N1601 | --- | --- | Y661 | --- | --- | S308 | I142 | I3880 |
| 1117 | --- | I1602 | --- | --- | M662 | --- | --- | T309 | T143 | G3881 |
| 1119 | E1139 | H1603 | A261 | --- | C663 | --- | H245 | G310 | N144 | C3882 |
| 1124 | D1140 | Y1604 | N263 | C180 | S664 | --- | D249 | C311 | F145 | Y3883 |
| 1130 | S1141 | Y1605 | G264 | N181 | F665 | S289 | F250 | Y312 | Y146 | M3884 |
| 1147 | Y1142 | F1606 | N265 | S182 | L666 | Y291 | S251 | M313 | M147 | --- |
| 1358 | R1166 | R1625 | H293 | S202 | R685 | R317 | A269 | R336 | R166 | R3903 |
| 1359 | F1167 | F1626 | K294 | L203 | F686 | Y318 | F270 | L337 | F167 | F3904 |
| 1360 | I1168 | M1627 | A295 | L204 | A687 | V319 | I271 | I338 | M168 | I3905 |
| 1361 | N1169 | N1628 | N296 | N205 | N688 | N320 | N272 | N339 | N169 | N3906 |
| 1362 | H1170 | H1629 | H297 | H206 | H689 | C321 | H273 | H340 | H170 | H3907 |
| 1473 | Y1211 | Y1666 | Y335 | Y239 | Y726 | Y357 | Y307 | Y377 | Y207 | Y3944 |
| 1497 | --- | --- | --- | Y257 | --- | --- | --- | I385 | --- | --- |
| 1521 | --- | R1670 | --- | --- | Q730 | --- | --- | --- | C211 | --- |
| 1586 | W1216 | --- | --- | --- | --- | --- | F312 | --- | --- | --- |
| 1599 | --- | --- | --- | --- | --- | --- | --- | --- | --- | --- |
| 1601 | F1223 | Q1676 | W352 | C258 | L734 | --- | --- | H388 | T217 | L3955 |
| 1605 | --- | --- | E356 | --- | --- | --- | --- | --- | --- | --- |
| 1612 | C1225 | C1678 | T363 | E260 | Y736 | --- | C319 | W390 | C219 | C3957 |
| 1613 | --- | --- | --- | C261 | --- | --- | --- | --- | --- | --- |
| 1619 | R1226 | F1679 | --- | --- | V737 | --- | E320 | L391 | K220 | N3958 |

**Table S4.** Translation of sequence alignment positions to PDB residue number for each of the 7 PKMTs with substrate-competitive inhibitors discussed in the text.

| Alignment Position | EHMT2 | EHMT1 | SETD7 | KMT5A | KMT5B | SMYD2 | SMYD3 |
| --- | --- | --- | --- | --- | --- | --- | --- |
| 831 | --- | --- | --- | --- | --- | T105 | S101 |
| 972 | --- | --- | --- | --- | --- | K145 | K140 |
| 1063 | D1074 | D1131 | H252 | I293 | --- | --- | --- |
| 1087 | A1077 | A1134 | V255 | A296 | --- | --- | --- |
| 1088 | --- | --- | D256 | --- | --- | --- | --- |
| 1089 | D1078 | D1135 | S257 | K297 | --- | --- | --- |
| 1097 | R1080 | R1137 | D259 | K299 | L243 | --- | --- |
| 1114 | --- | --- | --- | --- | --- | N180 | I179 |
| 1124 | D1083 | D1140 | N263 | C311 | D249 | C181 | C180 |
| 1130 | S1084 | S1141 | G264 | Y312 | F250 | N182 | N181 |
| 1147 | Y1085 | Y1142 | N265 | M313 | S251 | G183 | S182 |
| 1148 | L1086 | L1143 | T266 | Y314 | V252 | F184 | F183 |
| 1149 | F1087 | F1144 | L267 | --- | M253 | --- | --- |
| 1151 | D1088 | D1145 | S268 | Y315 | S255 | T185 | T184 |
| 1197 | --- | --- | --- | --- | --- | E187 | C186 |
| 1208 | --- | --- | --- | --- | --- | H193 | E192 |
| 1256 | --- | --- | --- | --- | W264 | --- | --- |
| 1325 | C1098 | C1155 | V274 | C324 | --- | S196 | V195 |
| 1358 | R1109 | R1166 | H293 | R336 | A269 | A203 | S202 |
| 1473 | Y1154 | Y1211 | Y335 | Y377 | Y307 | Y240 | Y239 |
| 1476 | G1155 | G1212 | G336 | --- | G308 | I241 | L240 |
| 1496 | --- | --- | --- | --- | --- | S257 | Q256 |
| 1576 | R1157 | R1214 | D338 | --- | G310 | --- | --- |
| 1585 | F1158 | F1215 | --- | --- | F311 | --- | --- |

**Table S5.** Chemical structure, biochemical inhibitory activity against primary target of cofactor and substrate-competitive inhibitors of PKMTs compiled from ChEMBL and literature and lacking a crystal structure.

| Chemical Structure | Ligand  Name | Primary Target | Selectivity | Binding  Site |
| --- | --- | --- | --- | --- |
|  | Cyproheptadine | SETD7  IC_50_ = 1µM[38] | EHMT2  IC_50_ > 100µM[38]  KMT5A  IC_50_ > 100µM[38] | Substrate |
|  | BRD9539 | EHMT2  IC_50_ = 6.3µM[39] | PRC2  IC_50_ ~ 5µM[39]  NSD2  IC_50_ = 40µM[39]  <20% Inh. at 10µM for:[39]  SUV39H1, SUV39H2, KMT2A, SETD7, KMT5A, PRMT1, PRMT3, PRMT5, DNMT1 | SAM |
|  | DC-S239[40] | SETD7  IC_50_ = 4590nM[40] | KMT5A ~41% at 100µM[40]  <30% Inh. at 100µM for:[40]  EHMT2, NSD1  EZH2 | SAM |
|  | DC-S238[40] | SETD7  IC_50_ = 4880nM[40] | KMT5A ~46% at 100µM[40]  <30% Inh. at 10µM for:[40]  EHMT2, NSD1, EZH2 | SAM |
|  | SETin-1[41] | SETD7  IC_50_ = 9930nM[41] | EHMT2  IC_50_ = 26400nM[41] | SAM |
|  | GSK343[42] | EZH2  IC_50_ = 4nM[42] | EZH1  IC_50_ = 240nM[42]  SETD7  IC_50_ = 63µM[42]  SUV39H2  IC_50_ = 123µM[42]  PRMT3  IC_50_ = 74µM[42]  IC_50_ > 100µM for:[42]  SETMAR, EHMT2, DNMT1, DNMT3A, DNMT3B, DOT1L,  KMT2A, KMT2B  KMT2C, KMT2D  PRMT1, CARM1  PRMT5, SUV39H1 | SAM |
|  | UNC1999[43] | EZH2  IC_50_ < 10nM[43] | EZH1  IC_50_ = 45 nM  IC_50_ > 100µM for:[43]  SMYD2, EHMT2, SUV39H2, PRMT5, KMT5A, SETD7, PRMT3, KMT2A, PRMT1, KMT5C,  SETDB1, EHMT1,  DOT1L | SAM |

**Table S6.** Distribution of PLIF detected contacts for the 101 complexes of SET domain-containing PKMTs bound with cofactor (SAM/SAH) or SAM-competitive inhibitors. % corresponds to # crystals divided by the number of complexes (101). HBD = Hydrogen Bond Donor. HBA = Hydrogen Bond Acceptor.

| Alignment Position | Contact Type | # Crystals | % | PKMTs involved |
| --- | --- | --- | --- | --- |
| 545 | HBD | 21 | 20.8 | EHMT1/KMT2A/KMT2C  SETMAR/EZH2/KMT5A  SETD2/SETD3/SMYD2  SMYD3 |
| 545 | Surface Contacts | 14 | 13.9 | EHMT2/EHMT1/SETMAR  KMT2A/KMT5A/SETD2  SETD3/SMYD3 |
| 545 | Arene Attraction | 10 | 9.9 | EHMT2/EHMT1/KMT2A  KMT5A/NSD1/SMYD3 |
| 549 | HBA | 2 | 2.0 | KMT5B |
| 549 | HBD | 2 | 2.0 | KMT5B |
| 550 | HBD | 5 | 5.0 | KMT5B/KMT5C |
| 550 | Ionic | 5 | 5.0 | KMT5B/KMT5C |
| 556 | HBA | 38 | 37.6 | EHMT2/EHMT1/SUV39H2  KMT2D/KMT2A/KMT2C  EZH2/SETD2/ASH1L/  NSD1/NSD3/SETD6  SMYD3/SMYD2 |
| 556 | HBD | 5 | 5.0 | EHMT1/SETD7 |
| 556 | Arene Attraction | 11 | 10.9 | SETD7 |
| 557 | HBD | 72 | 71.3 | EHMT2/EHMT1/SUV39H2  SETMAR/EZH2  KMT2A/KMT2C/KMT2D KMT5A /SETD3  SETD2/ASH1L/NSD2  NSD1/NSD3/SETD6  SMYD3/SMYD2/SMYD1 |
| 557 | HBA | 79 | 78.2 | KMT5B/EHMT2/EHMT1  SUV39H2/SETMAR/KMT2D  KMT2A/KMT2C/EZH2/KMT5A  SETD7/SETD2/NSD2/SETD3  NSD3/NSD1/SETD6  SMYD3/SMYD2  SMYD1/KMT5C |
| 557 | Ionic | 18 | 17.8 | KMT5A/SMYD2/SMYD1 |
| 557 | Surface Contacts | 8 | 7.9 | SMYD2 |
| 558 | HBD | 14 | 13.9 | SETD7 |
| 558 | HBA | 15 | 14.9 | SETD7 |
| 559 | HBD | 5 | 5.0 | KMT5B/KMT5C |
| 559 | HBA | 6 | 5.9 | KMT5B/KMT5C/PRDM9 |
| 926 | HBD | 10 | 9.9 | SMYD3/SMYD2/SMYD1 |
| 926 | Surface Contacts | 15 | 14.9 | SMYD3/SMYD2/SMYD1 |
| 928 | HBD | 10 | 9.9 | SMYD3 |
| 928 | HBA | 21 | 20.8 | SMYD3/SMYD2/SMYD1 |
| 1116 | Surface Contacts | 2 | 2.0 | EZH2 |
| 1117 | HBD | 4 | 4.0 | EZH2/NSD1/NSD3/NSD2 |
| 1119 | HBA | 16 | 15.8 | EZH2/SETMAR/SETD2  ASH1L/NSD1  NSD3/NSD2 |
| 1119 | Surface Contacts | 14 | 13.9 | SETD2/ASH1L/NSD1  NSD3/NSD2 |
| 1119 | HBD | 12 | 11.9 | SETD2/ASH1L  NSD1/NSD3 |
| 1124 | HBD | 41 | 40.6 | KMT5A/SETD2/SETD3  NSD2/NSD3  NSD1/SETD6/ASH1L  SMYD3/SMYD2 |
| 1124 | HBA | 29 | 28.7 | KMT5A/SETD2/SETD3  KMT2D/KMT2A/KMT2C  NSD2/NSD3/NSD1  SETMAR/EZH2/ASH1L  SMYD3/SMYD2 |
| 1130 | HBA | 19 | 18.8 | KMT5A/EHMT2/EHMT1  SETD7/SETD2  SETD3/NSD2/SETD6  SMYD2/KMT5C |
| 1130 | Surface Contacts | 11 | 10.9 | KMT5A/KMT5B/SETD7  SETD6  EZH2/KMT5C |
| 1130 | HBD | 43 | 42.6 | KMT5B/EHMT2/EHMT1  SUV39H2/SETD7/SETD2  SMYD3/SMYD2/SMYD1  KMT5C |
| 1147 | HBA | 20 | 19.8 | EHMT2/EHMT1/PRDM9 |
| 1147 | Surface Contacts | 4 | 4.0 | EHMT1/PRDM9 |
| 1147 | HBD | 5 | 5.0 | KMT5B/SETD7/KMT5C |
| 1358 | HBD | 88 | 87.1 | KMT5B/EHMT2/EHMT1  SUV39H2/SETMAR/KMT2A/  KMT2D/EZH2/KMT5A/SETD7  SETD2/NSD2/ASH1L/NSD3  NSD1/SETD6/SETD3  SMYD3/SMYD2/SMYD1  KMT5C/PRMD9 |
| 1359 | HBA | 16 | 15.8 | SETD7 |
| 1359 | Ionic | 16 | 15.8 | SETD7 |
| 1359 | HBD | 8 | 7.9 | KMT5A/SMYD2/SETD6 |
| 1360 | HBD | 40 | 39.6 | KMT5B/EHMT2/EHMT1  SUV39H2/SETD2/SETD3  KMT5A/SETD7/ASH1L  NSD2/SETD6  SMYD3/SMYD2/KMT5C |
| 1361 | HBD | 87 | 86.1 | KMT5B/EHMT2/EHMT1  SETMAR/KMT2A/KMT2D  KMT5A/SETD7/SETD3  SETD2/ASH1L/NSD3/SETD6  SMYD3/SMYD2/SMYD1  KMT5C/PRMD9 |
| 1361 | HBA | 2 | 2.0 | SETD7 |
| 1362 | HBD | 95 | 94.1 | KMT5B/EHMT2/EHMT1  SUV39H2/SETMAR/EZH2  KMT2A/KMT2C/KMT2D  KMT5A/SETD7/SETD3  SETD2/ASH1L/NSD2/NSD1  NSD3/SETD6  SMYD3/SMYD2  SMYD1/KMT5C/PRMD9 |
| 1362 | HBA | 99 | 98.0 | KMT5B/EHMT2/EHMT1  SUV39H2/SETMAR/EZH2  KMT2A/KMT2C/KMT2D  KMT5A/SETD7/SETD3/SETD2  ASH1L/NSD2/NSD1/NSD3  SETD6/SMYD3/SMYD2  SMYD1/KMT5C/PRMD9 |
| 1473 | Surface Contacts | 42 | 41.6 | EHMT2/EHMT1  KMT2D/KMT2A/KMT2C  EZH2/KMT5A/SETD7/SETD2  ASH1L/NSD2/NSD1  SMYD2/SMYD3/SMYD1 |
| 1473 | HBD | 7 | 6.9 | SUV39H2/EHMT2  SETD7/SETD2/ASH1L |
| 1497 | HBD | 24 | 23.8 | SMYD3/SMYD2  SMYD1 |
| 1497 | HBA | 2 | 2.0 | SMYD2 |
| 1521 | HBA | 2 | 2.0 | SETD2/SETMAR |
| 1586 | Surface Contacts | 3 | 3.0 | KMT5B/KMT5C |
| 1599 | HBD | 2 | 2.0 | SETD6 |
| 1601 | HBA | 7 | 6.9 | SETD2 |
| 1601 | Surface Contacts | 8 | 7.9 | SETD7/SETD2/KMT2A  KMT2D/NSD3/SUV39H2 |
| 1601 | HBD | 7 | 6.9 | SETD2/ASH1L |
| 1605 | HBD | 10 | 9.9 | SETD7 |
| 1612 | HBD | 48 | 47.5 | KMT5B/EHMT2/EHMT1  SETMAR/SUV39H2  ASH1L/SETD2/NSD3  NSD1/KMT2C/NSD2  KMT2D/KMT2A/KMT5C |
| 1612 | Surface Contacts | 4 | 4.0 | KMT5A |
| 1612 | HBA | 46 | 45.5 | KMT5B/EHMT2/EHMT1  SETMAR/SUV39H2  EZH2/ASH1L/SETD2  NSD3/NSD1/KMT2C/NSD2  KMT2A/KMT5C |
| 1613 | HBD | 3 | 3.0 | SMYD2 |
| 1619 | HBA | 48 | 47.5 | KMT5B/EHMT2/EHMT1  SETMAR/SUV39H2  ASH1L/SETD2/NSD3  NSD1/KMT2C/NSD2  KMT2D/KMT2A/KMT5C |
| **TOTAL** |  | **1320** |  |  |

**Figure S5**. Mapping of PLIF interacting residues for 6 complexes of representative PKMTs complexed with either SAM or SAH. SETD7 (PDB: 1N6C; SAM); KMT2A (PDB: 2W5Z; SAH); NSD1 (PDB: 3OOI; SAM); SMYD3 (PDB: 3QWP; SAM); EHMT1 (PDB: 5VSD; SAM) and SETD2 (PDB: 4H12; SAM). All 30 PLIF interacting residues for SAM-competitive inhibitors are shown, considering that the residue can be mapped in the alignment. Balls correspond to either backbone (red) or side chain (green) atoms establishing HB contacts or hydrophobic contacts (blue), respectively. Grey balls, marked at the Cα atom, correspond to PLIF interacting residues for which the corresponding ligand does not establish any interaction.


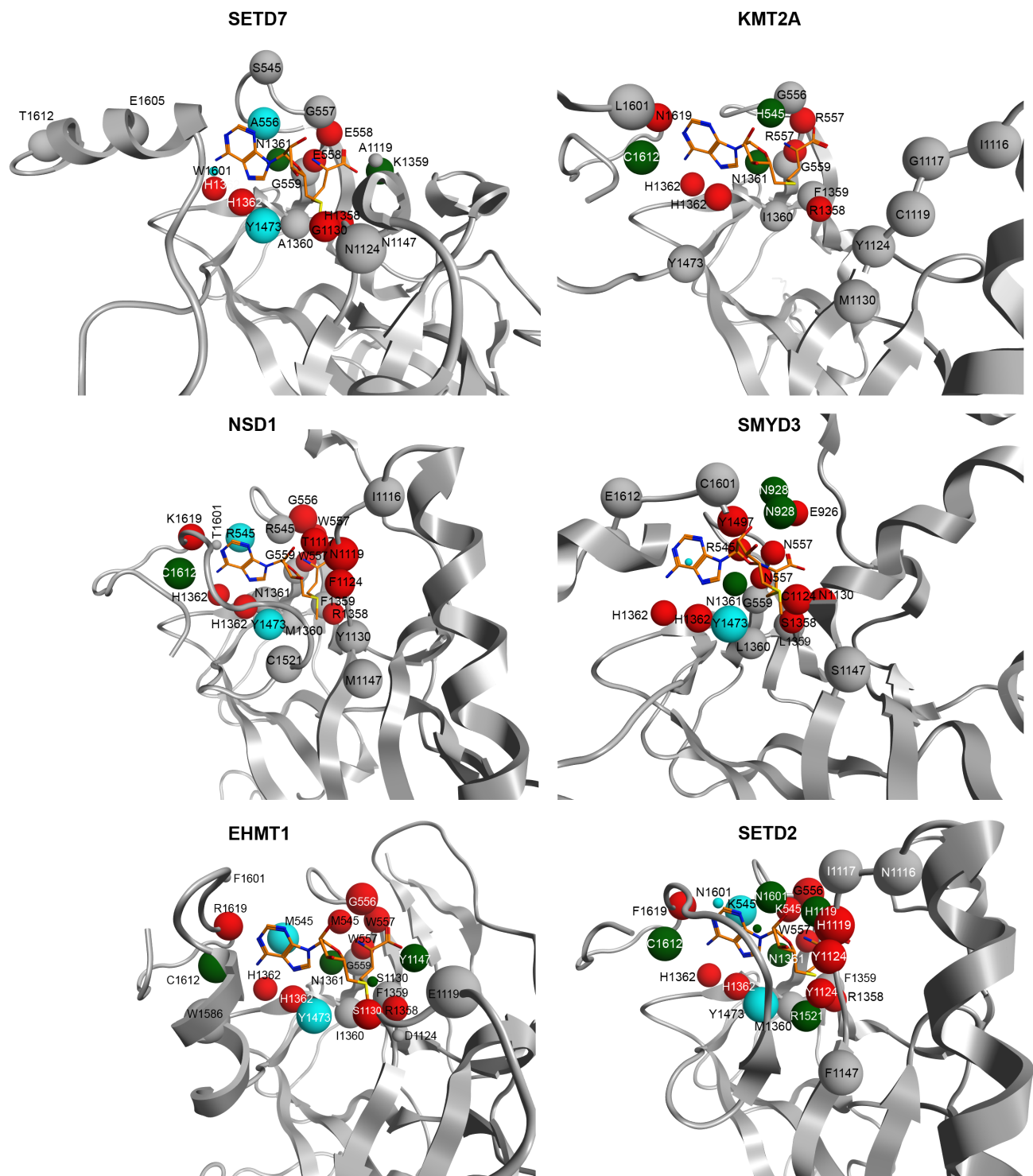


**Figure S6**. 2D schematic view of PLIF conserved contacts across the 101 complexes of the cofactor (SAM or SAH) and SAM-competitive inhibitors against 23 different PKMTs (EHMT2, EHMT1, SUV39H2, SETMAR, KMT2D, KMT2A, KMT2C, EZH2, KMT5A, SETD7, SETD2, ASH1L, NSD3, NSD1, NSD2, SETD6, SETD3, SMYD3, SMYD2, SMYD1, KMT5B, KMT5C and PRMD9). Circles and dotted lines correspond to hydrogen bond contacts and surface contact interactions (affecting different atoms), respectively. Residue numbers in the legend correspond to the alignment position of the corresponding amino acid residue in the PKMT alignment (not to the PDB number).


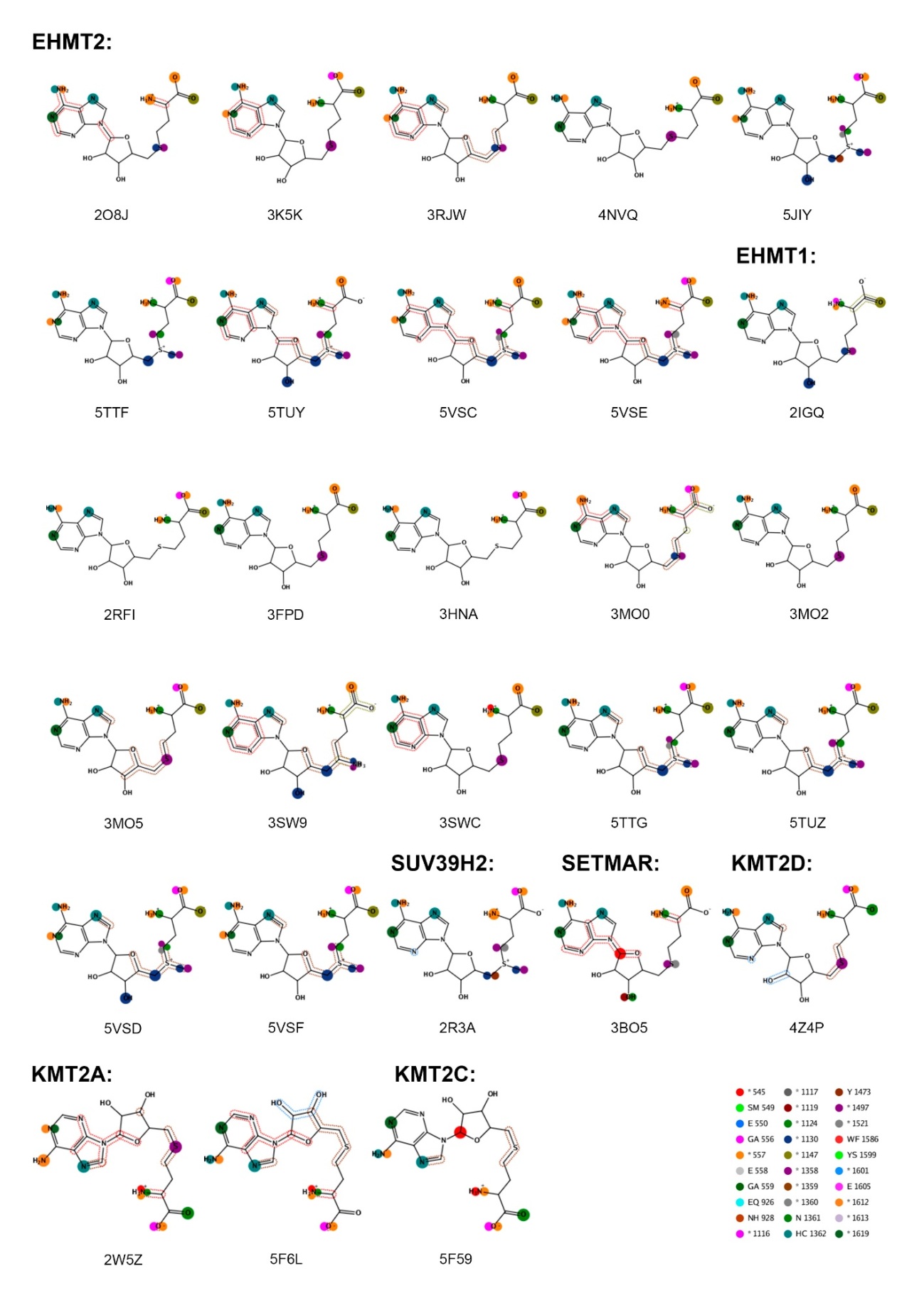


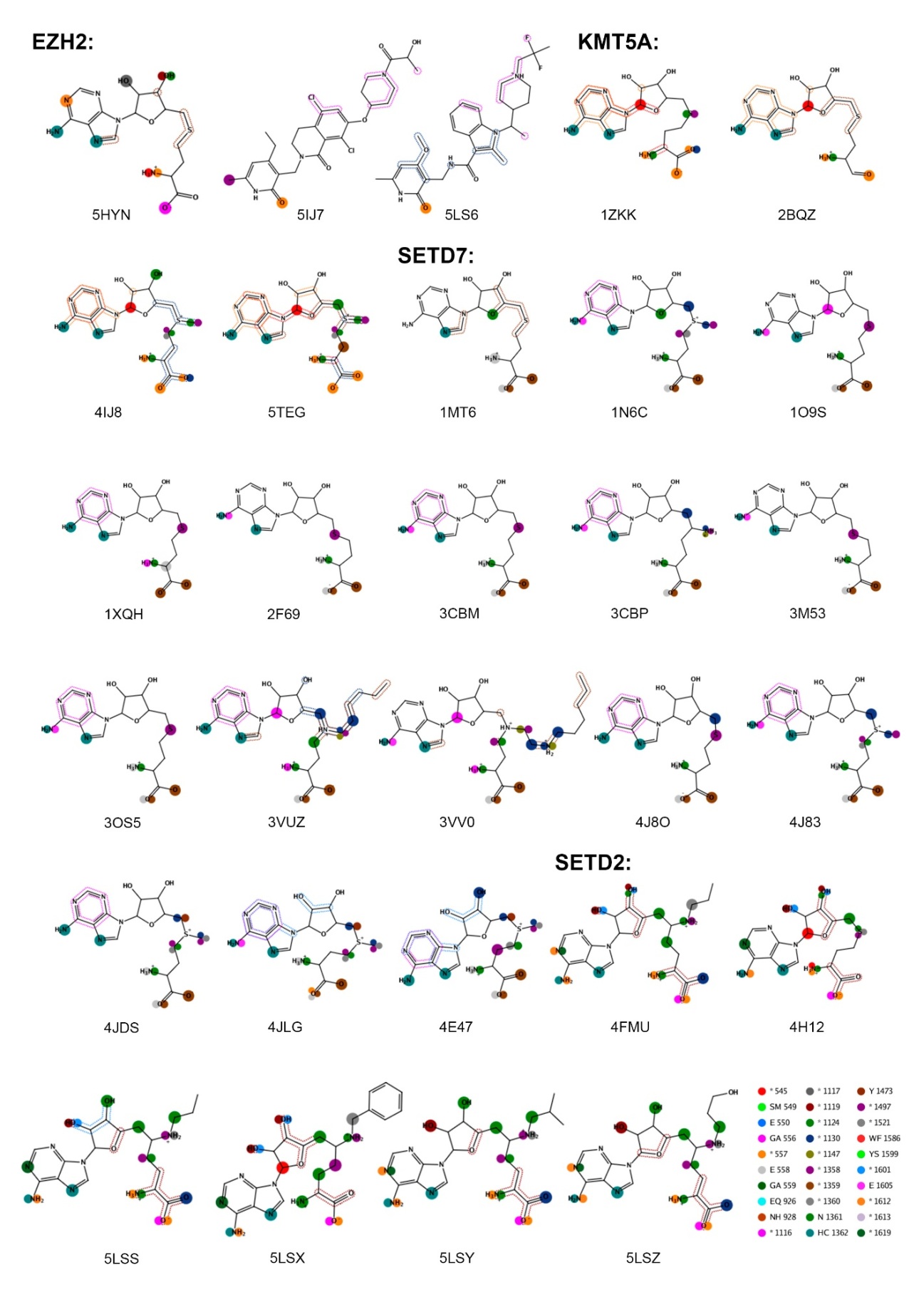


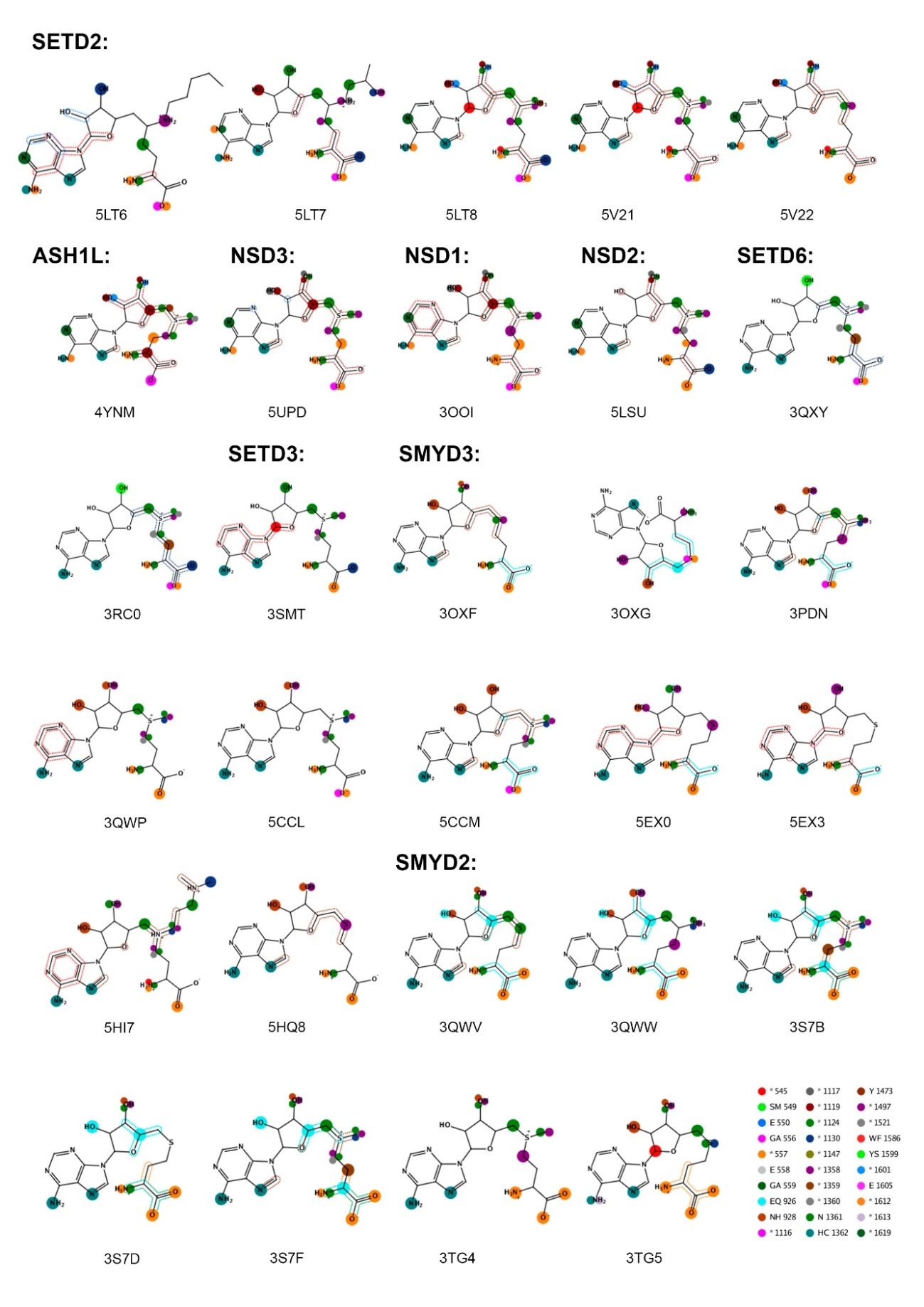


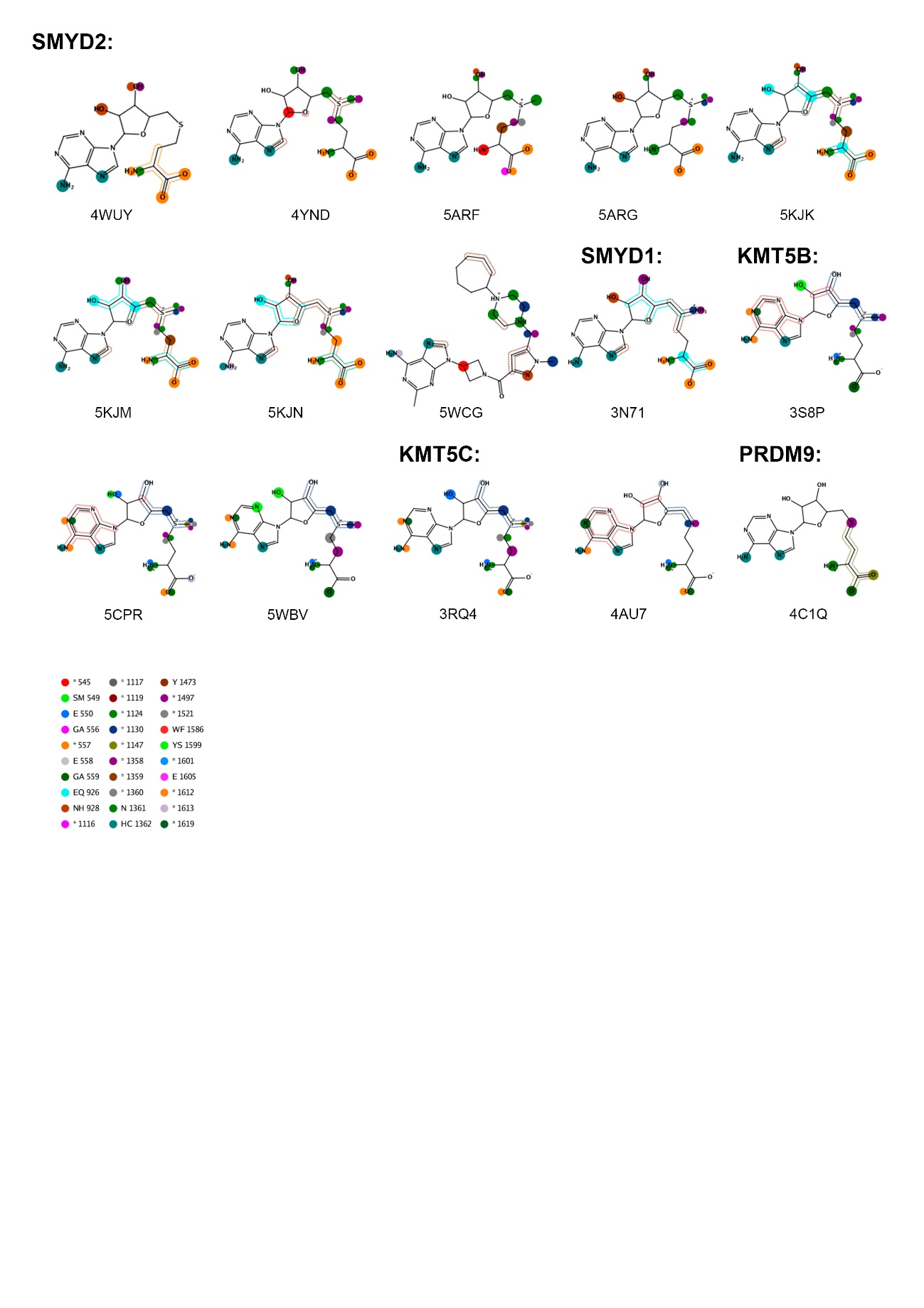


**Figure S7**. Heat map colored according to the RMSD of superposition between the 41 SAM cofactors in each of the 41 PKMT-SAM complexes. For comparison with Figure S8 below, clustering is based on PLIF distances. With the exception of SMYD2-SAM complex (PDB entry 5ARG) [32], the pairwise RMSD of superposition is < 1 Å for the rest of the cases.


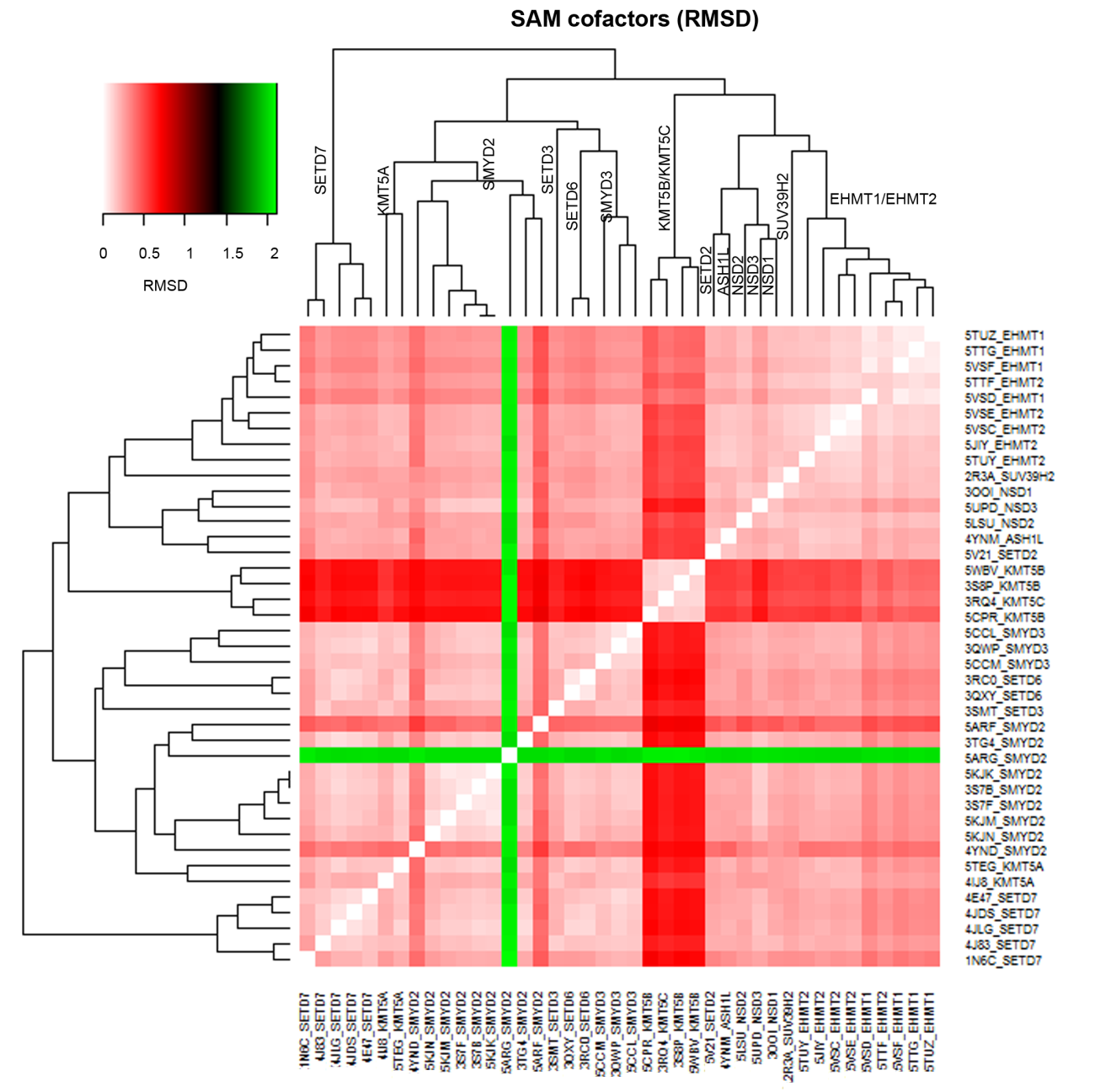


**Figure S8.** Heat map representation of PLIF similarities (expressed as distances) for the 41 PKMT-SAM complexes.


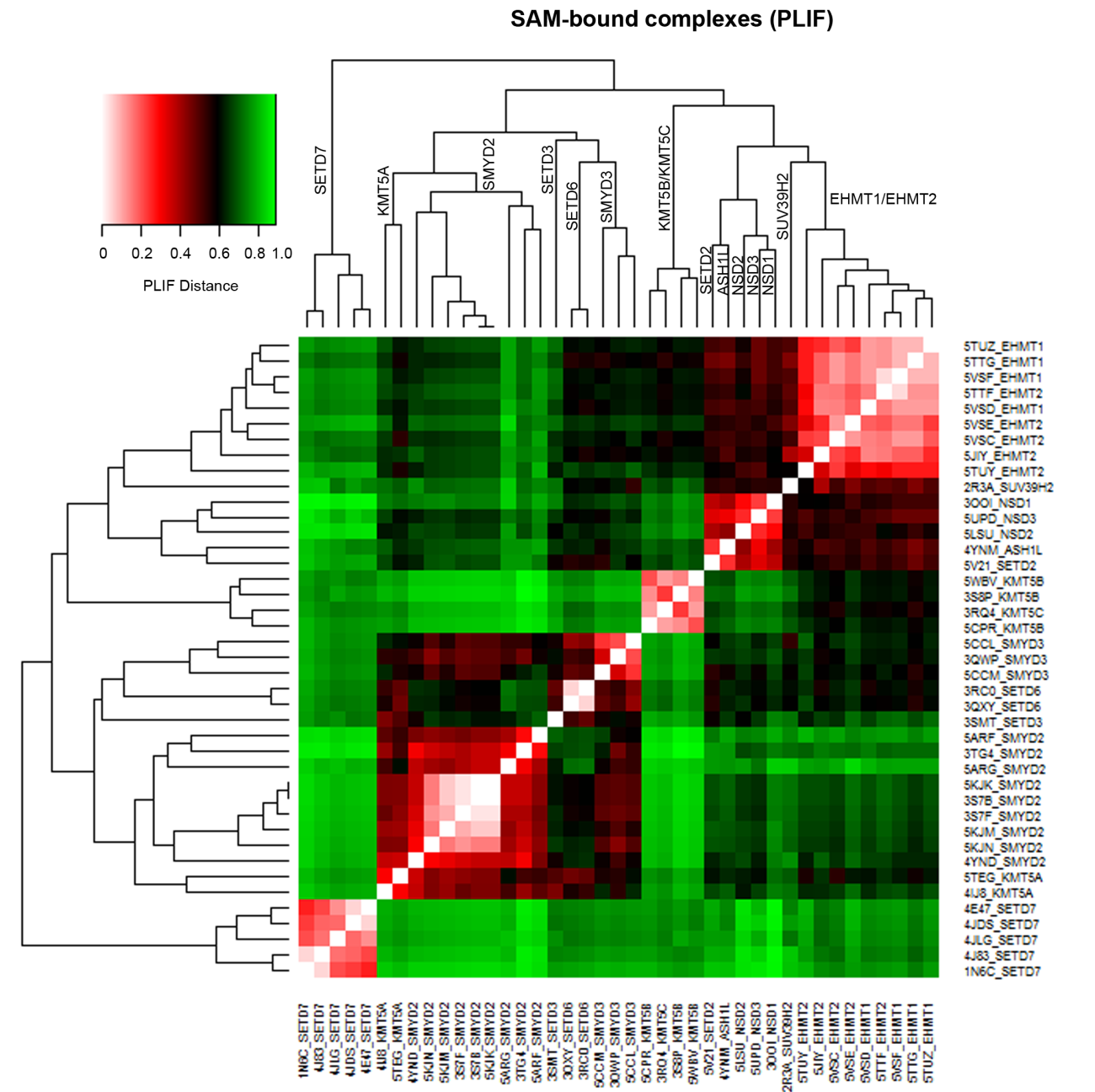


**Figure S9.** Heat map colored according to the RMSD of superposition between the 41 SAH cofactors in each of the 41 PKMT-SAH complexes. For comparison with Figure S10 below, clustering is based on PLIF distances. With the exception of SMYD3-SAH complex (PDB entry 3OXG) [44], in which the ribose-*l*-homocysteine is flipped, the pairwise RMSD of superposition is < 1 Å for the rest of the cases.


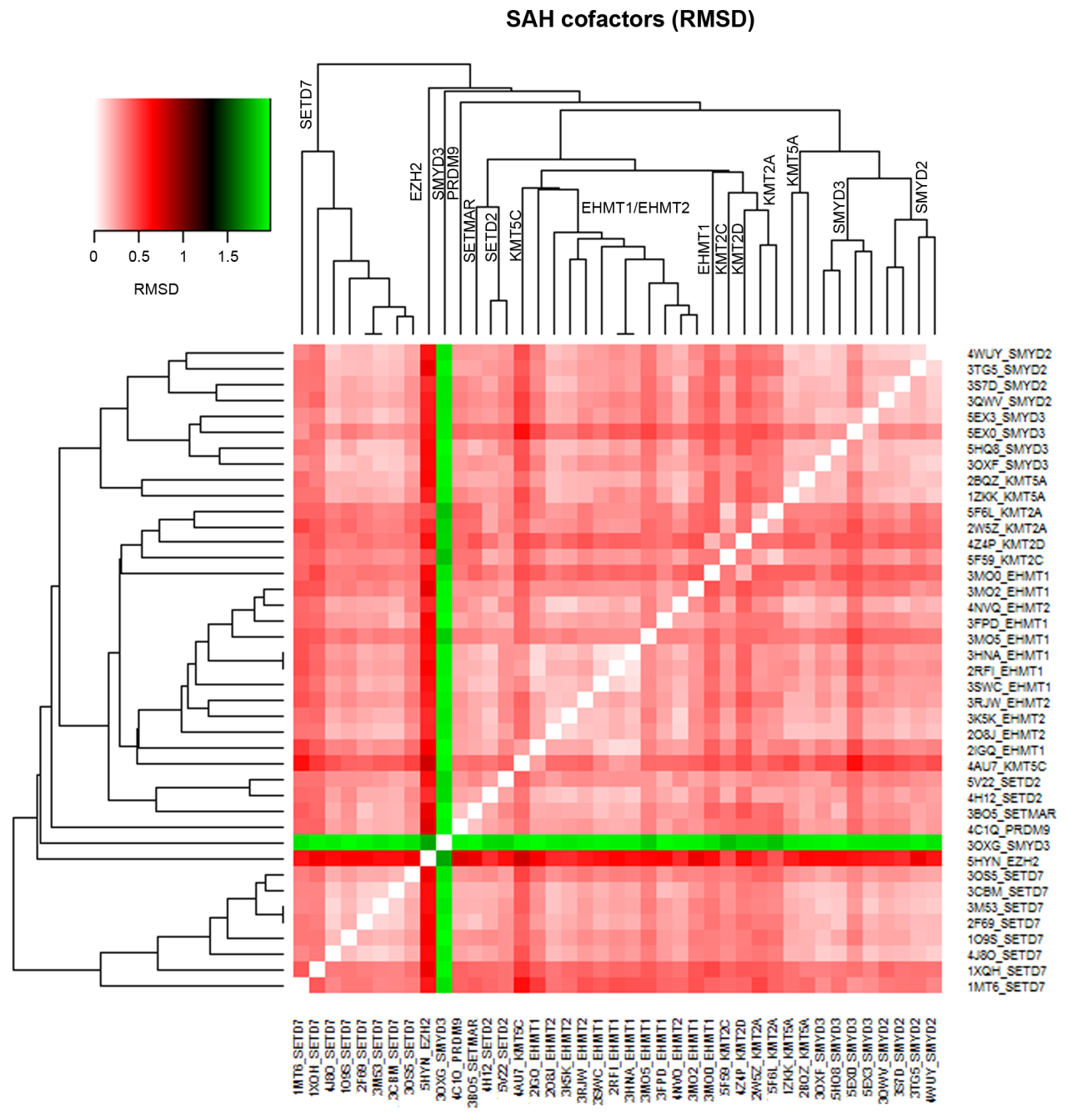


**Figure S10.** Heat map representation of PLIF similarities (expressed as distances) for the 41 PKMT-SAH complexes.


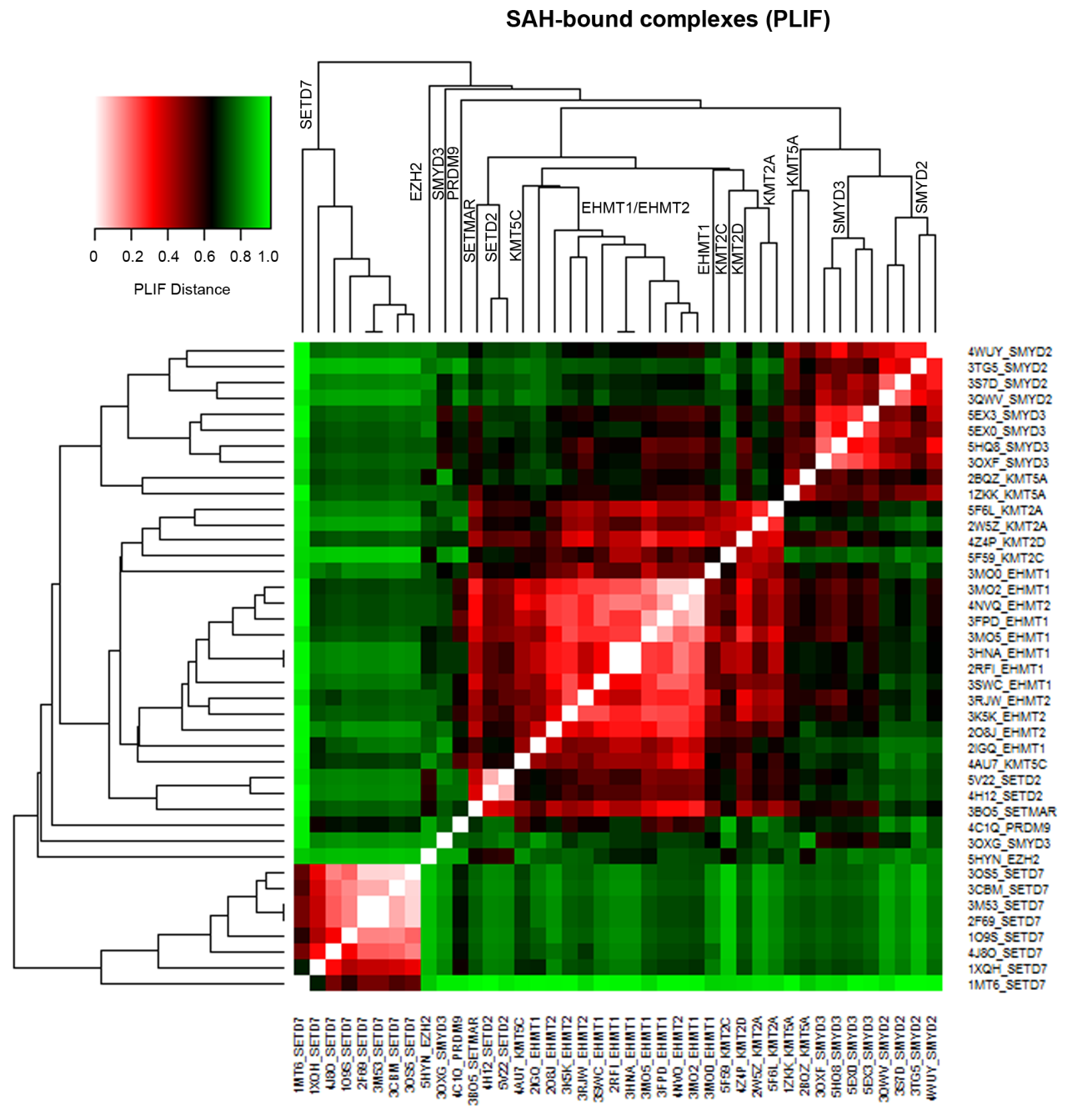


**Figure S11**. Heat map representation of PLIF similarities (expressed as distances) for the average of pairwise similarities of a particular PKMT for all the 101 PKMT – SAM binding complexes.


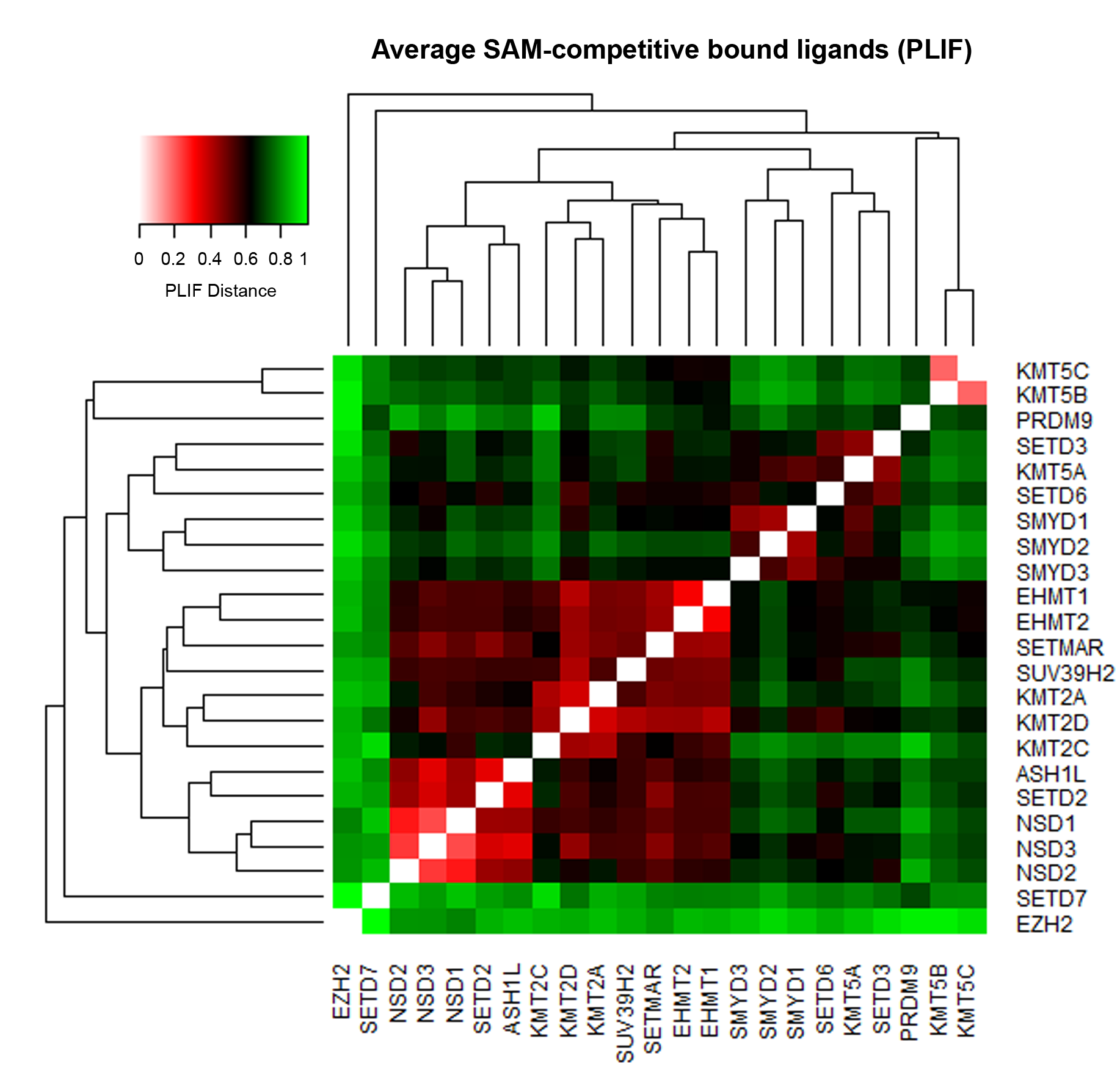


Phylogenetically related PKMTs mostly tend to cluster together (e.g. EHMT2/EHMT1; NSD1/NSD2/NSD3; SMYD2/SMYD3; KMT2C/KMT2D) with PLIF distance values < 0.4. Only a few unanticipated relations emerge, such as the group formed by KMT5A and SETD3 (distance of 0.46). EZH2, despite phylogenetic similarity with KMT2A/KMT2C/KMT2D (Fig. 1) is clustered independently, probably as a result of the different interaction pattern at residue alignment positions 1361, 1612, 1619 and different interactions of the ribose ring discussed in the manuscript. SETD7 clustering as a singleton mirrors its singularity in the phylogenetic tree in Fig. 1 as well as in the interactions in Fig. S5. PRDM9, the single representative crystal of the PRDM family, can also be regarded as a singleton, because of its high distance (>0.65) to the rest of PKMTs. The highest PLIF-based similarity region is observed among members of the SETD2 subfamily (ASH1L, NSDs, SETD2), MLL subfamily (KMT2s) and Suvar3-9 subfamily (EHMT1/EHMT2 and SUV39H2), recapitulating phylogenetic similarities.

**Table S7.** Distribution of PLIF detected contacts for the 33 complexes of SET domain-containing PKMTs bound with substrate-competitive inhibitors. % corresponds to # crystals divided by the number of complexes (33). HBD = Hydrogen Bond Donor. HBA = Hydrogen Bond Acceptor.

| Alignment Position | Contact Type | # Crystals | % | PKMTs involved |
| --- | --- | --- | --- | --- |
| 831 | HBD | 2 | 6.1 | SMYD2 |
| 972 | Surface Contacts | 4 | 12.1 | SMYD2 |
| 1063 | HBD | 12 | 36.4 | EHMT2/EHMT1/SETD7 |
| 1063 | Ionic | 5 | 15.2 | EHMT2/EHMT1 |
| 1063 | Surface Contacts | 5 | 15.2 | EHMT2/EHMT1 |
| 1087 | HBD | 3 | 9.1 | EHMT2 |
| 1087 | Surface Contacts | 3 | 9.1 | EHMT1/SETD7 |
| 1088 | HBD | 3 | 9.1 | SETD7 |
| 1088 | Ionic | 2 | 6.1 | SETD7 |
| 1089 | HBD | 14 | 42.4 | EHMT2/EHMT1 |
| 1097 | HBD | 9 | 27.3 | EHMT2/EHMT1 |
| 1114 | HBD | 6 | 18.2 | SMYD2 |
| 1114 | Surface Contacts | 6 | 18.2 | SMYD2 |
| 1124 | HBD | 21 | 63.6 | EHMT2/EHMT1/KMT5A/SMYD2 |
| 1124 | Surface Contacts | 3 | 9.1 | KMT5A/SETD7 |
| 1130 | HBD | 4 | 12.1 | EHMT2/EHMT1 |
| 1130 | Surface Contacts | 2 | 6.1 | KMT5A |
| 1130 | HBA | 4 | 12.1 | SMYD2/SMYD3 |
| 1147 | HBD | 8 | 24.2 | SMYD2/SMYD3 |
| 1148 | HBD | 14 | 42.4 | EHMT2/EHMT1/SETD7 |
| 1148 | Surface Contacts | 22 | 66.7 | EHMT2/EHMT1/SETD7/SMYD3/SMYD2 |
| 1148 | HBA | 5 | 15.2 | SMYD2/SMYD3 |
| 1148 | Arene Attraction | 7 | 21.2 | EHMT2/EHMT1/SMYD2 |
| 1149 | HBA | 3 | 9.1 | SETD7 |
| 1151 | HBD | 13 | 39.4 | EHMT2/EHMT1/SMYD2 |
| 1151 | HBA | 11 | 33.3 | SETD7/SMYD3/SMYD2 |
| 1151 | Surface Contacts | 6 | 18.2 | SMYD2 |
| 1151 | Arene Attraction | 2 | 6.1 | SMYD2 |
| 1197 | HBD | 2 | 6.1 | SMYD3 |
| 1208 | HBD | 2 | 6.1 | SMYD3 |
| 1256 | Surface Contacts | 2 | 6.1 | KMT5B |
| 1325 | HBD | 4 | 12.1 | EHMT2/EHMT1 |
| 1358 | HBD | 3 | 9.1 | KMT5A/SMYD2 |
| 1358 | HBA | 2 | 6.1 | SMYD3 |
| 1473 | HBD | 11 | 33.3 | EHMT2/SMYD3/SMYD2 |
| 1473 | Surface Contacts | 12 | 36.4 | EHMT2/SMYD3/SMYD2 |
| 1473 | Arene Attraction | 3 | 9.1 | EHMT2/EHMT1 |
| 1476 | HBD | 3 | 9.1 | SETD7 |
| 1476 | HBA | 3 | 9.1 | SETD7 |
| 1476 | Arene Attraction | 3 | 9.1 | SETD7 |
| 1496 | HBD | 4 | 12.1 | SMYD2 |
| 1576 | Surface Contacts | 8 | 24.2 | EHMT2/EHMT1 |
| 1585 | HBD | 4 | 12.1 | EHMT2/EHMT1 |
| **TOTAL** |  | **265** |  |  |

**Figure S12.** 2D schematic view of PLIF conserved contacts across the 33 complexes of substrate-competitive inhibitors against 7 different PKMTs (EHMT2, EHMT1, KMT5A, SETD7, SMYD2, SMYD3 and KMT5B). Circles and dotted lines correspond to hydrogen bond contacts and surface contact interactions (affecting different atoms), respectively. Residue numbers in the legend correspond to the alignment position of the corresponding amino acid residue in the PKMT alignment (not to the PDB number).


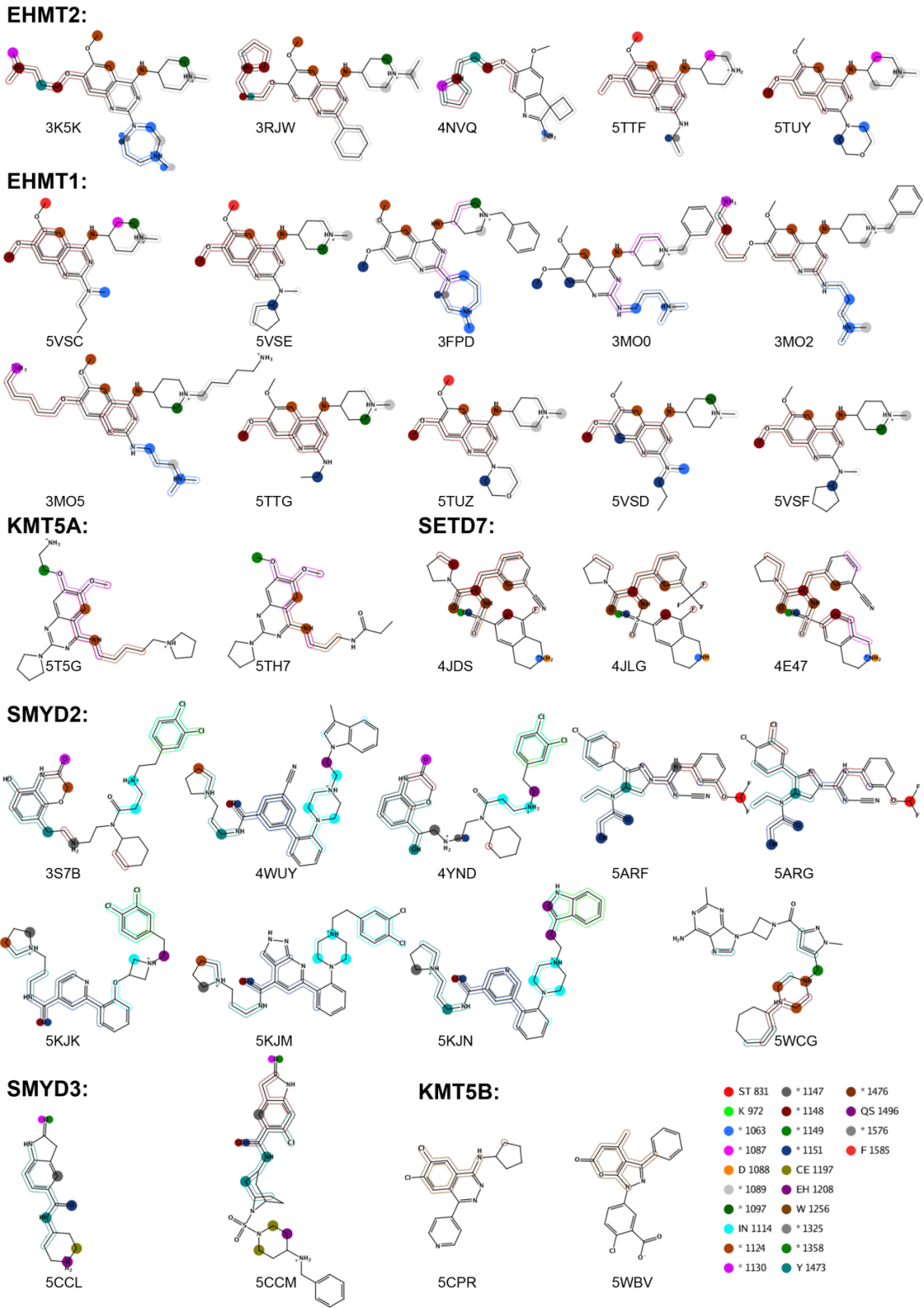


**Table S8.** Average RMSD (Cα and All atoms) among all 7 PKMTs with substrate competitive inhibitors.

| Protein | PDB entries | RMSD (Cα) | RMSD (All) | Selected Structure for Fig. 7 |
| --- | --- | --- | --- | --- |
| EHMT2 | 3K5K, 3RJW, 4NVQ, 5TTF, 5TUY, 5VSC, 5VSE | 0.502 | 0.957 | 3K5K |
| EHMT1 | 3FPD, 3MO0, 3MO2, 3MO5, 5TTG, 5TUZ, 5VSD, 5VSF | 0.752 | 1.262 | 3MO2 |
| KMT5A | 5T5G, 5TH7 | 2.522 | 2.750 | 5T5G |
| SETD7 | 4JDS, 4JLG, 4E47 | 0.468 | 1.017 | 4JDS |
| SMYD3 | 5CCL, 5CCM | 0.428 | 0.822 | 5CCM |
| SMYD2 | 3S7B, 4WUY, 4YND, 5ARF, 5ARG, 5KJK, 5KJM, 5KJN, 5WCG | 0.843 | 1.098 | 4YND |
| KMT5B | 5CPR, 5WBV | 0.682 | 1.266 | 5WBV |

**Figure S13.** FCFP_4-based similarities between the 30 substrate-competitive inhibitors of the 7 PKMTs. Chemical structure of 5TUY_EHMT2 = 5TUZ_EHMT1, 5VSC_EHMT2 = 5VSD_EHMT1, 5VSE_EHMT2 = 5VSF_EHMT1. For the pair 5TTG_EHMT1 and 5TTF_EHMT2, the X-ray resolved ligand structure does not match the original structures and differs between them.


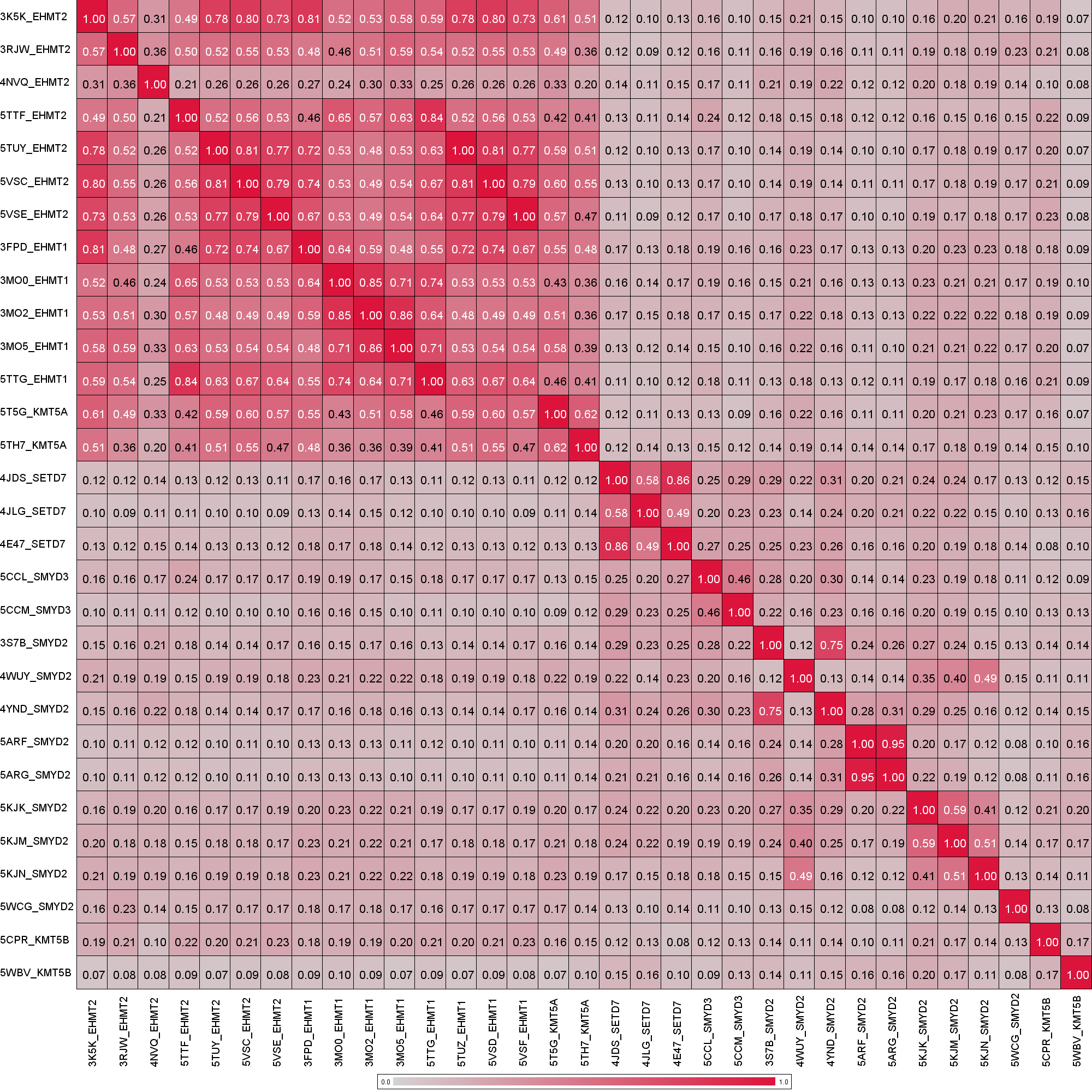


**Figure S14.** Heat map representation of PLIF similarities (expressed as distances) for the 33 substrate-competitive complexes.


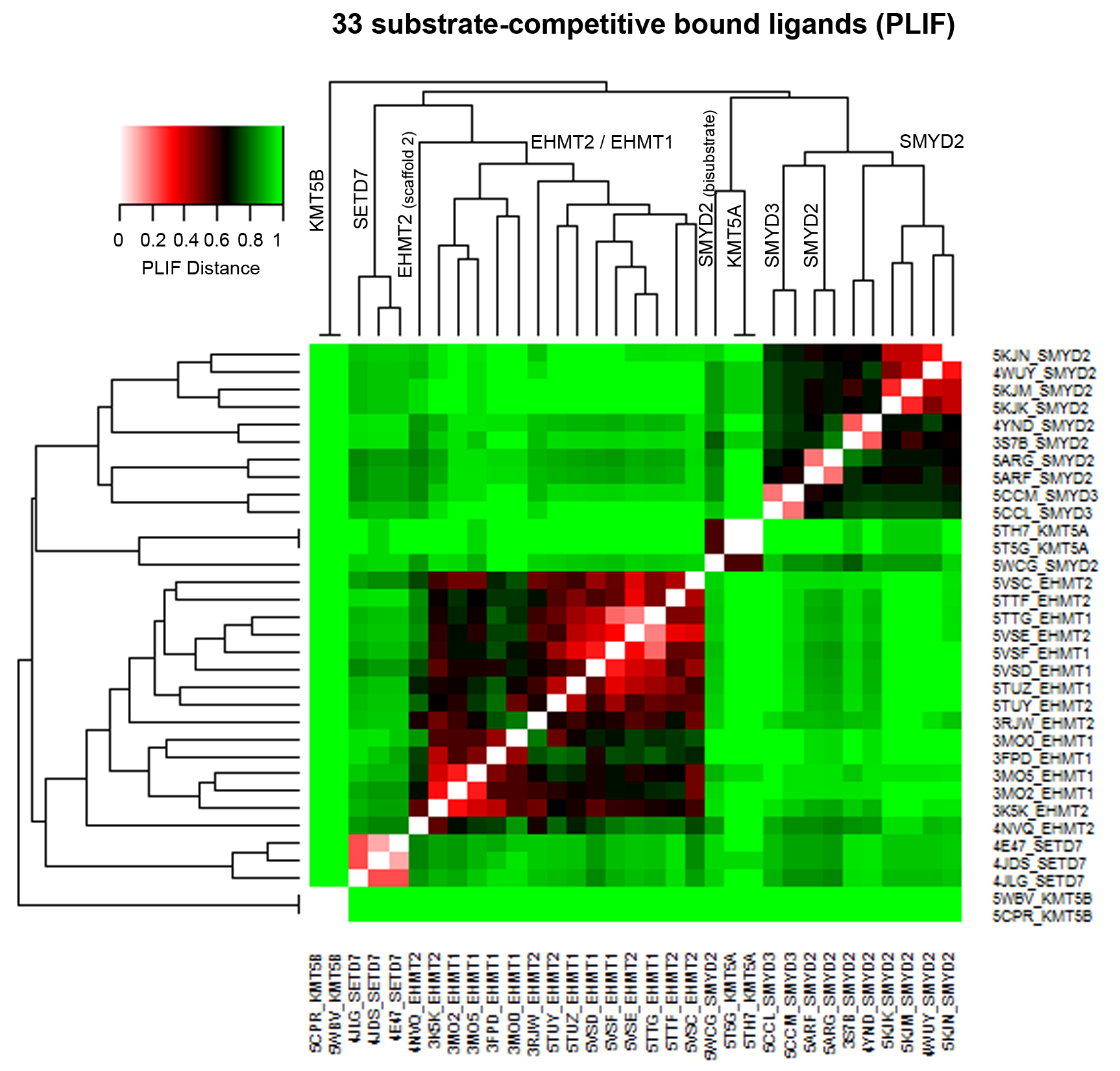


All crystals of a given PKMT cluster together, with the exception of the bisubstrate SMYD2 inhibitor MTF003 (PDB entry 5WCG), which groups with KMT5A inhibitors due to the fact that these three ligands enter depth into the lysine binding channel. Three main clusters are identified: 1) for KMT5B, fully dissimilar to any other PKMT (distance = 1) as expected from the analysis of the interactions of the A-196 inhibitor; 2) a higher cluster formed by SETD7, EHMT1 and EHMT2, in line with visual inspection of complexes in Fig. 7, although distances between SETD7 and EHMT1/EHMT2 are on average ~ 0.90 and 3) and a cluster formed by KMT5A and SMYD2/SMYD3 (again, but with a high average distance of 0.94 between these two subgroups).

**Figure S15.** Heat map representation of Gonnet similarity matrix (expressed as distances) for the SAM-binding site.


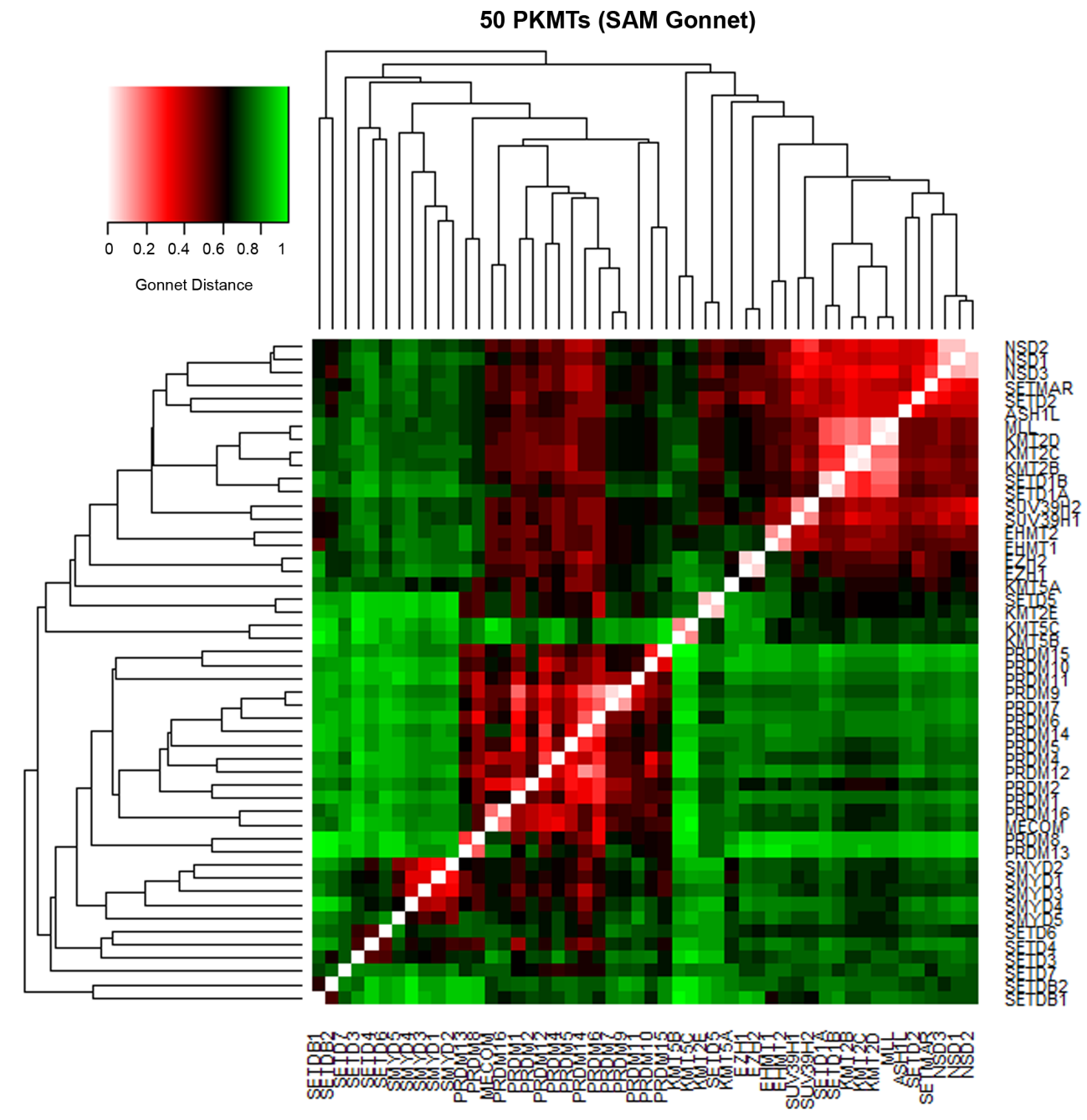


**Figure S16.** Heat map representation of Gonnet similarity matrix (expressed as distances) for the substrate-binding site.


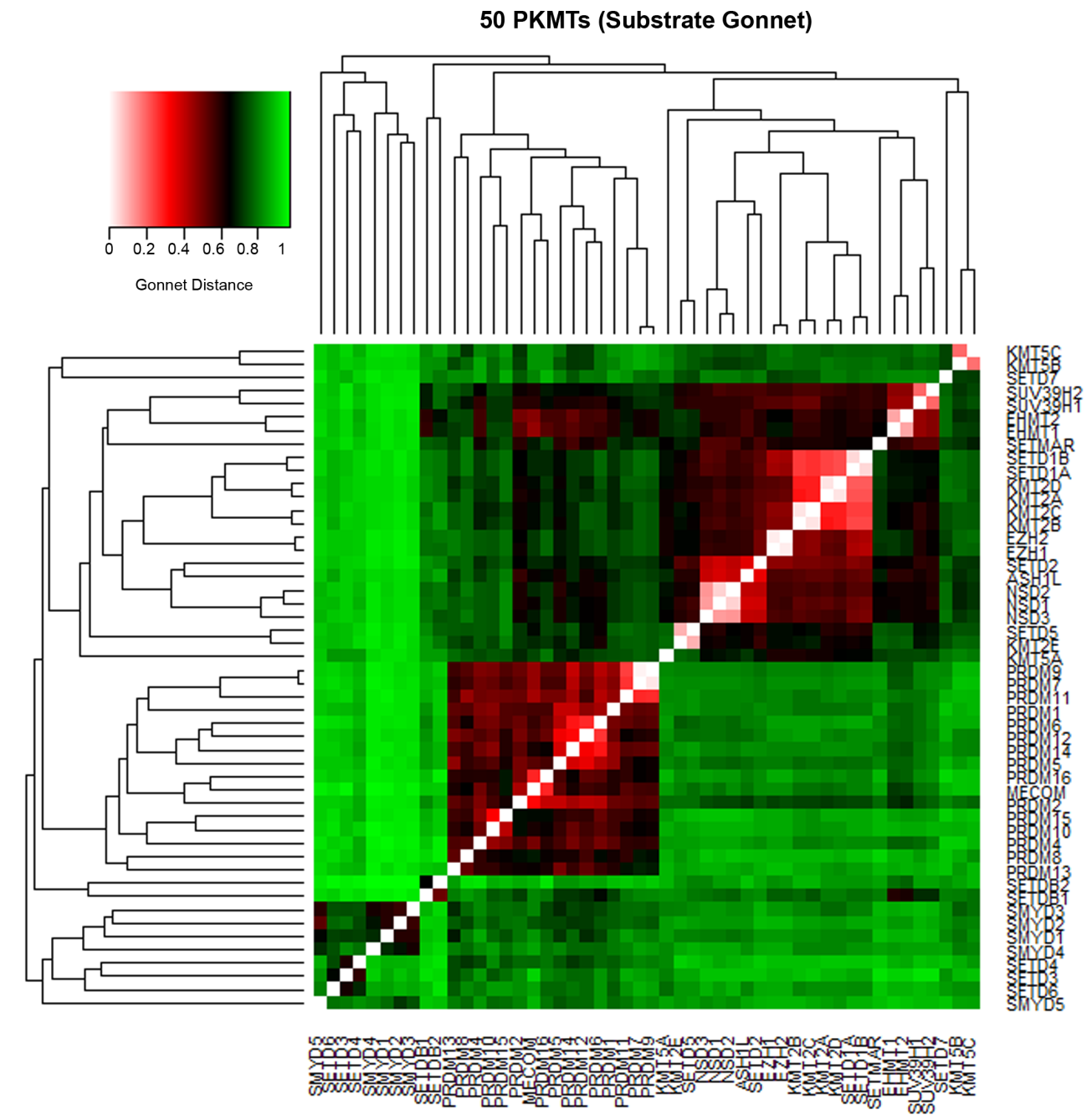


**Table S9.** Percentage of inhibition (mean values and two independent replicates) for the proprietary compounds CM-272, CM-986, CM-679. Assays carried out at Eurofins (<https://www.eurofins.com/>).

| **PKMT** | **Compound** | **Test Concentration (µM)** | **% Inhibition** | | |
| --- | --- | --- | --- | --- | --- |
|  |  |  | **1st** | **2nd** | **Mean** |
| SETD2 | CM-272 | 10 | -41.1 | -178.6 | -41.1 |
|  |  | 100 | 57.8 | 34.1 | 45.9 |
|  | CM-986 | 10 | -68.9 | -70.7 | -69.8 |
|  |  | 100 | 44.3 | 39.8 | 42.1 |
|  | CM-679 | 10 | -51.0 | -53.8 | -52.4 |
|  |  | 100 | 85.6 | 82.8 | 84.2 |
| KMT2A | CM-272 | 10 | -6.1 | 4.1 | -1.0 |
|  |  | 100 | -2.0 | 16.9 | 7.4 |
|  | CM-986 | 10 | -5.9 | 11.7 | 2.9 |
|  |  | 100 | 21.8 | 39.2 | 30.5 |
|  | CM-679 | 10 | 6.6 | 10.6 | 8.6 |
|  |  | 100 | 40.7 | 56.9 | 48.8 |
| KMT5C | CM-272 | 10 | 95.8 | 88.3 | 92.0 |
|  |  | 100 | 96.9 | 86.7 | 91.8 |
|  | CM-986 | 10 | 79.1 | 79.2 | 79.1 |
|  |  | 100 | 94.3 | 81.0 | 87.7 |
|  | CM-679 | 10 | 84.6 | 86.7 | 85.7 |
|  |  | 100 | 94.3 | 91.4 | 92.9 |
| NSD2 | CM-272 | 10 | -5.3 | 4.3 | -0.5 |
|  |  | 100 | 18.5 | 4.9 | 11.7 |
|  | CM-986 | 10 | -13.2 | -1.1 | -7.2 |
|  |  | 100 | 4.0 | 1.5 | 2.7 |
|  | CM-679 | 10 | 7.7 | 16.2 | 11.9 |
|  |  | 100 | 0.6 | 0.0 | 0.3 |
| NSD1 | CM-272 | 10 | 62.4 | 64.6 | 63.5 |
|  |  | 100 | 98.3 | 97.3 | 97.8 |
|  | CM-986 | 10 | 60.5 | 51.3 | 55.9 |
|  |  | 100 | 98.7 | 99.0 | 98.8 |
|  | CM-679 | 10 | 83.5 | 79.9 | 81.7 |
|  |  | 100 | 97.8 | 98.2 | 98 |

**References**

1. Brooun A, Gajiwala KS, Deng Y-L, et al (2016) Polycomb repressive complex 2 structure with inhibitor reveals a mechanism of activation and drug resistance. Nat Commun 7:11384

2. Vaswani RG, Gehling VS, Dakin LA, et al (2016) Identification of (R)-N-((4-Methoxy-6-methyl-2-oxo-1,2-dihydropyridin-3-yl)methyl)-2-methyl-1-(1-(1-(2,2,2-trifluoroethyl)piperidin-4-yl)ethyl)-1H-indole-3-carboxamide (CPI-1205), a Potent and Selective Inhibitor of Histone Methyltransferase EZH2, Suitabl. J Med Chem 59:9928–9941

3. Chang Y, Sun L, Kokura K, et al (2011) MPP8 mediates the interactions between DNA methyltransferase Dnmt3a and H3K9 methyltransferase GLP/G9a. Nat Commun 2:533

4. Subramanian K, Jia D, Kapoor-Vazirani P, Powell DR, Collins RE, Sharma D, Peng J, Cheng X, Vertino PM (2008) Regulation of estrogen receptor alpha by the SET7 lysine methyltransferase. Mol Cell 30:336–47

5. Tisi D, Chiarparin E, Tamanini E, et al (2016) Structure of the epigenetic oncogene MMSET and inhibition by N-alkyl sinefungin derivatives. ACS Chem Biol 11:3093–3105

6. Sirinupong N, Brunzelle J, Doko E, Yang Z (2011) Structural insights into the autoinhibition and posttranslational activation of histone methyltransferase SmyD3. J Mol Biol 406:149–59

7. Jiang Y, Sirinupong N, Brunzelle J, Yang Z (2011) Crystal structures of histone and p53 methyltransferase SmyD2 reveal a conformational flexibility of the autoinhibitory C-terminal domain. PLoS One 6:e21640

8. Sirinupong N, Brunzelle J, Ye J, Pirzada A, Nico L, Yang Z (2010) Crystal structure of cardiac-specific histone methyltransferase SmyD1 reveals unusual active site architecture. J Biol Chem 285:40635–44

9. Zheng W, Ibáñez G, Wu H, et al (2012) Sinefungin derivatives as inhibitors and structure probes of protein lysine methyltransferase SETD2. J Am Chem Soc 134:18004–14

10. Mori S, Iwase K, Iwanami N, Tanaka Y, Kagechika H, Hirano T (2010) Development of novel bisubstrate-type inhibitors of histone methyltransferase SET7/9. Bioorg Med Chem 18:8158–66

11. Van Aller GS, Graves AP, Elkins PA, et al (2016) Structure-Based Design of a Novel SMYD3 Inhibitor that Bridges the SAM-and MEKK2-Binding Pockets. Structure 24:774–781

12. Liu F, Chen X, Allali-Hassani A, et al (2009) Discovery of a 2,4-diamino-7-aminoalkoxyquinazoline as a potent and selective inhibitor of histone lysine methyltransferase G9a. J Med Chem 52:7950–3

13. Liu F, Chen X, Allali-Hassani A, et al (2010) Protein lysine methyltransferase G9a inhibitors: design, synthesis, and structure activity relationships of 2,4-diamino-7-aminoalkoxy-quinazolines. J Med Chem 53:5844–57

14. Chang Y, Zhang X, Horton JR, Upadhyay AK, Spannhoff A, Liu J, Snyder JP, Bedford MT, Cheng X (2009) Structural basis for G9a-like protein lysine methyltransferase inhibition by BIX-01294. Nat Struct Mol Biol 16:312–7

15. Vedadi M, Barsyte-Lovejoy D, Liu F, et al (2011) A chemical probe selectively inhibits G9a and GLP methyltransferase activity in cells. Nat Chem Biol 7:566–74

16. Morishita M, Mevius DEHF, Shen Y, Zhao S, di Luccio E (2017) BIX-01294 inhibits oncoproteins NSD1, NSD2 and NSD3. Med Chem Res 26:2038–2047

17. Kubicek S, O’Sullivan RJ, August EM, et al (2007) Reversal of H3K9me2 by a small-molecule inhibitor for the G9a histone methyltransferase. Mol Cell 25:473–81

18. Chang Y, Ganesh T, Horton JR, et al (2010) Adding a lysine mimic in the design of potent inhibitors of histone lysine methyltransferases. J Mol Biol 400:1–7

19. UNC0638 Selective chemical probe for G9a/GLP methyltransferases. http://www.thesgc.org/chemical-probes/UNC0638. Accessed 16 Dec 2016

20. Xiong Y, Li F, Babault N, et al (2017) Discovery of Potent and Selective Inhibitors for G9a-Like Protein (GLP) Lysine Methyltransferase. J Med Chem 60:1876–1891

21. Xiong Y, Li F, Babault N, et al (2017) Structure-activity relationship studies of G9a-like protein (GLP) inhibitors. Bioorganic Med Chem 25:4414–4423

22. Sweis RF, Pliushchev M, Brown PJ, et al (2014) Discovery and development of potent and selective inhibitors of histone methyltransferase g9a. ACS Med Chem Lett 5:205–9

23. A-366 A chemical Probe for G9a/GLP. http://www.thesgc.org/chemical-probes/A-366. Accessed 10 Oct 2017

24. Butler K V, Ma A, Yu W, et al (2016) Structure-Based Design of a Covalent Inhibitor of the SET Domain-Containing Protein 8 (SETD8) Lysine Methyltransferase. J Med Chem 59:9881–9889

25. Barsyte-Lovejoy D, Li F, Oudhoff MJ, et al (2014) (R)-PFI-2 is a potent and selective inhibitor of SETD7 methyltransferase activity in cells. Proc Natl Acad Sci 111:12853–12858

26. Nguyen KT, Li F, Poda G, Smil D, Vedadi M, Schapira M (2013) Strategy to target the substrate binding site of SET domain protein methyltransferases. J Chem Inf Model 53:681–91

27. Mitchell LH, Boriack-Sjodin PA, Smith S, et al (2016) Novel Oxindole Sulfonamides and Sulfamides: EPZ031686, the First Orally Bioavailable Small Molecule SMYD3 Inhibitor. ACS Med Chem Lett 7:134–138

28. Ferguson AD, Larsen NA, Howard T, et al (2011) Structural basis of substrate methylation and inhibition of SMYD2. Structure 19:1262–1273

29. Nguyen H, Allali-Hassani A, Antonysamy S, et al (2015) LLY-507, a cell-active, potent, and selective inhibitor of protein-lysine methyltransferase SMYD2. J Biol Chem 290:13641–13653

30. LLY-507 A chemical probe for SMYD2 protein lysine methyltransferase. http://www.thesgc.org/chemical-probes/LLY-507. Accessed 10 Oct 2017

31. Sweis RF, Wang Z, Algire M, et al (2015) Discovery of A-893, A New Cell-Active Benzoxazinone Inhibitor of Lysine Methyltransferase SMYD2. ACS Med Chem Lett 6:695–700

32. Eggert E, Hillig RC, Koehr S, et al (2016) Discovery and Characterization of a Highly Potent and Selective Aminopyrazoline-Based in Vivo Probe (BAY-598) for the Protein Lysine Methyltransferase SMYD2. J Med Chem 59:4578–600

33. BAY-598 A selective chemical probe for SMYD2. http://www.thesgc.org/chemical-probes/BAY-598. Accessed 10 Oct 2017

34. Cowen SD, Russell D, Dakin LA, et al (2016) Design, Synthesis, and Biological Activity of Substrate Competitive SMYD2 Inhibitors. J Med Chem 59:11079–11097

35. Bromberg KD, Mitchell TRH, Upadhyay AK, et al (2017) The SUV4-20 inhibitor A-196 verifies a role for epigenetics in genomic integrity. Nat Chem Biol 13:317–324

36. A-196 A selective chemical probe for SUV420H1/H2. http://www.thesgc.org/chemical-probes/A-196. Accessed 10 Oct 2017

37. SCG. ChromoHub: a data hub for navigators of chromatin-mediated signalling. http://apps.thesgc.org/resources/phylogenetic_trees/index.php. Accessed 25 Sep 2017

38. Takemoto Y, Ito A, Niwa H, et al (2016) Identification of Cyproheptadine as an Inhibitor of SET Domain Containing Lysine Methyltransferase 7/9 (Set7/9) That Regulates Estrogen-Dependent Transcription. J Med Chem 59:3650–60

39. Yuan Y, Wang Q, Paulk J, Kubicek S, Kemp MM, Adams DJ, Shamji AF, Wagner BK, Schreiber SL (2012) A small-molecule probe of the histone methyltransferase G9a induces cellular senescence in pancreatic adenocarcinoma. ACS Chem Biol 7:1152–7

40. Meng F, Cheng S, Ding H, et al (2015) Discovery and Optimization of Novel, Selective Histone Methyltransferase SET7 Inhibitors by Pharmacophore- and Docking-Based Virtual Screening. J Med Chem 58:8166–81

41. Kashyap S, Sandler J, Peters U, Martinez EJ, Kapoor TM (2014) Using “biased-privileged” scaffolds to identify lysine methyltransferase inhibitors. Bioorg Med Chem 22:2253–60

42. Verma SK, Tian X, LaFrance L V., et al (2012) Identification of Potent, Selective, Cell-Active Inhibitors of the Histone Lysine Methyltransferase EZH2. ACS Med Chem Lett 3:1091–1096

43. Konze KD, Ma A, Li F, et al (2013) An orally bioavailable chemical probe of the Lysine Methyltransferases EZH2 and EZH1. ACS Chem Biol 8:1324–34

44. Xu S, Wu J, Sun B, Zhong C, Ding J (2011) Structural and biochemical studies of human lysine methyltransferase Smyd3 reveal the important functional roles of its post-SET and TPR domains and the regulation of its activity by DNA binding. Nucleic Acids Res 39:4438–49
